# A survey of quality of life indicators in the Romanian Roma population following the ‘Decade of Roma Inclusion’

**DOI:** 10.12688/f1000research.12546.3

**Published:** 2018-12-13

**Authors:** Rebecca Powell Doherty, Pyrros A. Telionis, Daniel Müller-Demary, Alexandra Hosszu, Ana Duminica, Andrea Bertke, Bryan Lewis, Stephen Eubank

**Affiliations:** 1 Biocomplexity Institute of Virginia Tech, Blacksburg, VA, USA; 2 Population Health Sciences, Virginia Tech, Blacksburg, VA, USA; 3 Department of Geography, Virginia Tech, Blacksburg, VA, USA; 4 Agentia Impreuna, Bucharest, Romania

**Keywords:** Roma, Romania, rural populations, water quality, healthcare, development, global health, decade of Roma inclusion

## Abstract

**Background**: This study explores how the Roma in Romania, the EU’s most concentrated population, are faring in terms of a number of quality of life indicators, including poverty levels, healthcare, education, water, sanitation, and hygiene. It further explores the role of synthetic populations and modelling in identifying at-risk populations and delivering targeted aid.

**Methods**: 135 surveys were conducted across five geographically diverse Romanian communities. Household participants were selected through a comprehensive random walk method. Analyses were conducted on all data using Pandas for Python. Combining land scan data, time-use survey analyses, interview data, and ArcGIS, the resulting synthetic population was analysed via classification and regression tree (CART) analysis to identify hot-spots of need, both ethnically and geographically.

**Results**: These data indicate that the Roma in Romania face significant disparities in education, with Roma students less likely to progress beyond 8 th grade. In addition, the Roma population remains significantly disadvantaged with regard to safe and secure housing, poverty, and healthcare status, particularly in connection to diarrheal disease. In contrast, however, both Roma and non-Roma in rural areas face difficulties regarding full-time employment, sanitation, and water, sanitation, and hygiene infrastructure. In addition, the use of a synthetic population can generate information about ‘hot spots’ of need, based on geography, ethnicity, and type of aid required.

**Conclusions**: These data demonstrate the challenges that remain to the Roma population in Romania, and also point to the myriad of ways in which all rural Romanians, regardless of ethnicity, are encountering hardship. This study highlights an approach that combines traditional survey data with more wide-reaching geographically based data and CART analysis to determine ‘hot spot’ areas of need in a given population. With the appropriate inputs, this tool can be extrapolated to any population in any country.

## Introduction

In the years that followed independence and the democratic election of 1990, the southeastern European country of Romania received significant aid from the International Monetary Fund (IMF), World Bank (WB), European Bank for Reconstruction and Development (EBRD), European Investment Bank (EIB), the US Agency for International Development (USAID), and other donors ^1^. This influx of investment enabled Romania to make great strides in multiple areas of development and meet a number of the goals set forth in the United Nations Millennium Development Goals (UN MDGs) ^2^. In particular, the issues of severe poverty and hunger have significantly improved for ethnic Romanians and affluent minorities, with severe poverty (as defined by the United Nations) decreasing from 10 per cent to 4.1 per cent as of 2006 ^2^. In addition, maternal mortality has fallen by half to 17 deaths/100,000 births, infant mortality has decreased 25 per cent, and Romania has seen a significant decrease in adolescent pregnancy, concomitant with a significant increase in the use of modern contraceptives ^2^. In the 1990’s and early 2000’s, vaccination rates, particularly for measles, improved to around 98 per cent, up from less than 70 per cent at the time of independence; HIV/AIDS cases have decreased and life expectancy for those living with HIV has increased dramatically; and there has been a significant decrease in domestic violence ^2^.

For the Roma, the second most numerous minority in the country (after Hungarians), however, such progress was not extended. Despite enjoying a reprieve from targeted discrimination during the Soviet era, Romanian independence brought on a renewal of oppressive policies and behaviours against the Roma. The Roma are Europe’s most marginalised group ^3^, a minority population numbering between 10–12 million individuals across the continent and the UK ^4^. Emerging from slavery in the late 19 ^th^ century, they have historically faced discrimination in employment, education, and access to healthcare ^5^. Numerous studies indicate Roma have a significantly reduced lifespan compared to non-Roma and suffer greater rates of communicable and waterborne diseases ^6– 8^. In multiple countries, they are less likely to have access to basic services, including a municipal water supply, waste water treatment, or trash disposal ^9^,. Romania boasts the largest concentration of Roma in the European Union (EU), at approximately 1.85 million individuals, representing 9.3 per cent of the overall population of 19.8 million, though official census numbers vary ^4^.

The addition of eastern European countries (including Bulgaria, Romania, and Hungary) to the EU in the mid-2000s has renewed interest in the well-being of this population, as indicated by the EU’s targeted attempt to improve the circumstances of the Roma through the recently concluded *Decade of Roma Inclusion* (DRI), a ten year long initiative by twelve European countries to improve the socio-economic status and social standing of the Roma minority across the continent ^10^. Numerous studies have explored the success of the DRI, both during its implementation and since its conclusion, and outcomes vary, depending on the sector and goal in question ^8, 11– 13^. 

For such assessments, international aid agencies and non-governmental organizations often employ assessment surveys and interviews to determine the type and level of need in a particular area or for a disadvantaged population ^14^. However, while such methods are useful for specific communities ‘of interest’ and can provide statistical support for straightforward claims or goals, they are of little use in identifying new areas and populations in need or addressing multi-faceted and complex issues. This proof-of-concept study explores the possibility of using a synthetic representation of Romania (down to the individual level) to predict currently unrecognized areas of need based on key variables from an assessment survey and a classification and regression tree (CART) analysis. The synthetic population was generated from the fusion of land-scan data, geographic census data and ethnicity statistics, time-use surveys, and our own needs assessment survey data. Our representation captures details about households and their quality of life, and is able to capture heterogeneities across geographic space. This approach augments the strength of the survey, in particular allowing the identification of potential areas of need without requiring additional resources to conduct a needs assessment in those regions. Furthermore, the synthetic population becomes an ideal foundation for dynamic simulations and can be used to identify sub-populations at greatest risk for infection during disease outbreaks.

## Methods

### Regional survey

We developed our survey by combining questions adapted from a validated WASH survey previously used for multiple use service strategy research (personal communication to *authors*) and the WHO core questions on drinking-water and sanitation ^15^ with questions related to demographics, socio-economic status, and healthcare access and history, we conducted 135 surveys each consisting of 56 total questions across five geographically diverse communities throughout Romania. The survey questions were modified with the assistance of our NGO partner to appropriately reflect cultural characteristics in Romania. Communities were chosen from a list of those that had previously participated with *Agentia Impreuna* in education and anti-discrimination capacity-building programs for communities with prominent Roma populations. In addition, in an attempt to address geographical bias in improve the generalizability of our findings, communities were identified for their geographical diversity. Participating communities included central urban households, suburban communities, and very rural, mountainous regions. Communities were further distinct in the level of integration observed between the Roma population and the non-Roma, being fully integrated in some areas and completely separate in others. Household participants were selected through a comprehensive random walk method, with survey teams accompanied by both Roma and non-Roma community leaders. Survey teams varied the time of day they moved through any given community to ensure access to the full population, and interviews were conducted in areas throughout the community, with participants identified at their homes, as well as in shops and cafes. Identifying information for the participants was used only to ensure there was no duplication of household information. Any household with an individual over the age of 18 present and willing to participate, regardless of ethnicity, was included until the desired 30 surveys per community were achieved or there were no further willing participants. Interviews were conducted by trained volunteers who either spoke the national language (Romanian) or were accompanied by a certified translator. The team interviewed only one member of each household, who provided information about all members of the household. The specifics of participating communities are purposefully withheld to comply with the approval constraints of our ethics board.

### Ethical statement

Surveys ( Supplementary material 1 and Supplementary material 2) and procedures were approved by the Virginia Tech Institutional Review Board (IRB) prior to study implementation (VT IRB #16-475), and all interviews and analysis were carried out according to IRB protocol.

### IRB protocol and participant protections

Informed consent was obtained from all individual participants included in this study. A brief explanation of the survey questions and the intended use of the data was provided to each participant, and the individual’s agreement to participate in the survey interview was considered consent, as indicated by the IRB protocol. Further, interviewers ensured each participant understood that he or she could refuse to answer any question and could withdraw their consent at any time. Survey participation was anonymous, and no identifying information was retained. In addition, the IRB stipulated that location data for the participating villages remain unavailable, due to the vulnerable population and minority status of some study participants. All demographic information was self-reported, and those who were considered part of the Roma sample self-identified as either Roma or Rudar (a sub-set of Roma people who do not speak Romani), in response to a question that explicitly asked for their ethnicity ( Dataset 1).

**Figure 1.**
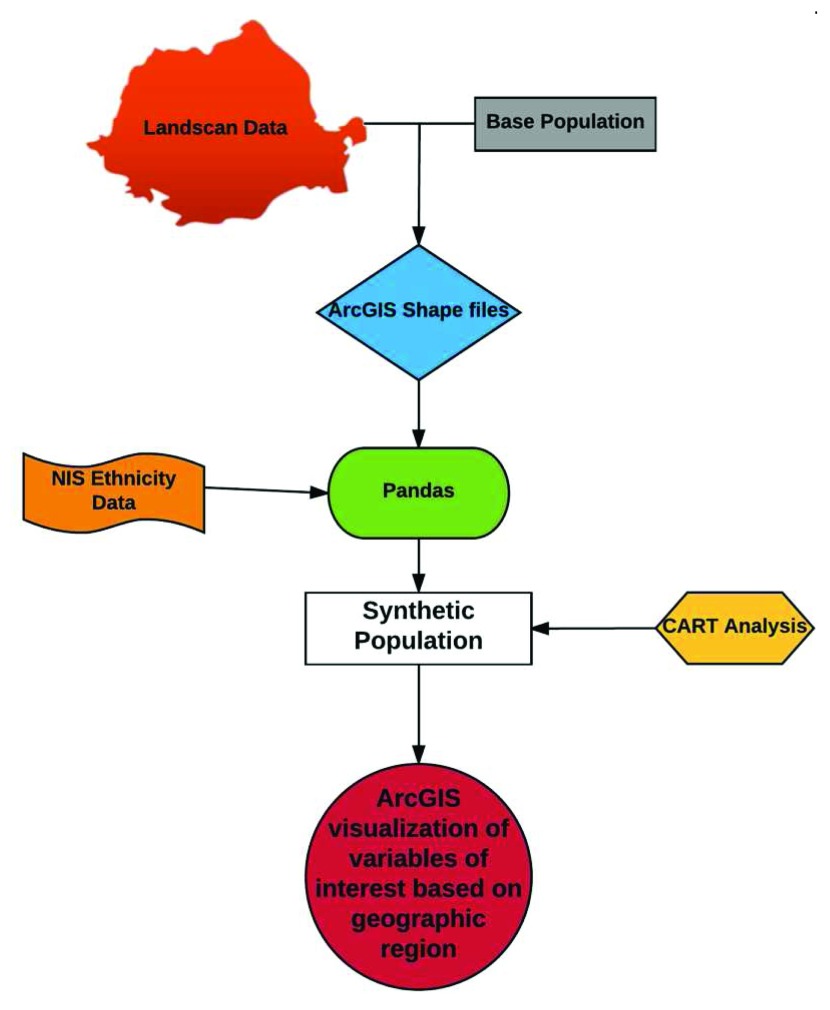
Work Flow to Generate Synthetic Population and ArcGIS mapping.

**Figure 2.**
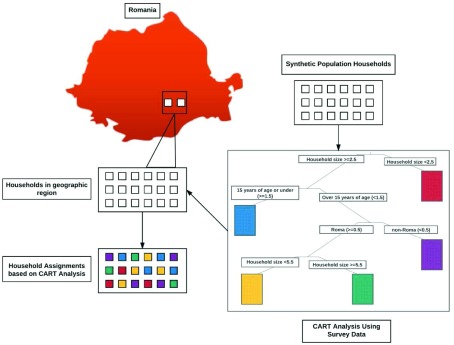
Work-flow for CART Analysis and categorical variable assignment in synthetic population.

***Synthetic Population Generation*.** In order to generate a synthetic population for Romania that would allow us to explore variables of interest based upon geographic location and ethnicity, we fused data sets from multiple sources ( Figure 1). To establish our base population, we populated the land-scan data from the Global Population Project ^16^ with data from the U.S. Census Bureau International Database ^17^, which predicts global populations based on past census data and growth projections, along with time-use survey data from Russia (chosen as a substitute for specific similarities) ^18^, as there are no available time-use data from Romania ^18^. We then used ArcGIS ^19^ to join this population to shape files that defined administrative regions of Romania, at the *judet* (county), city, town, and commune level ^20^. Exporting our population, now defined geographically, to Python/Pandas ^21^, we merged it by geographic region to ethnicity data, counts of individuals reporting to be from various ethnic groups, obtained from the Romanian National Institute of Statistics ^22^. We were then able to assign each household in the population an ethnicity (Roma or non-Roma), and identify regions of the country with concentrated Roma populations. Finally, we applied a CART analysis ( Figure 2), based upon our pilot survey data, to the synthetic population, and exported data related to our variables of interest (insecure housing, education level, water quality, diarrheal rates, parameters of poverty, and urban *versus* rural communities) to ArcGIS for visualization.

***Classification and Regresssion Tree (CART) Analysis*.** Using the synthetic population described, we used a classification and regression tree (CART) analysis to identify how ethnicity, household size, and age structure of the household predicted the responses to seven of the most significant quality of life indicators The resulting tree grouped many of the surveys into similar pools based on these three predictor values ( Figure 2). Acknowledging the small sample size of our pilot survey (n=135), we aggregated categories containing only a single household into four larger groupings. Using independent variables previously identified, the univariate classification tree produced five overall categories based on this analysis. Each household in the population was then assigned an individual survey response from its corresponding pool based on the ethnicity, household size, and age structure of the household.

### Primary data analysis

All data analyses were conducted via Pandas with Python (version 2.7.11 & 0.18.0) notebook and the software package Epipy ^21, 22^ ( Dataset 2– Dataset 3). Descriptive statistics were broken down by community, ethnicity, gender, age, household size, education level, marital status, employment, literacy, and geographical description (urban *versus* rural). WASH parameters were defined using the UN descriptions as provided in the DRI progress report through 2013, as well as the addition of a ‘safe water score’, which included the option of a private, protected well water source in addition to tap water in the home ^10^. The overall WASH score for each participating household is an aggregate of the following UN parameters: indoor toilet (improved sanitation), indoor bathroom (improved sanitation II), piped water to tap (improved water source), and insecure housing (a 0–3 score reflecting the status of the floor, walls, and roof of a dwelling). The overall ‘WASH Safe’ score exchanged the improved water source parameter for the aforementioned safe water score. In addition, time to primary drinking water sources has been converted to a numerical scale, based on 15 minute intervals, up to one hour (0–4 scale). Distance to primary drinking water is indicated both by a percentage of those in each ethnic group who travel a kilometre or more and the average distance travelled by each group. Similar to the WASH score, the healthcare score is an aggregate of self-reported immunization, reported incidence of diarrheal event, access to primary care physician (PCP), and reported medical insurance status. Finally, the poverty score is an aggregate of available electricity in dwelling, available gas source in dwelling, and the UN indicator of severe poverty (surviving on 2USD/person/day or less). Univariate analyses compared the Roma sample to the non-Roma sample for each variable (using non-Roma as the reference population), as well as urban areas to rural ones (with urban areas as the reference population) for some parameters. Odds Ratios (ORs) with 95 per cent confidence intervals are reported, as are t-test results (95 per cent confidence interval) with accompanying p-value where appropriate.

### Secondary data analysis and multivariate models

Multivariate linear regression analyses were conducted by using combinations of the four aggregate scores, as explained in primary analysis, and by including parameters that demonstrated significance in univariate modelling ( Dataset 2– Dataset 3).

***Hot Spot Generation*.** Using the Spatial Autocorrelation (Global Moran's I) tool in ArcGIS, which measures spatial autocorrelation based on feature locations and feature values, we analyzed each variable of interest to determine whether the pattern expressed in our population was random. Significant autocorrelation (non-random pattern or clustering) was determined by z-score and accompanying p-value (≤ 0.05). Significance or lack thereof suggests whether the independent variables upon which our synthetic population was built (household size and ethnicity) are appropriate indicators for our specific quality of life (QoL) parameters. For variables demonstrated to be significantly spatially auto-correlated, we progressed to Incremental Spatial Autocorrelation with a fixed distance measure to identify areas of intense need or ‘hot spot’ clustering. 

## Results

### Population demographics

Analyses of demographic data and breakdown by percentage indicate our sample population is, overall, predominantly Roma (72.6 per cent *vs.* 27.4 per cent non-Roma), split evenly by sex (50.4 per cent Female, 49.6 per cent Male), and average approximately 47 years of age ( Table 1). Three of the five sample communities are rural (more than 25km from a city centre), one is suburban (between 10–25km from a city centre), and one is urban (less than 10km from a city centre). There is no significant difference between Roma and non-Roma in the sample population on the basis of marital status, age, or sex. However, our data indicate notable disparities in level of education (secondary school completion for Roma *vs.* high school completion for non-Roma), household size (5.3 individuals for Roma *vs.* 4.2 individuals for non-Roma), and literacy rate (61 per cent literate Roma *vs.* 97.4 per cent literate non-Roma) ( Table 1). Little difference is noted in full-time employment rates between the groups (26.6 per cent Roma *vs.* 32.4 per cent non-Roma), though some difference is observable between rural and urban communities ( Table 1).

**Table 1.** Study population demographics broken down by community. Romania, 2016. *M=male, F=female, FT=full-time, UE=unemployed, DL=day labour*.

	*Population* *N (%)*	*Sex* *% M(F)*	*Age of* *Respondents* *(Mean in* *years)*	*House-hold* *Size N* *(Mean no.* *persons)*	*Education Level* *of Respondents* *(Mean Grade* *Completed)*	*Marital Status* *% Partnership* *(Single)*	*Employment* *Status % FT* *(UE or DL)*	*Literacy of* *Families* *Overall (%)*	*Geographical* *Location*
**Community 1**									
*Roma*	*28 (96.6)*	*28.5 (71.4)*	*49.3*	*4.7*	*Secondary* *School (8th* *grade)*	*71.4 (28.6)*	*10.7 (89.3)*	*56*	--
*Non-Roma*	*1 (3.4)*	*100 (0)*	*52*	*5*	*Some University* */ College*	*100 (0)*	*0 (100)*	*80*	--
Overall	29	31 (69)	49.4	4.7	Secondary School (8th grade)	72.4 (27.6)	7.7 (89.7)	57.5	*Rural*
**Community 2**									
*Roma*	*24 (80)*	*75 (25)*	*48.1*	*5.9*	*Secondary* *School (8th* *grade)*	*87.5 (12.5)*	*16.7 (83.3)*	*58.9*	--
*Non-Roma*	*6 (20)*	*50 (50)*	*50.1*	*4.8*	*Some University* */ College*	*83.3 (16.7)*	*66.7 (33.3)*	*100*	--
Overall	30	70 (30)	45.8	5.7	Required School (10th grade)	86.7 (13.3)	*26.7 (73.3)*	66.7	*Rural*
**Community 3**									
*Roma*	*18 (60)*	*44.4 (55.6)*	*42.6*	*5.3*	*Secondary* *School (8th* *grade)*	*94.4 (5.6)*	*27.8 (72.2)*	*58.3*	--
*Non-Roma*	*12 (40)*	*58.3 (41.7)*	*50.1*	*4.2*	*High School* *+ Vocational* *School*	*91.7 (8.3)*	*25 (75)*	*100*	--
Overall	30	50 (50)	45.6	4.9	Required School (10th grade)	93.3 (6.7	26.7(73.3)	74.4	*Suburban*
**Community 4**									
*Roma*	*13 (43.3)*	*46.2 (53.8)*	*42.7*	*6.1*	*Secondary* *School (8th* *grade)*	*84.6 (15.4)*	*23.1 (76.9)*	*48*	--
*Non-Roma*	*17 (56.7)*	*29.4 (70.6)*	*60.4*	*2.9*	*Required School* *(10th grade)*	*58.8 (41.2)*	*23.5 (76.5)*	*95.1*	--
Overall	30	36.7 (63.3)	52.7	4.3	Secondary School (8th grade)	70 (30	23.3 (76.7)	69.2	*Rural*
**Community 5**									
*Roma*	*15 (93.7)*	*66.7 (33.3)*	*35.4*	*4.7*	*High School* *(12th grade)*	*73.3 (26.7)*	*73.3 (26.7)*	*93.2*	--
*Non-Roma*	*1 (6.3)*	*100 (0)*	*40*	*4*	*Required School* *(10th grade)*	*100 (0)*	*100 (0)*	*100*	--
Overall	16	68.8 (31.2)	35.7	4.7	High School (12th grade)	75 (25)	75 (25)	93.6	*Urban*
**Overall**									
*Roma*	*98 (72.6)*	*51 (49)*	*44.8*	*5.3*	*Secondary* *School (8th* *grade)*	*75.5 (24.5)*	*26.6 (73.4)*	*61*	--
*Non-Roma*	*37 (27.4)*	*46 (54)*	*52.4*	*4.2*	*High School* *(12th grade)*	*75.6 (24.3)*	*32.4 (67.6)*	*97.4*	--
Total	135	49.6 (50.4)	46.9	4.8	Required School (10th grade)	80 (20)	28.1 (71.9)	72.3	--

### WASH, healthcare, poverty parameters

Using parameters utilized by the DRI in the 2011 progress report, univariate analysis indicates little difference between Roma and non-Roma with regard to specific WASH variables. The non-Roma are slightly more likely to have an indoor toilet (21.6 per cent non-Roma *vs* 17.3 per cent Roma) and bathroom (21.6 per cent non-Roma *vs* 20.4 per cent Roma), but the Roma are more likely than non-Roma to have tap (indoor or outdoor) water (20.4 per cent Roma *vs* 8.1 per cent non-Roma), whether piped in from a personal well or a municipal water source ( Table 2). However, when considering all safe water options (including a protected well *without* a tap to the home or garden), non-Roma report greater accessibility (59.5 per cent non-Roma *vs* 50 per cent Roma). In addition, Roma are significantly more at risk to inhabit insecure housing, regardless of geographical region, than non-Roma (27.6 per cent Roma *vs* 5.4 per cent non-Roma) ( Table 2). Interestingly, while the Roma population have greater access to tap water (indoor or outdoor), they are less likely to use it as their primary drinking water source, demonstrated by the increased time and distance Roma are likely to travel to secure safe drinking water (12.2km Roma *vs.* 10.8km non-Roma; Table 2). Of interest, however, is the increased time all individuals in suburban and urban areas must travel to secure drinking water compared to their rural counterparts (16–30 minutes (1.2 on 0–3 scale) urban *vs.* 0–15 minutes (1.0 on 0–3 scale) rural) ( Table 3).

**Table 2.** Univariate analyses. Romania, 2016. Reference population for all variables is non-Roma. * indicates significance at 95% CI level. ** indicates significance at 90% CI level.

		**Roma**	**Non-Roma**	**t-statistic**	**p-value**	**Odds** **Ratio**	**95% CI**
**WASH**	Improved Sanitation (Indoor Toilet, % yes)	17.3	21.6	0.567	0.57	1.31	0.51, 3.37
	Improved Sanitation II (Indoor Bathroom, % yes)	20.4	21.6	0.154	0.878	1.08	0.43, 2.71
	Improved Water Source (Piped water to tap, % yes)	20.4	8.1	-1.701	0.091 ^**^	0.34	0.1, 1.24
	Insecure Housing (% yes)	27.6	5.4	2.858	0.005 ^*^	6.65	1.5, 29.6
	Time to Primary Drinking Water Source (Mean, 0–4 scale, 15min intervals)	1.12	1.0	0.769	0.443	1.12	0.37, 3.43
	Distance to Primary Drinking Water Source (%, 1km or more)	12.2	10.8	0.124	0.901	1.15	0.35, 3.82
	Safe Water Source (tap or well, % yes)	50	59.5	0.978	0.329	1.47	0.8, 1.91
**Healthcare**	Moderate/Severe Diarrhea in Last Year (% yes)	58.1	40.5	-1.84	0.07 ^**^	2.04	0.94, 4.4
	Reports Immunization of any kind (% yes)	87.8	97.1	0.678	0.499	1.58	0.42, 5.96
	Medically Insured (% yes)	81.6	89.1	1.057	0.292	1.86	0.58, 5.9
	Access to PCP (% yes)	98	97	-0.231	0.818	0.75	0.07, 8.53
**Poverty**	Electricity in Home or Dwelling (% no)	13.2	2.7	1.804	0.07 ^**^	5.51	0.69, 43.68
	Piped or Tank Gas in Home or Dwelling (% no)	32.7	18.9	1.57	0.12	2.47	0.82, 5.24
	Spends more than $2/person/day (% no)	55.1	43.2	1.23	0.22	1.61	0.75, 3.45

**Table 3.** Geographical univariate analysis. Romania, 2016. Reference population for all variables is urban. * indicates significance at 95% CI level.

	*Rural*	*Urban*	*t-statistic*	*p-value*	*Odds* *Ratio*	*95% CI*
*Time to Primary Drinking Water Source* *(Mean, 0–4 scale, 15min intervals)*	*1.0*	*1.2*	*1.306*	*0.194*	*0.53*	*0.19, 1.49*
*Spends more than $2/person/day (% no)*	*61.8*	*32.6*	*3.323*	0.001 ^*^	*3.3*	*1.58, 7.08*

In addition to physical infrastructure, we analysed the differences between Roma and non-Roma with regard to key factors contributing to overall health status. Roma are more than twice as likely to report at least one household member suffering from moderate to severe diarrhoea (lasting more than 3 days) than non-Roma (58.1 per cent Roma *vs* 40.5 per cent non-Roma; OR 2.04) ( Table 2). In addition, while there is little difference in access to a primary care physician between the groups, Roma are approximately 1.5 times less likely to report having received an immunization of any kind (87.8 per cent Roma *vs* 97.1 per cent non-Roma; OR 1.58) and fewer Roma possess medical insurance (81.6 per cent Roma *vs* 89.1 per cent non-Roma; OR 1.86) than non-Roma ( Table 2).

Finally, we used the UN definition of extreme poverty (2USD/person/day or less) in addition to two other variables as an overall indicator of impoverished conditions ( Table 2). Roma report a slightly greater, though not significant, incidence of lacking working electricity in their homes or dwellings (13.2 per cent Roma *vs* 2.7 per cent non-Roma), as well as lacking piped gas and/or the ability to purchase gas tanks (32.7 per cent Roma *vs.* 18.9 per cent non-Roma, p=0.12) ( Table 2). Moreover, Roma report greater incidences of severe poverty (2USD/day/person or less) than non-Roma (55.1% per cent *vs.* 43.2 per cent) ( Table 2), although overall, those in rural areas are significantly more susceptible to extreme poverty than those in suburban or urban communities (61.8 per cent rural *vs.* 32.6 per cent urban) ( Table 3).

### Multivariate analyses

Following univariate analysis, we used general multivariate linear regression analysis for four distinct models, combining categories that indicated a specific score (WASH, WASH Safe, poverty, healthcare) or approached a level of significance in the univariate analysis ( Table 4). These analyses further demonstrate the significant (α = 0.05) disparity between Roma and non-Roma.

**Table 4.** Multivariate analysis modelling. Romania, 2016. All models use non-Roma as reference. * indicates significance at 95% CI level. ** indicates significance at 90% CI level.

MOD1	Regression coefficient	p-value	95% Confidence Interval
Property Documents	0.0854	0.279	0.069, 0.240
Education Level	0.2613 ^*^	0.001	0.100, 0.422
Household Size	0.2362 ^*^	0.002	0.083, 0.389
Employment Status	0.0505	0.559	-0.119, 0.220
MOD2	Regression coefficient	p-value	95% Confidence Interval
Improved Water Source	-0.1914 ^*^	0.05	-0.383, -0.0000465
Moderate/Severe Diarrhea	0.1302 ^**^	0.08	-0.016, 0.276
Electricity in Dwelling	0.1802	0.139	-0.058, 0.419
Insecure Housing	0.2860 ^*^	0.001	0.111, 0.461
MOD3	Regression coefficient	p-value	95% Confidence Interval
WASH Score	-0.4104 ^*^	0.017	-0.747, -0.074
Healthcare Score	0.3407 ^**^	0.066	-0.022, 0.704
Poverty Score	0.3391 ^*^	0.013	0.070, 0.608
MOD4	Regression coefficient	p-value	95% Confidence Interval
WASH Safe Score	-0.250	0.203	-0.521, 0.111
Healthcare Score	0.3277 ^**^	0.083	-0.042, 0.698
Poverty Score	0.3305 ^*^	0.02	0.052, 0.609

A multivariate combination of demographic variables further highlights the difference in education level and household size between Roma and non-Roma. Roma households are significantly larger than non-Roma households, but whether this is a correlation with birth rate or the presence of multiple generations in a single dwelling is beyond the scope of this study. Furthermore, Roma individuals are far less likely to complete required education (10 ^th^ grade) than non-Roma individuals (MOD1; Table 4). In our univariate analysis, we broke down the score categories to their individual components and identified significant factors to further explore. Multivariate analysis of these parameters points to insecure housing as having the strongest correlation with being Roma, followed by access to tap water (improved water source), and less significantly, the occurrence of moderate or severe diarrhoea (MOD2; Table 4).

Finally, we analysed our four score categories, using two different approaches. We first analysed the WASH score, as defined by the DRI, together with the healthcare and poverty scores (MOD3; Table 4). Healthcare and poverty equally significantly correlate with being Roma. The WASH score, however, is negatively correlated to the Roma, indicating that Roma individuals actually have an advantage over non-Roma individuals. To further investigate this question, we ran an additional analysis with healthcare and poverty, but substituting our WASH Safe score (MOD4; Table 4). The significant difference observed in healthcare and poverty remains, but when protected well water is included alongside tap water in the definition of improved or safe water sources, the disparity associated with WASH is eliminated.

Dataset 1. Coded survey data http://dx.doi.org/10.5256/f1000research.12546.d177233
Romania, 2016. Excel file of compiled responses to survey questions. Coded and de-identified. Numerical code corresponds to responses as indicated on the study surveys ( Supplementary material 1 and Supplementary material 2). Click here for additional data file. Copyright: © 2018 Powell Doherty R et al. 2018 Data associated with the article are available under the terms of the Creative Commons Zero "No rights reserved" data waiver (CC0 1.0 Public domain dedication).

Dataset 2. Python Notebook data analysis and statistics http://dx.doi.org/10.5256/f1000research.12546.d177234
Romania, 2016. Python Notebook analysis of survey data. Click here for additional data file. Copyright: © 2018 Powell Doherty R et al. 2018 Data associated with the article are available under the terms of the Creative Commons Zero "No rights reserved" data waiver (CC0 1.0 Public domain dedication).

Dataset 3. Python Notebook data analysis and statistics http://dx.doi.org/10.5256/f1000research.12546.d177235
Romania, 2016. Python Notebook analysis of survey data, exported as a PDF file. Click here for additional data file. Copyright: © 2018 Powell Doherty R et al. 2018 Data associated with the article are available under the terms of the Creative Commons Zero "No rights reserved" data waiver (CC0 1.0 Public domain dedication).

### CART Analysis and *Hot Spot* Generation

Following the CART-based assignment of categories to the synthetic population, we used ArcGIS to determine, at the *judet* (county) level, which regions of Romania are most in need of development and/or government aid based on seven key parameters. We reduced each parameter to a binary distinction during the generation of the population in order to simplify the visualization process, and all parameters are presented on a continuous scale using standard deviation from the mean.

***Poverty Parameters*.** First, we visualized the availability of electricity to households throughout the country ( Figure 3.3A). Analysis of survey responses indicated the presence or absence of electricity in a household was a significant distinction between Roma and non-Roma families. Our visualization (darker regions indicate areas of greater risk and/or need) demonstrates that households most likely to lack electricity are clustered in the middle of the country where Brasov, Sibiu, and Mures counties meet, and extend into the North-West corner into Bihor, Salaj, and Satu-Mare counties. Additional areas at risk are observed along the southern border in Dolj county, as well as in select areas near the capitol, Bucharest.

**Figure 3.**
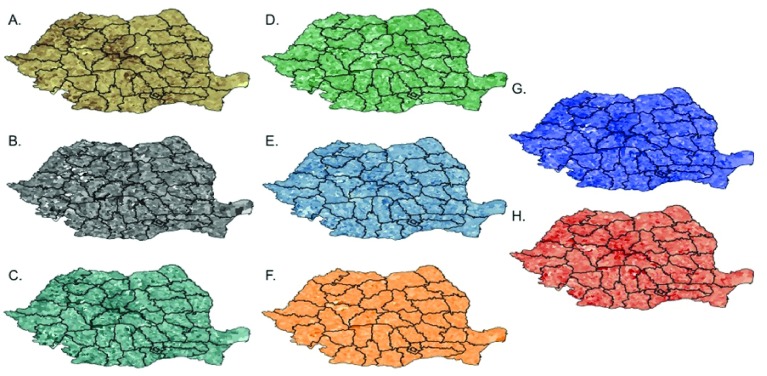
Visualization of quality of life parameters ***A**) Lack of Electricity, **B**) Severe poverty, **C**) Insecure Housing, **D**) Lack of 'improved water source, **E**) High incidence of diarrheal disease, **F**) Urban versus rural distribution, G) Prevalence of lack of education and H) Cumulative risk.* Following the assignment of categories to the synthetic population, we used ArcGIS to determine, at county level, what regions are most in need of development and/or government aid based on key parameters. We reduced each parameter to a binary distinction during the generation of the population, so as to simplify the visualization process, and all parameters are presented on a continuous scale using standard deviation from the mean.

**Figure 4.**
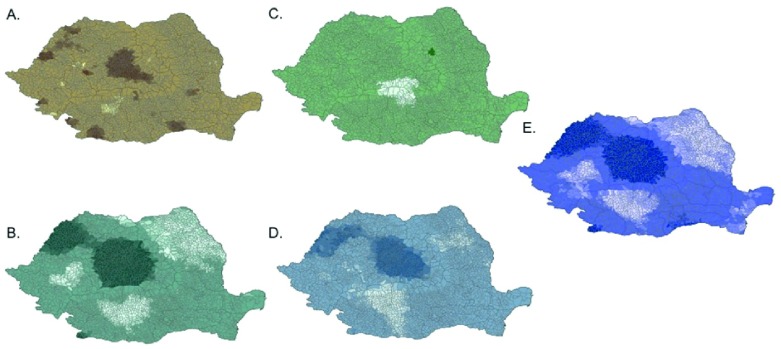
Hot spot analysis for quality of life parameters. ***A**) Lack of Electricity, **B**) Insecure Housing, **C**) Lack of 'improved water source', **D**) High incidence of diarrheal disease, and E) Lack of education beyond 8th grade.* Using spatial autocorrelation (Global Moran’s I), each variable was analyzed to determine whether the pattern expressed in the population was random. Significant autocorrelation (non-random pattern or clustering) was determined by z-score and accompanying p-value (p=0.05). Significance or lack thereof suggests whether the independent variables upon which our synthetic population was built (household size and ethnicity) are appropriate indicators for our specific QoL parameters. For variables demonstrated to be significantly spatially auto-correlated, we progressed to Incremental Spatial Autocorrelation with a fixed distance measure to identify areas of intense need (dark shading) or ‘hot spot’ clustering.

**Figure 5.**
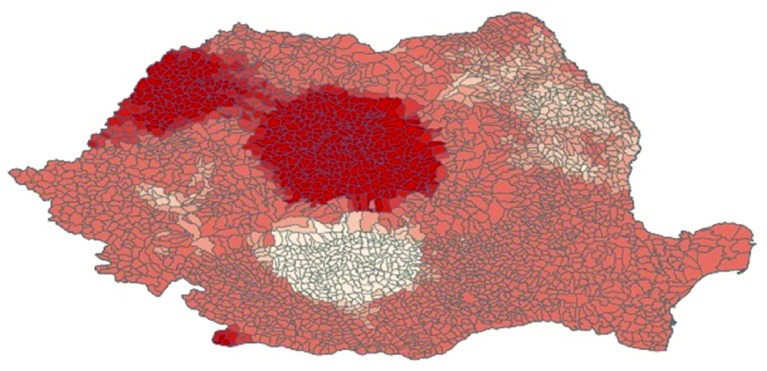
Hot Spot Analysis for Cumulative Risk. Hot spot analysis visualization of cumulative need across the country. Including all significantly auto-correlated parameters, with the addition of the measure of severe poverty, need of *any kind* is significantly auto-correlated in Romania (z-score = 11.5, p-value < 0.0001) and most apparent in the central portion of the country and extending to the North-West corner (dark red). These areas correlate with locations of Roma communities.

We then visualized the level of severe poverty, defined as the inability to spend more than U.S. $2 per person in a household per day ( Figure 3.3B). Households with the greatest number of individuals at risk or currently experiencing severe poverty, shown by dark regions on the map, are in the middle portion of the country where Brasov, Sibiu, and Mures counties meet. Additional regions of risk include Caras-Severin county in the far West and various smaller pockets along the Eastern border counties.

***Healthcare and Infrastructure*.** Analysis of insecure housing, rates of diarrheal disease, and the lack of access to an ‘improved water source’ (as defined by the WHO, ^15^) led us to explore these issues in our population as parameters indicative of health status and deficiencies in essential infrastructure ( Figure 3.3C–E). In general, these three parameters mimic the same patterns demonstrated by our poverty parameters, with a concentration of areas of need in the Central region and North-West corner of the country. Specifically, areas with prominent levels of insecure housing are where Brasov, Sibiu, and Mures counties meet in the middle portion of the country, along with regions in the North-West counties of Bihor, Salaj, Cluj, and Bistrita-Nasaud ( Figure 3.3C). Additional smaller pockets in the South-West include areas of Caras-Severin, Mehedinti, and Dolj counties.

The lack of access to an ‘improved water source’, in contrast to insecure housing, is not a widespread issue, nor is it concentrated in one particular region, with only small pockets of affected areas spread throughout the country ( Figure 3.3D). The areas with the highest rates of diarrheal disease cluster in Sibiu and Mures counties (excepting Brasov), as well as in Bihor, Salaj, Bistrita-Nasaud, and Satu-Mare. Additionally, clusters of high diarrheal disease rates are observed in the more southern county of Arges. Isolated clusters are also identified along the South-Western and North-Eastern border counties ( Figure 3.3E).

***Education and Geographical Classification*.** To provide a geographical classification to frame our other variables of interest, we visualized all of Romania to identify urban *versus* rural areas. The most urban areas are lightest (such as the capital of Bucharest), while the most rural areas are darker shades and predominantly align with the Carpathian mountain range and the boundary of the country with the Black Sea ( Figure 3.3F).

We next visualized areas of the country in which portions of the population have not completed beyond an eighth-grade education or are at risk for not doing so ( Figure 3.3G). The regions most in need of aid in this area are again Mures and Sibiu counties in the central portion of the country, Bihor and Satu-Mare counties in the North-West, and Arges, Giurgiu, and Dambovita counties in the Southern portion of the country. In addition, we observe some at risk areas in and around the city limits of the capital, Bucharest.

***Cumulative Risk*.** Upon visualization of each individual QoL variable, we generated a map to indicate cumulative need across all variables ( Figure 3.3H). Unsurprisingly, areas of greatest cumulative need mimic the patterns identified in individual variables and are concentrated in the central portion of the country in Brasov, Sibiu, and Mures counties. Additional regions include areas of Arges and Dambovita counties, along with isolated clusters throughout the country.

***Parameter Correlation and Hot Spot Analysis*.** We used the spatial autocorrelation Global Moran’s I together with the Incremental Spatial Autocorrelation test to both validate our model and determine whether the pattern of clustering for each variable was significant. This analysis provides additional information beyond initial ArcGIS visualization ( Figure 3.3), as it allows analysis down to the commune level and highlights the distinct patterns exhibited by the various parameters. Variables that did not demonstrate significance using Global Moran’s, including geographical classification and severe poverty, were not carried through to hot spot visualization.

Analysis of the lack of electricity variable demonstrated that this variable is significantly geographically auto-correlated (z-score = 24.802, p-value<0.0001) and aligns with prior visualization, showing intense hotspots of need concentrated predominantly in the central portion of the country (dark areas on the map). In addition, hot spots are observed just south of Bucharest, and in select communes in the South-West and North-West portions of the country ( Figure 3.4A).

Likewise, analysis of the variable indicating areas with a strong prevalence of insecure housing was also significantly clustered by geographical region (z-score = 15.46, p-value<0.0001). It too aligns with previous visualization, as well as with *some* areas that are in need of access to electricity ( Figure 3.4B). However, comparing the two variables, there are also communes that exhibit a need for better housing that are, paradoxically, not deficient in access to electricity, particularly in the North-West corner of the country.

Analysis of the ‘improved water source’ metric demonstrated that, while significant (z-score = 2.179, p-value = 0.029), the pattern of clustering is not as strong as in other variables, highlighting only one small hot spot throughout the country ( Figure 3.4C).

Autocorrelation analysis of rates of diarrheal disease indicated significant geographical clustering (z-score = 8.548, p-value < 0.0001) and also appeared to be most concentrated in the central and North-West judets ( Figure 3.4D). Analyzing more deeply, we observe numerous communes that appear in both the electricity variable and the housing variable. Alternatively, communes in Bihor and Satu-Mare counties in the North-West corner demonstrate particularly high rates of diarrheal disease and insecure housing, but not a significant lack of electricity.

The education variable ( Figure 3.4E), indicating communes and/or judets with a significant number of individuals at risk for or already failing to progress beyond eighth grade, demonstrates a clustering pattern most similar to the insecure housing variable ( Figure 3.4B). Significantly auto-correlated (z-score = 18.499, p-value < 0.0001), hot spots are most intense in the central and North-West counties. Much like the prevalence of insecure housing and, to a lesser extent, the lack of electricity, hot spots also appear in the Southern regions of the country, outside Bucharest and throughout Dolj county. The clustering pattern observed in these three parameters is distinctly different from that which appears in diarrheal disease and water quality analysis.

Finally, we utilized our analysis to visualize hot spots of cumulative need across the country ( Figure 3.5). Including all significantly auto-correlated parameters, with the addition of the measure of severe poverty, need of *any kind* is significantly auto-correlated in Romania (z-score = 11.5, p-value < 0.0001) and, not surprisingly, most apparent in the central portion of the country and extending to the North-West corner.

## Discussion

A number of studies have examined the various factors *the Decade of Roma Inclusion* (DRI) sought to address in Roma communities across the EU, both during the implementation of the project and since its conclusion in 2015 ^5, 10, 12, 24, 25^. Unfortunately, while some improvements did occur, a number of studies indicate the DRI did not achieve its stated goals in the areas of education, housing, employment, and health status of Roma in participating countries ^26, 27^. Our study supports these conclusions, particularly with regard to education, healthcare, and poverty. However, disparities that other studies have highlighted in multiple countries with regard to employment and sanitation do not necessarily occur in Romania ^25, 28– 30^. Rather, both the Roma and non-Roma in rural Romania face similar challenges regarding access to full-time employment and water, which are exacerbated by a lack of municipal sanitation services in over 800 Romanian communities ^31^. The lack of significant difference between Roma and non-Roma in our sample in relation to indoor toilets and bathrooms does not indicate that either ethnic group has an advantage, but rather all those who reside in rural communities face a disadvantage, regardless of ethnicity. Notably, our findings indicate that, in some instances, the Roma appear to have a slight advantage over non-Roma ( Table 4). Using the DRI definition of piped water to an indoor or outdoor tap, our analyses indicate Romanian and other non-Roma individuals lag behind the Roma in ‘improved water sources’. However, when one accounts for the prevalence of private, protected wells (WASH Safe score), the disparity is minimized and no longer significant ( Table 4). We postulate this distinction is indicative of how our survey collected this type of data, and future iterations will refine how we classify ‘safe’ and ‘improved’ water sources.

Of additional interest is the key indicator that those in suburban and urban areas, Roma and non-Roma alike, take longer to reach their chosen primary drinking water sources than do their rural counterparts. However, this statistic is potentially ambiguous. The urban community included in this study reported overwhelmingly that it had recently been subject to a contamination of the municipal water supply with coliform bacteria and, thus, the majority of residents therein reported the need to purchase water rather than use the taps available in their homes. It was not possible to collect data regarding the behaviour of these residents prior to the contamination event. Furthermore, the suburban community included here recently experienced the loss of a bridge, connecting the far side of the river to the village centre on the other side. Those individuals stranded on the far side of the bridge (predominantly Roma) reported numerous problems with their wells, requiring them to travel 5km or more to the nearest crossing to reach a shop or market until the bridge is restored. Therefore, this statistic is potentially a reflection of the walking or driving time that would otherwise be unnecessary.

Despite the evidence presented that Roma and non-Roma alike are subjected to ineffective sanitation and hygiene services throughout the country, one should note that the Roma population still reports a greater incidence of diarrheal disease *and* a reduced rate of immunization than the non-Roma population. There are potentially a number of reasons for this. Unlike in other countries ^5, 30^, the Romanian Roma report fairly equivalent rates of medical insurance and access to primary care, but the type of treatment received when care is sought was beyond the scope of this study and may be a contributing factor. Indeed, Roma individuals have elsewhere reported poor health related to both their unhygienic circumstances and the care they receive ^25, 32, 33^. In addition, as has already been noted, both literacy rates and overall levels of education are significantly decreased in the Romanian Roma population. This is in contrast to education rates in Roma populations of other countries, as the educational component of the DRI has been lauded as the most successful portion of the initiative, albeit only for primary school attendance ^26, 27^. Rates of disease and healthcare status overall are inversely associated with education ^34^, which may offer another possible explanation for the disparity in diarrheal disease rates. It is important to consider, however anecdotally, the Roma do report some knowledge of personal water treatment and safety (data not shown), through the use of salt or lime in personal wells and a commitment to boiling water before drinking or cooking if possible. However, the lack of infrastructure and services works against these individual and imperfect efforts. Furthermore, for those Roma who do have access to tap water (municipal or otherwise), many of them report using an alternative primary water source. While these same individuals indicate that they believe their tap water to be safe (data not shown), their daily activities are in direct contrast to this assertion.

While the population data are of interest, our primary focus is using that data to demonstrate the utility of our CART analysis and *hot spot* generation tool. Recognizing the limited nature of our population size and to corroborate the validity of our approach, we searched for areas in Romania with development issues that were previously identified using more traditional methods. In particular, the areas identified as having a high prevalence of individuals experiencing insecure housing, lack of electricity and diarrheal disease ( Figures 3.3 and 3.4) align with areas known for informal settlements, populated predominantly by Roma families, found in the suburban and urban areas surrounding the North-Western city of Cluj and the far North-West town of Baia Mare ^35^. These areas extend westward into Bihor and Salaj counties, as well as southward into Mures county, the sites predicted to be the most concentrated hot spots on our maps. Our methodology also identifies incorporated areas (villages, cities, etc.) that suffer from specific issues. For example, the village of Holbav and numerous others in Brasov county have been highlighted as areas with energy poor communities with little indication of infrastructure improvements on the horizon ^36^. These villages are in the central region of Romania and fall in the most intense hot spot for lack of electricity, as predicted by our model. Furthermore, in a case study by Vincze, the city of *Calafat* in Dolj county was characterized following the demise of its manufacturing economy ^37^. The study highlighted the particular problems facing the Roma community in that area, noting a lack of formal employment along with inadequate housing and precarious government services. This portion of Dolj county is highlighted as a hot spot for housing, education, and generalized need in our model. These areas coincide with those identified in our model as regions of intense need across multiple variables and also boast large concentrations of Roma. Thus, our model provides corroborating evidence to demonstrate how the Roma minority in Romania are consistently at risk in key quality of life indicators and frequently lack access to basic services. However, as indicated by our survey data, non-Roma within these areas are likely also at risk.

Interestingly, our model only produces a small hot spot in the Eastern portion of Romania as an area of need related to ‘improved water source’ access. At first glance, this suggests that the WASH infrastructure in the country is better than initially anticipated. However, while there are no true hot spots, there are also no ‘cold’ spots. These results indicate that limited access to clean, reliable water sources is a ubiquitous problem across the country and *not* confined to specific geographic regions aligning with the Roma minority.

This model and subsequent analyses serve as an example of the utility of synthetic populations and how their use in conjunction with traditional surveys, time-use data, and census data can augment the conclusions generated from those kinds of data. Using a model such as ours, conclusions of greater complexity can be made. While it is possible to analyze survey data for information, that analysis is severely restricted to the area in which the survey was conducted and the questions that were asked. Furthermore, the analysis only achieves a summary view of the population. In contrast, merging survey data with population statistics and conducting analyses via the synthetic population allows one to identify geographical regions with similar characteristics and populations with key identifiable traits, and combine the two to extrapolate conclusions beyond the original survey regions. This approach requires fewer on-the-ground resources and allows conclusions to be visualized in an accessible fashion for use in project proposals and grant justifications.

### Limitations and future directions

The primary limitation of this study is the use of a small sample (n = 135) of survey respondents to generate the categories necessary for CART analysis and random household assignment. Constraints of limited time, funding and personnel, which are often factors in community-based public health studies, inhibited our ability to interact with more than 30 households per community and restricted the study to five communities. Future iterations will seek to obtain a more robust survey sample size for integration into the synthetic population. While acknowledging this limitation, we do note our ability to validate the predictions of the existing model via identification of similar conclusions from more traditional methods, thereby suggesting that the methodology is sound. Thus, using this type of model, conclusions can be drawn and applied to a larger population and geographic area even with limited resources and sample size, providing a valid methodology to conduct similar studies to highlight hot spots of need. Similar methodologies could also be applied to geographic areas with restricted access due to geography or political unrest, which limits the ability to assess needs within these areas. Additionally, subsequent studies can use these and other data to generate detailed models that explore specific initiatives that could be implemented to address discrepancies in equality and access, and progress the literature around Roma health disparities beyond analysis and into intervention testing.

## Conclusions

The model and approach demonstrated herein provides a useful tool to identify and predict both areas of need and the type of need required in a given region. Furthermore, this approach allows populations to be separated based on ethnicity and other characteristics, and to determine if subpopulations require different kinds of assistance compared to the majority. Therefore, we assert this approach can and should be utilized by non-profit organizations, NGOs, and government funding agencies to more appropriately focus valuable time and resources during project planning and development to ensure aid reaches those who are in greatest need.

## Data availability

The data referenced by this article are under copyright with the following copyright statement: Copyright: © 2018 Powell Doherty R et al.

Data associated with the article are available under the terms of the Creative Commons Zero "No rights reserved" data waiver (CC0 1.0 Public domain dedication). 

**Dataset 1: Coded survey data**. Romania, 2016. Excel file of compiled responses to survey questions. Coded and de-identified. Numerical code corresponds to responses as indicated on the study surveys ( Supplementary material 1 and Supplementary material 2).

DOI, 10.5256/f1000research.12546.d177233 ^38^

**Dataset 2: Python Notebook data analysis and statistics.** Romania, 2016. Python Notebook analysis of survey data.

DOI, 10.5256/f1000research.12546.d177234 ^39^

**Dataset 3: Python Notebook data analysis and statistics.** Romania, 2016. Python Notebook analysis of survey data, exported as a PDF file.

DOI, 10.5256/f1000research.12546.d177235 ^40^

## Supplementary material

**Supplementary material 1: Quality of Life Survey, English.** Romania 2016. Survey questions provided in English.

**Supplementary material 2: Quality of Life Survey, Romanian.** Romania, 2016. Survey questions provided in Romanian.

## References

[1] European Investment Bank: EIB loan signatures in Romania amounted to EUR 211 million in 2015.2015.

[2] United Nations Development Programme: Millennium Development Goals in Romania.2007.

[3] European Union: European parliament resolution of 31 January 2008 on a European strategy on the Roma.2008 Reference Source

[4] World Bank: Roma.2015 Reference Source

[5] Cook B Wayne GF Valentine A: Revisiting the evidence on health and health care disparities among the Roma: a systematic review 2003-2012. *Int J Public Health.* 2013;58(6):885–911. 10.1007/s00038-013-0518-6 24096986

[6] Hajioff S McKee M: The health of the Roma people: a review of the published literature. *J Epidemiol Community Health.* 2000;54(11):864–9. 10.1136/jech.54.11.864 11027202 PMC1731574

[7] Zeman CL Depken DE Senchina DS: Roma health issues: a review of the literature and discussion. *Ethn Health.* 2003;8(3):223–49. 10.1080/1355785032000136434 14577997

[8] Parekh N Rose T: Health inequalities of the Roma in Europe: a literature review. *Cent Eur J Public Health.* 2011;19(3):139–42. 10.21101/cejph.a3661 22026288

[9] Gladdis K: Life inside the Romanian gypsy ghetto that is so grim the town mayor sealed it off behind a wall. *Daily Mail* 2013 Reference Source

[10] Friedman E: Decade of Roma Inclusion Progress Report 2005-2013.2014 Reference Source

[11] Flecha A: Healthier lives for European minority groups: school and health care, lessons from the Roma. *Int J Environ Res Public Health.* 2013;10(8):3089–111. 10.3390/ijerph10083089 23887619 PMC3774426

[12] Fésüs G Östlin P McKee M: Policies to improve the health and well-being of Roma people: the European experience. *Health Policy.* 2012;105(1):25–32. 10.1016/j.healthpol.2011.12.003 22217864

[13] Gould R: Roma rights and Roma expulsions in France: Official discourse and EU responses. *Critical Social Policy.* 2015;35(1):24–44. 10.1177/0261018314545595

[14] Dale CR: Development Planning: Concepts and Tools for Planners, Managers and Facilitators. London: Zed Books;2004 Reference Source

[15] World Health Organization: Core Questions on Drinking-Water and Sanitation for Household Surveys.2006 Reference Source

[16] Digital Raster Data-Romania [Internet]. Oak Ridge National Laboratory.2013 Reference Source

[17] International Data Base-Romania [Internet].2016 Reference Source

[18] Lester A: Global Population NDSSL Tech Report.2016.

[19] ESRI: ArcGIS Desktop: Release 10. Redlands, CA: Environmental Systems Research Institute;2011.

[20] Hijmans R: GADM database of Global Administrative Areas.2015 Reference Source

[21] McKinney W, editor: Data Structures for Statistical Computing in Python. *9th Python in Science Conference*; SciPy.2010 Reference Source

[22] NIS Romania: Populaţia stabilă după etnie – judeţe, municipii, oraşe, comune. In: Romania INdS, editor.2011.

[23] Rivers C: Modeling Emerging Infectious Diseases for Public Health Decision Support. Blacksburg, VA: Virginia Polytechnic Institute and State University;2015 Reference Source

[24] Curcic SM Maj A Shayna P: Inclusion, Integration or Perpetual Exclusion? A Critical Examination of the Decade of Roma Inclusion, 2005-2015. *Educ Res Eval J.* 2014;13(3):257–67. 10.2304/eerj.2014.13.3.257

[25] Masseria C Mladovsky P Hernández-Quevedo C: The socio-economic determinants of the health status of Roma in comparison with non-Roma in Bulgaria, Hungary and Romania. *Eur J Public Health.* 2010;20(5):549–54. 10.1093/eurpub/ckq102 20650945

[26] Bojadjieva A: Roma Inclusion Index. Budapest, Hungary: Decade of Roma Inclusion Secretariat Foundation;2015 Reference Source

[27] Jovanovic Z: Why Europe’s “Roma Decade” Didn’t Lead to Inclusion. In: Foundations OS, editor.2015 Reference Source

[28] Molnár A Adány R Adám B: Health impact assessment and evaluation of a Roma housing project in Hungary. *Health Place.* 2010;16(6):1240–7. 10.1016/j.healthplace.2010.08.011 20801071

[29] Janevic T Jankovic J Bradley E: Socioeconomic position, gender, and inequalities in self-rated health between Roma and non-Roma in Serbia. *Int J Public Health.* 2012;57(1):49–55. 10.1007/s00038-011-0277-1 21814849

[30] Kühlbrandt C Footman K Rechel B: An examination of Roma health insurance status in Central and Eastern Europe. *Eur J Public Health.* 2014;24(5):707–12. 10.1093/eurpub/cku004 24500807

[31] Duminica G: Raport Anual, 2015. Bucharest, Romania: Agentia Împreunã;2015.

[32] Casals M Pila P Langohr K: Incidence of infectious diseases and survival among the Roma population: a longitudinal cohort study. *Eur J Public Health.* 2012;22(2):262–6. 10.1093/eurpub/ckq204 21217119

[33] Teira R Suárez-Lozano I Lozano F: Characteristics and outcome of HIV infection in gypsies in the Spanish VACH Cohort. *Enferm Infecc Microbiol Clin.* 2010;28(5):266–72. 10.1016/j.eimc.2009.04.018 20129716

[34] Regidor E De Mateo S Calle ME: Educational level and mortality from infectious diseases. *J Epidemiol Community Health.* 2002;56(9):682–3. 10.1136/jech.56.9.682 12177084 PMC1732238

[35] Small Steps PROJECT: Romania-Cluj-Napoca Translyvania.2017 Reference Source

[36] Gaedtke F: In Transylvania, energy poverty persists in the developed world.2016 Reference Source

[37] Vincze E: Precarization of Working Class Roma through Spatial Deprivation, Labor Destitution and Racialization. *Review of Sociology.* 2015;25(4):58–85. Reference Source

[38] Powell Doherty R Telionis PA Müller-Demary D: Dataset 1 in: A survey of quality of life indicators in the Romanian Roma population following the ‘Decade of Roma Inclusion’. *F1000Research.* 2018;6:1692 10.5256/f1000research.12546.d177233 PMC6357989 30774929

[39] Powell Doherty R Telionis PA Müller-Demary D: Dataset 2 in: A survey of quality of life indicators in the Romanian Roma population following the ‘Decade of Roma Inclusion’. *F1000Research.* 2018;6:1692 10.5256/f1000research.12546.d177234 PMC6357989 30774929

[40] Powell Doherty R Telionis PA Müller-Demary D: Dataset 3 in: A survey of quality of life indicators in the Romanian Roma population following the ‘Decade of Roma Inclusion’. *F1000Research.* 2018;6:1692 10.5256/f1000research.12546.d177235 PMC6357989 30774929

