# Supplementary material for: A survey of quality of life indicators in the Romanian Roma population following the ‘Decade of Roma Inclusion’
Source: F1000Res. 2018 Dec 13;6:1692. Originally published 2017 Sep 15. [Version 3] doi: 10.12688/f1000research.12546.3 (PMC6357989; doi:10.12688/f1000research.12546.3)
Supplement: Dataset 3. Python Notebook data analysis and statistics — http://dx.doi.org/10.5256/f1000research.12546.d177235 Romania, 2016. Python Notebook analysis of survey data, exported as a PDF file. [file f1000research-6-19000-s0002.tgz › 657f32a1-76f3-4fdc-a24b-2bf029bc2172_Romania_Demographics.pdf]

Romania_Demographics

September 1, 2017

In [1]:
```
import pandas as pd
import epipy
import seaborn as sns
%pylab inline

import statsmodels.api as sm
from scipy import stats
import numpy as np
```

```
/Users/RebeccaD/Library/Enthought/Canopy_64bit/User/lib/python2.7/site-packages/matplotlib/font_
  warnings.warn('Matplotlib is building the font cache using fc-list. This may take a moment.')
```

```
Populating the interactive namespace from numpy and matplotlib
```

In [2]:
```
my_data = pd.read_csv('surveys_allcommunities_transposed.csv')
```

In [3]:
```
pd.__version__
```

Out[3]:
```
u'0.18.0'
```

In [4]:
```
my_data.Age.describe()
```

Out[4]:
```
count    135.000000
mean      46.874074
std       16.457739
min       17.000000
25%       35.500000
50%       45.000000
75%       60.000000
max       91.000000
Name: Age, dtype: float64
```

In [8]:
```
Ethnicity = my_data.Ethnicity.value_counts(normalize=True)
```

In [11]:
```
Ethnicity = my_data.Ethnicity.value_counts()
```

In [12]:
```
Ethnicity
```

```
Out[12]: 1    81
         2    35
         3    17
         4     2
         Name: Ethnicity, dtype: int64
```

```
In [13]: Gender = my_data.Gender.value_counts()
```

```
In [14]: Gender
```

```
Out[14]: 2    68
         1    67
         Name: Gender, dtype: int64
```

```
In [15]: Education = my_data.Education.value_counts()
```

```
In [17]: my_data[['Gender', 'Education']][my_data.Ethnicity == 1].describe()
```

Out[17]:

	Gender	Education
count	81.000000	81.000000
mean	1.419753	4.320988
std	0.496593	2.301234
min	1.000000	1.000000
25%	1.000000	3.000000
50%	1.000000	4.000000
75%	2.000000	5.000000
max	2.000000	10.000000

```
In [18]: my_data[['Gender', 'Education']][my_data.Ethnicity == 3].describe()
```

Out[18]:

	Gender	Education
count	17.000000	17.000000
mean	1.823529	3.470588
std	0.392953	2.452490
min	1.000000	1.000000
25%	2.000000	2.000000
50%	2.000000	3.000000
75%	2.000000	5.000000
max	2.000000	9.000000

```
In [19]: my_data[['Gender', 'Education']][my_data.Ethnicity == 2].describe()
```

Out[19]:

	Gender	Education
count	35.000000	35.000000
mean	1.542857	6.142857
std	0.505433	2.390457
min	1.000000	2.000000
25%	1.000000	5.000000
50%	2.000000	6.000000
75%	2.000000	8.000000
max	2.000000	9.000000

```
In [20]: my_data[['Gender', 'Education']][my_data.Ethnicity == 4].describe()
```

Out[20]:

	Gender	Education
count	2.000000	2.00000
mean	1.500000	4.50000
std	0.707107	2.12132
min	1.000000	3.00000
25%	1.250000	3.75000
50%	1.500000	4.50000
75%	1.750000	5.25000
max	2.000000	6.00000

```
In [23]: my_data.Ethnicity.replace(3, 1)
```

```
Out[23]: 0      1
         1      1
         2      1
         3      1
         4      1
         5      1
         6      1
         7      1
         8      1
         9      1
         10     1
         11     1
         12     1
         13     1
         14     1
         15     1
         16     1
         17     1
         18     1
         19     1
         20     1
         21     1
         22     1
         23     1
         24     1
         25     1
         26     2
         27     1
         28     1
         29     1
               ..
         105    1
         106    1
         107    1
```

```
108    1
109    1
110    2
111    2
112    2
113    1
114    2
115    2
116    2
117    2
118    4
119    1
120    1
121    1
122    1
123    1
124    1
125    2
126    1
127    1
128    1
129    1
130    1
131    1
132    1
133    1
134    1
Name: Ethnicity, dtype: int64
```

In [24]:
```
my_data.Ethnicity.replace(4, 2)& my_data.Ethnicity.replace(3, 1)
```

Out[24]:
```
0      1
1      1
2      1
3      1
4      1
5      1
6      1
7      1
8      1
9      1
10     1
11     1
12     1
13     1
14     1
15     1
16     1
```

```
17     1
18     1
19     1
20     1
21     1
22     1
23     1
24     1
25     1
26     2
27     1
28     1
29     1
      ..
105    1
106    1
107    1
108    1
109    1
110    2
111    2
112    2
113    1
114    2
115    2
116    2
117    2
118    0
119    1
120    1
121    1
122    1
123    1
124    1
125    2
126    1
127    1
128    1
129    1
130    1
131    1
132    1
133    1
134    1
Name: Ethnicity, dtype: int64
```

In [25]:
```
my_data['Roma'] = 0
my_data.loc[(my_data.Ethnicity ==2)|(my_data.Ethnicity ==4),'Roma'] = 1
```

```
In [26]: my_data['Roma'].head()

Out[26]: 0      0
         1      0
         2      0
         3      0
         4      0
         5      0
         6      0
         7      0
         8      0
         9      0
         10     0
         11     0
         12     0
         13     0
         14     0
         15     0
         16     0
         17     0
         18     0
         19     0
         20     0
         21     0
         22     0
         23     0
         24     0
         25     0
         26     1
         27     0
         28     0
         29     0
               ..
         105    0
         106    0
         107    0
         108    0
         109    0
         110    1
         111    1
         112    1
         113    0
         114    1
         115    1
         116    1
         117    1
         118    1
         119    0
```

```
120    0
121    0
122    0
123    0
124    0
125    1
126    0
127    0
128    0
129    0
130    0
131    0
132    0
133    0
134    0
Name: Roma, dtype: int64
```

In [27]: `my_data`

Out[27]:

```
     Survey  Community  Household size  Time lived there  Head of house  \
0         1          1               8              51.0              1
1         2          1               4              15.0              1
2         3          1               4               4.0              1
3         4          1               1              11.0              1
4         5          1               9              12.0              1
5         6          1              12              40.0              2
6         7          1               7              35.0              2
7         8          1               4               4.0              1
8         9          1               4               7.0              1
9        10          1               1              30.0              2
10       11          1               2              33.0              1
11       12          1               2              45.0              1
12       13          1               8              12.0              2
13       14          1               3              25.0              1
14       15          1               6              30.0              2
15       16          1               8              32.0              2
16       17          1               1              50.0              2
17       18          1               6              60.0              1
18       19          1               5              30.0              2
19       20          1               2              50.0              2
20       21          1               7              40.0              1
21       22          1               3              10.0              1
22       23          1               2              35.0              1
23       24          1               8              35.0              1
24       25          1               1              40.0              2
25       26          1               7              26.0              3
26       27          1               5              40.0              1
27       28          1               3              37.0              2
```

```
28     29      1           4        30.0           2
29     30      2          10        15.0           1
..    ...    ...         ...         ...         ...
105   106      4           4        20.0           2
106   107      4           6        40.0           1
107   108      4           9        41.0           1
108   109      4           8        18.0           1
109   110      4           4        18.0           2
110   111      4           6         8.0           2
111   112      4           3         NaN           1
112   113      4           1        30.0           2
113   114      4           5        10.0           1
114   115      4           4        22.0           2
115   116      4           1        55.0           2
116   117      4           5        52.0           2
117   118      4           2        70.0           2
118   119      4           1        45.0           2
119   120      5           5        40.0           1
120   121      5           4         3.0           1
121   122      5           3        23.0           1
122   123      5           7        40.0           1
123   124      5           4        14.0           1
124   125      5           9        20.0           1
125   126      5           4        13.0           1
126   127      5           2        15.0           1
127   128      5           9        10.0           1
128   129      5           3        30.0           1
129   130      5           4        20.0           1
130   131      5           3        45.0           1
131   132      5           5        45.0           1
132   133      5           4        50.0           1
133   134      5           5        30.0           1
134   135      5           4        12.0           1

     50+ men  50+ women  15-49 men  15-49 women  under 15 boys  ...   \
0          1          1          2            2              1  ...
1          0          0          2            2              0  ...
2          0          0          1            1              0  ...
3          1          0          0            0              0  ...
4          1          1          2            2              1  ...
5          1          1          1            4              2  ...
6          0          1          3            1              2  ...
7          0          0          1            1              0  ...
8          0          0          1            1              1  ...
9          0          1          0            0              0  ...
10         1          1          0            0              0  ...
11         1          1          0            0              0  ...
12         1          1          1            1              2  ...
```

13	0	0	1	2	0	...
14	0	1	2	2	0	...
15	0	1	1	2	1	...
16	0	1	0	0	0	...
17	1	1	2	2	0	...
18	1	1	1	0	2	...
19	0	1	1	0	0	...
20	1	1	1	1	3	...
21	0	0	1	1	0	...
22	1	0	0	0	1	...
23	0	1	2	2	1	...
24	0	1	0	0	0	...
25	1	0	2	2	1	...
26	1	0	3	1	0	...
27	1	1	1	0	0	...
28	0	1	1	1	1	...
29	1	1	3	3	1	...
..	...	...	...	...	...	...
105	0	1	2	0	1	...
106	0	0	3	2	0	...
107	1	1	3	3	1	...
108	0	0	1	4	1	...
109	0	0	2	1	0	...
110	0	0	2	1	3	...
111	0	0	1	1	0	...
112	0	1	0	0	0	...
113	0	0	1	2	2	...
114	0	1	0	1	2	...
115	0	1	0	0	0	...
116	1	1	1	2	0	...
117	0	2	0	0	0	...
118	0	1	0	0	0	...
119	0	1	1	3	0	...
120	0	0	1	1	1	...
121	0	0	1	2	0	...
122	1	1	4	1	0	...
123	1	0	1	1	1	...
124	0	0	5	3	1	...
125	0	0	2	1	0	...
126	0	0	1	1	0	...
127	0	0	2	2	4	...
128	0	1	1	1	0	...
129	0	0	2	1	1	...
130	0	0	1	2	0	...
131	0	1	3	1	0	...
132	1	1	1	1	0	...
133	0	0	2	2	0	...
134	0	0	2	1	0	...

	Piped__tank_gas	Decider for female earned money \
0	1	NaN
1	0	NaN
2	0	NaN
3	0	NaN
4	0	NaN
5	0	NaN
6	0	NaN
7	0	NaN
8	0	NaN
9	0	NaN
10	0	NaN
11	1	NaN
12	0	NaN
13	1	NaN
14	1	NaN
15	1	NaN
16	1	NaN
17	1	NaN
18	1	NaN
19	0	NaN
20	1	NaN
21	1	NaN
22	0	NaN
23	1	NaN
24	1	NaN
25	1	NaN
26	1	NaN
27	1	NaN
28	1	NaN
29	1	NaN
..	...	...
105	0	NaN
106	0	NaN
107	0	NaN
108	0	NaN
109	1	NaN
110	0	NaN
111	0	NaN
112	1	NaN
113	1	NaN
114	1	NaN
115	1	NaN
116	0	NaN
117	1	NaN
118	1	NaN
119	1	NaN

```
120                 1                              NaN
121                 1                              NaN
122                 1                              NaN
123                 1                              NaN
124                 1                              NaN
125                 1                              NaN
126                 1                              NaN
127                 0                              NaN
128                 0                              NaN
129                 1                              NaN
130                 1                              NaN
131                 0                              NaN
132                 0                              NaN
133                 1                              NaN
134                 1                              NaN

     Conflict between Roma and non  Roma segregated  Community helpful  House  \
0                              NaN              NaN                NaN      1
1                              NaN              NaN                NaN      1
2                              NaN              NaN                NaN      1
3                              NaN              NaN                NaN      0
4                              NaN              NaN                NaN      1
5                              NaN              NaN                NaN      0
6                              NaN              NaN                NaN      1
7                              NaN              NaN                NaN      1
8                              NaN              NaN                NaN      1
9                              NaN              NaN                NaN      1
10                             NaN              NaN                NaN      1
11                             NaN              NaN                NaN      1
12                             NaN              NaN                NaN      1
13                             NaN              NaN                NaN      0
14                             NaN              NaN                NaN      1
15                             NaN              NaN                NaN      1
16                             NaN              NaN                NaN      1
17                             NaN              NaN                NaN      0
18                             NaN              NaN                NaN      1
19                             NaN              NaN                NaN      1
20                             NaN              NaN                NaN      1
21                             NaN              NaN                NaN      1
22                             NaN              NaN                NaN      1
23                             NaN              NaN                NaN      1
24                             NaN              NaN                NaN      1
25                             NaN              NaN                NaN      1
26                             NaN              NaN                NaN      1
27                             NaN              NaN                NaN      0
28                             NaN              NaN                NaN      1
29                             NaN              NaN                NaN      1
..                             ...              ...                ...    ...
```

105	NaN	NaN	NaN	1
106	NaN	NaN	NaN	1
107	NaN	NaN	NaN	1
108	NaN	NaN	NaN	1
109	NaN	NaN	NaN	1
110	NaN	NaN	NaN	1
111	NaN	NaN	NaN	0
112	NaN	NaN	NaN	1
113	NaN	NaN	NaN	1
114	NaN	NaN	NaN	1
115	NaN	NaN	NaN	1
116	NaN	NaN	NaN	1
117	NaN	NaN	NaN	1
118	NaN	NaN	NaN	1
119	NaN	NaN	NaN	1
120	NaN	NaN	NaN	1
121	NaN	NaN	NaN	1
122	NaN	NaN	NaN	1
123	NaN	NaN	NaN	1
124	NaN	NaN	NaN	0
125	NaN	NaN	NaN	1
126	NaN	NaN	NaN	0
127	NaN	NaN	NaN	1
128	NaN	NaN	NaN	1
129	NaN	NaN	NaN	1
130	NaN	NaN	NaN	1
131	NaN	NaN	NaN	1
132	NaN	NaN	NaN	1
133	NaN	NaN	NaN	0
134	NaN	NaN	NaN	1

	Roof	Floor	Housing_Score	Roma
0	0	1	2	0
1	0	1	2	0
2	0	1	2	0
3	0	0	0	0
4	0	1	2	0
5	1	0	1	0
6	0	1	2	0
7	0	1	2	0
8	1	1	3	0
9	1	0	2	0
10	1	1	3	0
11	1	1	3	0
12	0	1	2	0
13	0	0	0	0
14	0	1	2	0
15	0	1	2	0

16	1	0	2	0
17	0	1	1	0
18	0	0	1	0
19	1	0	2	0
20	0	1	2	0
21	0	1	2	0
22	0	0	1	0
23	0	0	1	0
24	1	0	2	0
25	0	0	1	0
26	0	1	2	1
27	0	1	1	0
28	0	1	2	0
29	0	1	2	0
..	...	...	...	...
105	0	0	1	0
106	0	1	2	0
107	1	1	3	0
108	0	1	2	0
109	1	1	3	0
110	1	1	3	1
111	1	1	2	1
112	1	1	3	1
113	1	1	3	0
114	1	1	3	1
115	1	1	3	1
116	1	1	3	1
117	1	0	2	1
118	1	1	3	1
119	1	1	3	0
120	1	1	3	0
121	1	1	3	0
122	1	0	2	0
123	0	1	2	0
124	0	0	0	0
125	0	1	2	1
126	0	0	0	0
127	0	1	2	0
128	0	1	2	0
129	0	1	2	0
130	1	1	3	0
131	1	1	3	0
132	0	1	2	0
133	1	1	2	0
134	0	1	2	0

[135 rows x 61 columns]

```
In [28]: my_data[['Education', 'Roma']]
```

Out[28]:

	Education	Roma
0	8	0
1	8	0
2	6	0
3	4	0
4	7	0
5	5	0
6	3	0
7	4	0
8	3	0
9	2	0
10	5	0
11	2	0
12	4	0
13	9	0
14	5	0
15	1	0
16	1	0
17	3	0
18	2	0
19	1	0
20	2	0
21	3	0
22	4	0
23	3	0
24	3	0
25	6	0
26	8	1
27	5	0
28	1	0
29	4	0
..	...	...
105	1	0
106	4	0
107	2	0
108	3	0
109	10	0
110	8	1
111	2	1
112	2	1
113	3	0
114	8	1
115	2	1
116	3	1
117	3	1
118	3	1

```
119          9     0
120          6     0
121          9     0
122          5     0
123          4     0
124          5     0
125          5     1
126          6     0
127          5     0
128          4     0
129          4     0
130          9     0
131          9     0
132         10     0
133          4     0
134          3     0

[135 rows x 2 columns]
```

In [31]:
```
my_data['Education_level'] = 0
my_data.loc[(my_data.Education <6),'Education_level'] = 1 #5 indicates secondary school
```

In [32]:
```
my_data['Education_level'].head()
```

Out[32]:
```
0      0
1      0
2      0
3      1
4      0
5      1
6      1
7      1
8      1
9      1
10     1
11     1
12     1
13     0
14     1
15     1
16     1
17     1
18     1
19     1
20     1
21     1
22     1
23     1
```

```
24     1
25     0
26     0
27     1
28     1
29     1
      ..
105    1
106    1
107    1
108    1
109    0
110    0
111    1
112    1
113    1
114    0
115    1
116    1
117    1
118    1
119    0
120    0
121    0
122    1
123    1
124    1
125    1
126    0
127    1
128    1
129    1
130    0
131    0
132    0
133    1
134    1
Name: Education_level, dtype: int64
```

In [33]: my_data

```
Out[33]:     Survey  Community  Household size  Time lived there  Head of house  \
         0        1          1               8              51.0              1
         1        2          1               4              15.0              1
         2        3          1               4               4.0              1
         3        4          1               1              11.0              1
         4        5          1               9              12.0              1
         5        6          1              12              40.0              2
```

6	7	1	7	35.0	2
7	8	1	4	4.0	1
8	9	1	4	7.0	1
9	10	1	1	30.0	2
10	11	1	2	33.0	1
11	12	1	2	45.0	1
12	13	1	8	12.0	2
13	14	1	3	25.0	1
14	15	1	6	30.0	2
15	16	1	8	32.0	2
16	17	1	1	50.0	2
17	18	1	6	60.0	1
18	19	1	5	30.0	2
19	20	1	2	50.0	2
20	21	1	7	40.0	1
21	22	1	3	10.0	1
22	23	1	2	35.0	1
23	24	1	8	35.0	1
24	25	1	1	40.0	2
25	26	1	7	26.0	3
26	27	1	5	40.0	1
27	28	1	3	37.0	2
28	29	1	4	30.0	2
29	30	2	10	15.0	1
..	...	...	...	...	...
105	106	4	4	20.0	2
106	107	4	6	40.0	1
107	108	4	9	41.0	1
108	109	4	8	18.0	1
109	110	4	4	18.0	2
110	111	4	6	8.0	2
111	112	4	3	NaN	1
112	113	4	1	30.0	2
113	114	4	5	10.0	1
114	115	4	4	22.0	2
115	116	4	1	55.0	2
116	117	4	5	52.0	2
117	118	4	2	70.0	2
118	119	4	1	45.0	2
119	120	5	5	40.0	1
120	121	5	4	3.0	1
121	122	5	3	23.0	1
122	123	5	7	40.0	1
123	124	5	4	14.0	1
124	125	5	9	20.0	1
125	126	5	4	13.0	1
126	127	5	2	15.0	1
127	128	5	9	10.0	1

128	129	5	3	30.0	1
129	130	5	4	20.0	1
130	131	5	3	45.0	1
131	132	5	5	45.0	1
132	133	5	4	50.0	1
133	134	5	5	30.0	1
134	135	5	4	12.0	1

	50+ men	50+ women	15-49 men	15-49 women	under 15 boys	\
0	1	1	2	2	1	
1	0	0	2	2	0	
2	0	0	1	1	0	
3	1	0	0	0	0	
4	1	1	2	2	1	
5	1	1	1	4	2	
6	0	1	3	1	2	
7	0	0	1	1	0	
8	0	0	1	1	1	
9	0	1	0	0	0	
10	1	1	0	0	0	
11	1	1	0	0	0	
12	1	1	1	1	2	
13	0	0	1	2	0	
14	0	1	2	2	0	
15	0	1	1	2	1	
16	0	1	0	0	0	
17	1	1	2	2	0	
18	1	1	1	0	2	
19	0	1	1	0	0	
20	1	1	1	1	3	
21	0	0	1	1	0	
22	1	0	0	0	1	
23	0	1	2	2	1	
24	0	1	0	0	0	
25	1	0	2	2	1	
26	1	0	3	1	0	
27	1	1	1	0	0	
28	0	1	1	1	1	
29	1	1	3	3	1	
..	...	...	...	...	...	
105	0	1	2	0	1	
106	0	0	3	2	0	
107	1	1	3	3	1	
108	0	0	1	4	1	
109	0	0	2	1	0	
110	0	0	2	1	3	
111	0	0	1	1	0	
112	0	1	0	0	0	

113	0	0	1	2	2
114	0	1	0	1	2
115	0	1	0	0	0
116	1	1	1	2	0
117	0	2	0	0	0
118	0	1	0	0	0
119	0	1	1	3	0
120	0	0	1	1	1
121	0	0	1	2	0
122	1	1	4	1	0
123	1	0	1	1	1
124	0	0	5	3	1
125	0	0	2	1	0
126	0	0	1	1	0
127	0	0	2	2	4
128	0	1	1	1	0
129	0	0	2	1	1
130	0	0	1	2	0
131	0	1	3	1	0
132	1	1	1	1	0
133	0	0	2	2	0
134	0	0	2	1	0

	...	Decider for female earned money \
0	...	NaN
1	...	NaN
2	...	NaN
3	...	NaN
4	...	NaN
5	...	NaN
6	...	NaN
7	...	NaN
8	...	NaN
9	...	NaN
10	...	NaN
11	...	NaN
12	...	NaN
13	...	NaN
14	...	NaN
15	...	NaN
16	...	NaN
17	...	NaN
18	...	NaN
19	...	NaN
20	...	NaN
21	...	NaN
22	...	NaN
23	...	NaN

```
24         ...                                    NaN
25         ...                                    NaN
26         ...                                    NaN
27         ...                                    NaN
28         ...                                    NaN
29         ...                                    NaN
..         ...                                    ...
105        ...                                    NaN
106        ...                                    NaN
107        ...                                    NaN
108        ...                                    NaN
109        ...                                    NaN
110        ...                                    NaN
111        ...                                    NaN
112        ...                                    NaN
113        ...                                    NaN
114        ...                                    NaN
115        ...                                    NaN
116        ...                                    NaN
117        ...                                    NaN
118        ...                                    NaN
119        ...                                    NaN
120        ...                                    NaN
121        ...                                    NaN
122        ...                                    NaN
123        ...                                    NaN
124        ...                                    NaN
125        ...                                    NaN
126        ...                                    NaN
127        ...                                    NaN
128        ...                                    NaN
129        ...                                    NaN
130        ...                                    NaN
131        ...                                    NaN
132        ...                                    NaN
133        ...                                    NaN
134        ...                                    NaN

     Conflict between Roma and non  Roma segregated  Community helpful  House  \
0                              NaN              NaN                NaN      1
1                              NaN              NaN                NaN      1
2                              NaN              NaN                NaN      1
3                              NaN              NaN                NaN      0
4                              NaN              NaN                NaN      1
5                              NaN              NaN                NaN      0
6                              NaN              NaN                NaN      1
7                              NaN              NaN                NaN      1
8                              NaN              NaN                NaN      1
```

9	NaN	NaN	NaN	1
10	NaN	NaN	NaN	1
11	NaN	NaN	NaN	1
12	NaN	NaN	NaN	1
13	NaN	NaN	NaN	0
14	NaN	NaN	NaN	1
15	NaN	NaN	NaN	1
16	NaN	NaN	NaN	1
17	NaN	NaN	NaN	0
18	NaN	NaN	NaN	1
19	NaN	NaN	NaN	1
20	NaN	NaN	NaN	1
21	NaN	NaN	NaN	1
22	NaN	NaN	NaN	1
23	NaN	NaN	NaN	1
24	NaN	NaN	NaN	1
25	NaN	NaN	NaN	1
26	NaN	NaN	NaN	1
27	NaN	NaN	NaN	0
28	NaN	NaN	NaN	1
29	NaN	NaN	NaN	1
..	...	...	...	...
105	NaN	NaN	NaN	1
106	NaN	NaN	NaN	1
107	NaN	NaN	NaN	1
108	NaN	NaN	NaN	1
109	NaN	NaN	NaN	1
110	NaN	NaN	NaN	1
111	NaN	NaN	NaN	0
112	NaN	NaN	NaN	1
113	NaN	NaN	NaN	1
114	NaN	NaN	NaN	1
115	NaN	NaN	NaN	1
116	NaN	NaN	NaN	1
117	NaN	NaN	NaN	1
118	NaN	NaN	NaN	1
119	NaN	NaN	NaN	1
120	NaN	NaN	NaN	1
121	NaN	NaN	NaN	1
122	NaN	NaN	NaN	1
123	NaN	NaN	NaN	1
124	NaN	NaN	NaN	0
125	NaN	NaN	NaN	1
126	NaN	NaN	NaN	0
127	NaN	NaN	NaN	1
128	NaN	NaN	NaN	1
129	NaN	NaN	NaN	1
130	NaN	NaN	NaN	1

131	NaN	NaN	NaN	1
132	NaN	NaN	NaN	1
133	NaN	NaN	NaN	0
134	NaN	NaN	NaN	1

	Roof	Floor	Housing_Score	Roma	Education_level
0	0	1	2	0	0
1	0	1	2	0	0
2	0	1	2	0	0
3	0	0	0	0	1
4	0	1	2	0	0
5	1	0	1	0	1
6	0	1	2	0	1
7	0	1	2	0	1
8	1	1	3	0	1
9	1	0	2	0	1
10	1	1	3	0	1
11	1	1	3	0	1
12	0	1	2	0	1
13	0	0	0	0	0
14	0	1	2	0	1
15	0	1	2	0	1
16	1	0	2	0	1
17	0	1	1	0	1
18	0	0	1	0	1
19	1	0	2	0	1
20	0	1	2	0	1
21	0	1	2	0	1
22	0	0	1	0	1
23	0	0	1	0	1
24	1	0	2	0	1
25	0	0	1	0	0
26	0	1	2	1	0
27	0	1	1	0	1
28	0	1	2	0	1
29	0	1	2	0	1
..	...	...	...	...	...
105	0	0	1	0	1
106	0	1	2	0	1
107	1	1	3	0	1
108	0	1	2	0	1
109	1	1	3	0	0
110	1	1	3	1	0
111	1	1	2	1	1
112	1	1	3	1	1
113	1	1	3	0	1
114	1	1	3	1	0
115	1	1	3	1	1

```
116     1      1              3     1                1
117     1      0              2     1                1
118     1      1              3     1                1
119     1      1              3     0                0
120     1      1              3     0                0
121     1      1              3     0                0
122     1      0              2     0                1
123     0      1              2     0                1
124     0      0              0     0                1
125     0      1              2     1                1
126     0      0              0     0                0
127     0      1              2     0                1
128     0      1              2     0                1
129     0      1              2     0                1
130     1      1              3     0                0
131     1      1              3     0                0
132     0      1              2     0                0
133     1      1              2     0                1
134     0      1              2     0                1

[135 rows x 62 columns]
```

```
In [34]: table = epipy.create_2x2(my_data, row='Roma', column='Education_level', row_order=[1,0]
                          col_order=[0, 1])

In [35]: table #row: non Roma = 1, Roma = 0; Columns: equal to or less than 8th grade = 1, great
```

```
Out[35]:       0   1  All
         1    20  17   37
         0    23  75   98
         All  43  92  135
```

```
In [36]: epipy.analyze_2x2(table)

Odds ratio: 3.84 (95% CI: (1.73, 8.52))
Relative risk: 2.3 (95% CI: (1.45, 3.67))

Chi square: 11.574780877
p value: 0.0208099530432


In [37]: table_2 = epipy.create_2x2(my_data, row='Gender', column='Education_level', row_order=[
                          col_order=[0, 1]) #row: 1=M, 2=F; column: 0= greater than 8th grade, 1

In [38]: table_2
```

```
Out[38]:       0   1  All
         1    26  41   67
         2    17  51   68
         All  43  92  135
```

```
In [39]: epipy.analyze_2x2(table_2)
```

```
Odds ratio: 1.9 (95% CI: (0.91, 3.97))
Relative risk: 1.55 (95% CI: (0.93, 2.58))

Chi square: 2.96343264718
p value: 0.563963392325
```

0.1 Community Breakdown for each variable

```
In [40]: my_data.groupby('Community').Age.mean()

Out[40]: Community
         1    49.413793
         2    45.833333
         3    45.600000
         4    52.700000
         5    35.687500
         Name: Age, dtype: float64

In [41]: my_data.groupby('Community').Ethnicity.describe()

Out[41]: Community
         1          count    29.000000
                    mean      2.206897
                    std       0.977581
                    min       1.000000
                    25%       1.000000
                    50%       3.000000
                    75%       3.000000
                    max       3.000000
         2          count    30.000000
                    mean      1.200000
                    std       0.406838
                    min       1.000000
                    25%       1.000000
                    50%       1.000000
                    75%       1.000000
                    max       2.000000
         3          count    30.000000
                    mean      1.400000
                    std       0.498273
                    min       1.000000
                    25%       1.000000
                    50%       1.000000
                    75%       2.000000
                    max       2.000000
         4          count    30.000000
```

```
                mean      1.700000
                std       0.794377
                min       1.000000
                25%       1.000000
                50%       2.000000
                75%       2.000000
                max       4.000000
5               count    16.000000
                mean      1.062500
                std       0.250000
                min       1.000000
                25%       1.000000
                50%       1.000000
                75%       1.000000
                max       2.000000
dtype: float64
```

```
In [42]: my_data.groupby('Community').Ethnicity.value_counts()
```

```
Out[42]: Community  Ethnicity
         1          3            17
                    1            11
                    2             1
         2          1            24
                    2             6
         3          1            18
                    2            12
         4          2            15
                    1            13
                    4             2
         5          1            15
                    2             1
         dtype: int64
```

```
In [43]: my_data.groupby('Community')['Household size'].mean()
```

```
Out[43]: Community
         1    4.724138
         2    5.666667
         3    4.866667
         4    4.300000
         5    4.687500
         Name: Household size, dtype: float64
```

```
In [44]: my_data['Family size']=0
         my_data.loc[(my_data['Household size'] >3),'Family size'] = 1
```

```
In [45]: table_3 = epipy.create_2x2(my_data, row='Roma', column='Family size', row_order=[1,0],
                                  col_order=[0, 1]) #row: non Roma = 1, Roma = 0; #column avg size = 0,
```

```
In [46]: table_3
```

```
Out[46]:       0   1  All
         1    21  16   37
         0    26  72   98
         All  47  88  135
```

```
In [47]: epipy.analyze_2x2(table_3)
```

```
Odds ratio: 3.63 (95% CI: (1.65, 8.01))
Relative risk: 2.14 (95% CI: (1.39, 3.3))

Chi square: 10.8130085638
p value: 0.0287479012745
```

```
In [48]: my_data.Education.describe()
```

```
Out[48]: count    135.000000
         mean       4.688889
         std        2.487181
         min        1.000000
         25%        3.000000
         50%        4.000000
         75%        6.000000
         max       10.000000
         Name: Education, dtype: float64
```

```
In [49]: my_data[['Education']][my_data.Roma == 0].describe()
```

```
Out[49]:        Education
         count  98.000000
         mean    4.173469
         std     2.337615
         min     1.000000
         25%     2.000000
         50%     4.000000
         75%     5.000000
         max    10.000000
```

```
In [50]: my_data[['Education']][my_data.Roma == 1].describe()
```

```
Out[50]:        Education
         count  37.000000
         mean    6.054054
         std     2.379845
         min     2.000000
         25%     5.000000
         50%     6.000000
         75%     8.000000
         max     9.000000
```

```
In [51]: my_data[['Age']][my_data.Community==2].hist()

Out[51]: array([[<matplotlib.axes._subplots.AxesSubplot object at 0x115ca41d0>]], dtype=object)
```

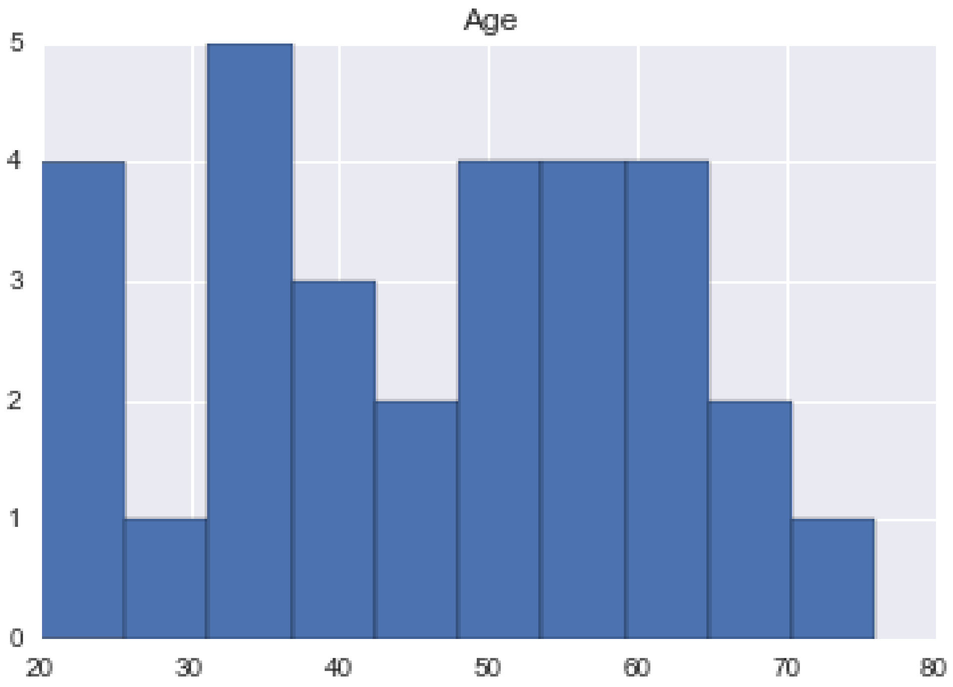


```
In [52]: my_data[['Age']][my_data.Community==5].describe()

Out[52]:             Age
         count  16.000000
         mean   35.687500
         std    10.473578
         min    19.000000
         25%    27.750000
         50%    39.000000
         75%    43.500000
         max    53.000000

In [53]: my_data[['Household size']][my_data.Roma == 0].describe()

Out[53]:        Household size
         count       98.000000
         mean         5.295918
         std          2.561448
         min          1.000000
         25%          3.000000
         50%          5.000000
         75%          7.000000
         max         12.000000
```

In [54]:
```
my_data[['Household size']][my_data.Roma == 1].describe()
```

Out[54]:
```
       Household size
count       37.000000
mean         3.729730
std          2.022768
min          1.000000
25%          2.000000
50%          3.000000
75%          5.000000
max          9.000000
```

In [55]:
```
my_data['Household size'].describe()
```

Out[55]:
```
count    135.000000
mean       4.866667
std        2.517995
min        1.000000
25%        3.000000
50%        5.000000
75%        6.000000
max       12.000000
Name: Household size, dtype: float64
```

In [57]:
```
my_data['Marital status'].describe()
```

Out[57]:
```
count    135.000000
mean       2.311111
std        1.672725
min        1.000000
25%        1.000000
50%        1.000000
75%        3.000000
max        6.000000
Name: Marital status, dtype: float64
```

In [58]:
```
my_data['Marital status'].value_counts()
```

Out[58]:
```
1    73
3    29
5    14
6     9
2     6
4     4
Name: Marital status, dtype: int64
```

In [59]:
```
my_data['Marital status simple']=0
my_data.loc[(my_data['Marital status'] ==4),'Marital status simple'] = 1
```

In [60]:
```
my_data.loc[(my_data['Marital status'] ==5),'Marital status simple'] = 1
```

```
In [61]: my_data.loc[(my_data['Marital status'] ==6),'Marital status simple'] = 1

In [62]: my_data[my_data.Roma == 0]['Marital status simple'].value_counts(normalize=True)

Out[62]: 0    0.816327
         1    0.183673
         Name: Marital status simple, dtype: float64

In [63]: my_data[my_data.Roma == 1]['Marital status simple'].value_counts(normalize=True) #Marit

Out[63]: 0    0.756757
         1    0.243243
         Name: Marital status simple, dtype: float64

In [64]: my_data.Occupation.value_counts()

Out[64]: 4    57
         1    38
         3    21
         2    15
         6     2
         5     2
         Name: Occupation, dtype: int64

In [65]: my_data[my_data.Roma == 0]['Occupation'].value_counts()

Out[65]: 4    42
         1    26
         2    14
         3    12
         6     2
         5     2
         Name: Occupation, dtype: int64

In [66]: my_data[my_data.Roma == 1]['Occupation'].value_counts(normalize=True)

Out[66]: 4    0.405405
         1    0.324324
         3    0.243243
         2    0.027027
         Name: Occupation, dtype: float64

In [67]: my_data['Employment status']=0 #0 is FT employment, 1 is day labor or unemployed (inc r

In [68]: my_data.loc[(my_data['Occupation'] !=1),'Employment status'] = 1

In [69]: my_data[my_data.Roma==0]['Employment status'].value_counts(normalize=True)

Out[69]: 1    0.734694
         0    0.265306
         Name: Employment status, dtype: float64
```

```
In [70]: my_data[my_data.Roma==1]['Employment status'].value_counts(normalize=True)

Out[70]: 1    0.675676
         0    0.324324
         Name: Employment status, dtype: float64

In [71]: table_4 = epipy.create_2x2(my_data, row='Roma', column='Employment status', row_order=[
                          col_order=[0, 1]) #row: non Roma = 1, Roma = 0; #column FT employment

In [72]: table_4

Out[72]:       0   1  All
         1    12  25   37
         0    26  72   98
         All  38  97  135

In [73]: epipy.analyze_2x2(table_4)

Odds ratio: 1.33 (95% CI: (0.58, 3.02))
Relative risk: 1.22 (95% CI: (0.69, 2.16))

Chi square: 0.462570768361
p value: 0.97704093156


In [74]: my_data['Total men']= my_data['50+ men'] + my_data['15-49 men']

In [75]: my_data['Total women'] = my_data['50+ women'] + my_data['15-49 women']

In [76]: my_data[['Total men', 'literate men (+15)']]

Out[76]:      Total men  literate men (+15)
         0            3                 3.0
         1            2                 0.0
         2            1                 1.0
         3            1                 0.0
         4            3                 3.0
         5            2                 2.0
         6            3                 3.0
         7            1                 1.0
         8            1                 1.0
         9            0                 0.0
         10           1                 1.0
         11           1                 1.0
         12           2                 0.0
         13           1                 1.0
         14           2                 2.0
         15           1                 0.0
         16           0                 0.0
```

```
17           3                 2.0
18           2                 0.0
19           1                 0.0
20           2                 2.0
21           1                 1.0
22           1                 0.0
23           2                 2.0
24           0                 0.0
25           3                 1.0
26           4                 3.0
27           2                 0.0
28           1                 0.0
29           4                 NaN
..         ...               ...
105          2                 0.0
106          3                 3.0
107          4                 3.0
108          1                 1.0
109          2                 2.0
110          2                 2.0
111          1                 1.0
112          0                 0.0
113          1                 1.0
114          0                 0.0
115          0                 0.0
116          2                 2.0
117          0                 0.0
118          0                 0.0
119          1                 1.0
120          1                 1.0
121          1                 1.0
122          5                 5.0
123          2                 2.0
124          5                 5.0
125          2                 2.0
126          1                 1.0
127          2                 1.0
128          1                 1.0
129          2                 2.0
130          1                 1.0
131          3                 3.0
132          2                 2.0
133          2                 2.0
134          2                 2.0

[135 rows x 2 columns]
```

In [77]:
```
my_data['illiterate men']= my_data['Total men'] - my_data['literate men (+15)']
```

```
In [78]: my_data['illiterate men']

Out[78]: 0      0.0
         1      2.0
         2      0.0
         3      1.0
         4      0.0
         5      0.0
         6      0.0
         7      0.0
         8      0.0
         9      0.0
         10     0.0
         11     0.0
         12     2.0
         13     0.0
         14     0.0
         15     1.0
         16     0.0
         17     1.0
         18     2.0
         19     1.0
         20     0.0
         21     0.0
         22     1.0
         23     0.0
         24     0.0
         25     2.0
         26     1.0
         27     2.0
         28     1.0
         29     NaN
               ...
         105    2.0
         106    0.0
         107    1.0
         108    0.0
         109    0.0
         110    0.0
         111    0.0
         112    0.0
         113    0.0
         114    0.0
         115    0.0
         116    0.0
         117    0.0
         118    0.0
         119    0.0
```

```
120    0.0
121    0.0
122    0.0
123    0.0
124    0.0
125    0.0
126    0.0
127    1.0
128    0.0
129    0.0
130    0.0
131    0.0
132    0.0
133    0.0
134    0.0
Name: illiterate men, dtype: float64
```

```
In [79]: my_data[['Total women', 'literate women (+15)']]
```

Out[79]:

	Total women	literate women (+15)
0	3	3.0
1	2	0.0
2	1	1.0
3	0	0.0
4	3	3.0
5	5	5.0
6	2	1.0
7	1	0.0
8	1	1.0
9	1	0.0
10	1	1.0
11	1	1.0
12	2	2.0
13	2	2.0
14	3	2.0
15	3	1.0
16	1	1.0
17	3	2.0
18	1	0.0
19	1	0.0
20	2	2.0
21	1	1.0
22	0	0.0
23	3	0.0
24	1	1.0
25	2	2.0
26	1	1.0
27	1	0.0

```
28             2                   0.0
29             4                   NaN
..           ...                   ...
105            1                   0.0
106            2                   2.0
107            4                   3.0
108            4                   4.0
109            1                   1.0
110            1                   0.0
111            1                   1.0
112            1                   1.0
113            2                   1.0
114            2                   2.0
115            1                   1.0
116            3                   3.0
117            2                   2.0
118            1                   1.0
119            4                   4.0
120            1                   1.0
121            2                   2.0
122            2                   2.0
123            1                   1.0
124            3                   3.0
125            1                   1.0
126            1                   1.0
127            2                   1.0
128            2                   2.0
129            1                   1.0
130            2                   1.0
131            2                   2.0
132            2                   2.0
133            2                   2.0
134            1                   1.0

[135 rows x 2 columns]
```

```
In [80]: my_data['illiterate women']= my_data['Total women'] - my_data['literate women (+15)']
```

```
In [81]: my_data['illiterate women']
```

```
Out[81]: 0      0.0
         1      2.0
         2      0.0
         3      0.0
         4      0.0
         5      0.0
         6      1.0
         7      1.0
```

```
8       0.0
9       1.0
10      0.0
11      0.0
12      0.0
13      0.0
14      1.0
15      2.0
16      0.0
17      1.0
18      1.0
19      1.0
20      0.0
21      0.0
22      0.0
23      3.0
24      0.0
25      0.0
26      0.0
27      1.0
28      2.0
29      NaN
       ...
105     1.0
106     0.0
107     1.0
108     0.0
109     0.0
110     1.0
111     0.0
112     0.0
113     1.0
114     0.0
115     0.0
116     0.0
117     0.0
118     0.0
119     0.0
120     0.0
121     0.0
122     0.0
123     0.0
124     0.0
125     0.0
126     0.0
127     1.0
128     0.0
129     0.0
```

```
130    1.0
131    0.0
132    0.0
133    0.0
134    0.0
Name: illiterate women, dtype: float64
```

In [82]:
```
my_data['illiterate women'].value_counts()
```

Out[82]:
```
0.0    85
1.0    32
2.0    10
3.0     5
4.0     1
Name: illiterate women, dtype: int64
```

In [83]:
```
my_data['illiterate men'].value_counts()
```

Out[83]:
```
0.0    90
1.0    28
2.0    15
3.0     1
Name: illiterate men, dtype: int64
```

In [84]:
```
my_data['Total men'].value_counts()
```

Out[84]:
```
1    55
2    52
3    13
0     8
4     5
5     2
Name: Total men, dtype: int64
```

In [85]:
```
my_data['Total women'].value_counts()
```

Out[85]:
```
1    59
2    40
3    21
4     9
0     4
5     2
Name: Total women, dtype: int64
```

In [86]:
```
my_data['Total women'].sum()
```

Out[86]:
```
248
```

In [87]:
```
my_data['illiterate women'].sum()
```

Out[87]: 71.0

In [88]: my_data['Total men'].sum()

Out[88]: 228

In [89]: my_data['illiterate men'].sum()

Out[89]: 61.0

In [90]: my_data[my_data.Roma==0]['illiterate women'].sum()

Out[90]: 70.0

In [91]: my_data[my_data.Roma==1]['illiterate women'].sum()

Out[91]: 1.0

In [92]: my_data[my_data.Roma==0]['Total women'].sum()

Out[92]: 192

In [93]: my_data[my_data.Roma==1]['Total women'].sum()

Out[93]: 56

In [94]: my_data[my_data.Roma==0]['Total men'].sum()

Out[94]: 171

In [95]: my_data[my_data.Roma==0]['illiterate men'].sum()

Out[95]: 59.0

In [96]: my_data[my_data.Roma==1]['Total men'].sum()

Out[96]: 57

In [97]: my_data[my_data.Roma==1]['illiterate men'].sum()

Out[97]: 2.0

In [98]: *##How to run a ttest between illiterate Roma men and illiterate non-Roma men (repeat fo*

In [99]: my_data[my_data.Roma==0]['Property documents'].value_counts()

```
Out[99]: 1.0    61
         2.0    33
         Name: Property documents, dtype: int64
```

In [100]: my_data[my_data.Roma==1]['Property documents'].value_counts()

```
Out[100]: 1.0    26
          2.0     9
          Name: Property documents, dtype: int64

In [101]: table_5 = epipy.create_2x2(my_data, row='Roma', column='Property documents', row_order
                           col_order=[1, 2]) #rows: 1= nonRoma 0= Roma, columns, 1=yes 2=no

In [102]: table_5

Out[102]:        1   2  All
          1     26   9   37
          0     61  33   98
          All   87  42  135

In [103]: epipy.analyze_2x2(table_5)

Odds ratio: 1.56 (95% CI: (0.66, 3.72))
Relative risk: 1.14 (95% CI: (0.9, 1.46))

Chi square: 1.02377835553
p value: 0.906169234059


In [104]: my_data['weekly expenses (RON)'].describe()

Out[104]: count     127.000000
          mean      331.102362
          std       303.673222
          min         0.000000
          25%       120.000000
          50%       250.000000
          75%       500.000000
          max      2000.000000
          Name: weekly expenses (RON), dtype: float64

In [105]: my_data[my_data.Roma==0]['weekly expenses (RON)'].describe()

Out[105]: count      94.000000
          mean      332.712766
          std       321.598548
          min        35.000000
          25%       120.000000
          50%       250.000000
          75%       437.500000
          max      2000.000000
          Name: weekly expenses (RON), dtype: float64

In [108]: my_data[my_data.Roma==1]['weekly expenses (RON)'].describe()
```

```
Out[108]: count      33.000000
          mean      326.515152
          std       249.992140
          min         0.000000
          25%       150.000000
          50%       300.000000
          75%       500.000000
          max      1000.000000
          Name: weekly expenses (RON), dtype: float64
```

```
In [109]: my_data[['Roma', 'weekly expenses (RON)']]
```

```
Out[109]:      Roma  weekly expenses (RON)
          0       0                  500.0
          1       0                  250.0
          2       0                  125.0
          3       0                   35.0
          4       0                   40.0
          5       0                  170.0
          6       0                  500.0
          7       0                  200.0
          8       0                  180.0
          9       0                   50.0
          10      0                  170.0
          11      0                   50.0
          12      0                  140.0
          13      0                   70.0
          14      0                  300.0
          15      0                  150.0
          16      0                   80.0
          17      0                  100.0
          18      0                   40.0
          19      0                  100.0
          20      0                  200.0
          21      0                  100.0
          22      0                  200.0
          23      0                  400.0
          24      0                   50.0
          25      0                  500.0
          26      1                  500.0
          27      0                  100.0
          28      0                  150.0
          29      0                  100.0
          ..    ...                    ...
          105     0                 1000.0
          106     0                  375.0
          107     0                  100.0
          108     0                  225.0
```

```
109      0                      1300.0
110      1                        25.0
111      1                       100.0
112      1                      1000.0
113      0                       175.0
114      1                       500.0
115      1                       500.0
116      1                       300.0
117      1                       110.0
118      1                       200.0
119      0                       700.0
120      0                       100.0
121      0                         NaN
122      0                       700.0
123      0                       200.0
124      0                      1000.0
125      1                       200.0
126      0                       400.0
127      0                       300.0
128      0                      2000.0
129      0                       275.0
130      0                       500.0
131      0                      1000.0
132      0                       300.0
133      0                       500.0
134      0                         NaN

[135 rows x 2 columns]
```

```
In [110]: my_data.groupby('Community')['weekly expenses (RON)'].describe() #Can I compare ethnic
```

```
Out[110]: Community
          1          count      29.000000
                     mean      187.931034
                     std       151.413089
                     min        35.000000
                     25%        80.000000
                     50%       150.000000
                     75%       200.000000
                     max       500.000000
          2          count      29.000000
                     mean      267.241379
                     std       149.854609
                     min        60.000000
                     25%       150.000000
                     50%       250.000000
                     75%       350.000000
                     max       550.000000
```

```
3           count      26.000000
            mean      474.807692
            std       291.857776
            min       100.000000
            25%       300.000000
            50%       400.000000
            75%       575.000000
            max      1500.000000
4           count      29.000000
            mean      287.241379
            std       316.613177
            min         0.000000
            25%       100.000000
            50%       200.000000
            75%       350.000000
            max      1300.000000
5           count      14.000000
            mean      583.928571
            std       497.069019
            min       100.000000
            25%       281.250000
            50%       450.000000
            75%       700.000000
            max      2000.000000
dtype: float64
```

```
In [111]: my_data['WeeklyExpense_perPerson'] = my_data.apply(lambda x: (x['weekly expenses (RON)
```

```
In [112]: my_data['WeeklyExpense_perPerson']
```

```
Out[112]: 0        62.500000
          1        62.500000
          2        31.250000
          3        35.000000
          4         4.444444
          5        14.166667
          6        71.428571
          7        50.000000
          8        45.000000
          9        50.000000
          10       85.000000
          11       25.000000
          12       17.500000
          13       23.333333
          14       50.000000
          15       18.750000
          16       80.000000
          17       16.666667
```

```
18        8.000000
19       50.000000
20       28.571429
21       33.333333
22      100.000000
23       50.000000
24       50.000000
25       71.428571
26      100.000000
27       33.333333
28       37.500000
29       10.000000
           ...
105     250.000000
106      62.500000
107      11.111111
108      28.125000
109     325.000000
110       4.166667
111      33.333333
112    1000.000000
113      35.000000
114     125.000000
115     500.000000
116      60.000000
117      55.000000
118     200.000000
119     140.000000
120      25.000000
121            NaN
122     100.000000
123      50.000000
124     111.111111
125      50.000000
126     200.000000
127      33.333333
128     666.666667
129      68.750000
130     166.666667
131     200.000000
132      75.000000
133     100.000000
134            NaN
Name: WeeklyExpense_perPerson, dtype: float64
```

In [114]:
```
my_data['WeeklyExpense_perPerson'].value_counts()
```

Out[114]:
```
100.000000     15
50.000000      13
```

33.333333	7
75.000000	6
25.000000	5
16.666667	5
62.500000	3
83.333333	3
35.000000	3
71.428571	3
200.000000	3
166.666667	3
116.666667	2
66.666667	2
28.571429	2
41.666667	2
125.000000	2
250.000000	2
4.166667	2
58.333333	2
23.333333	1
10.000000	1
666.666667	1
40.000000	1
11.666667	1
1000.000000	1
500.000000	1
57.142857	1
20.000000	1
15.000000	1
	..
8.000000	1
28.125000	1
0.000000	1
7.500000	1
37.500000	1
18.750000	1
17.500000	1
31.250000	1
300.000000	1
14.285714	1
24.000000	1
55.000000	1
233.333333	1
140.000000	1
160.000000	1
110.000000	1
85.714286	1
22.222222	1
4.444444	1

```
90.000000       1
600.000000      1
14.166667       1
85.000000       1
111.111111      1
91.666667       1
11.111111       1
325.000000      1
60.000000       1
44.444444       1
80.000000       1
Name: WeeklyExpense_perPerson, dtype: int64
```

In [115]:
```
my_data[my_data.Roma==0]['WeeklyExpense_perPerson'].describe()
```

Out[115]:
```
count     94.000000
mean      74.320183
std       85.899155
min        4.166667
25%       25.781250
50%       50.000000
75%       90.178571
max      666.666667
Name: WeeklyExpense_perPerson, dtype: float64
```

In [116]:
```
my_data[my_data.Roma==1]['WeeklyExpense_perPerson'].describe()
```

Out[116]:
```
count      33.000000
mean      138.247956
std       200.811239
min         0.000000
25%        44.444444
50%        83.333333
75%       116.666667
max      1000.000000
Name: WeeklyExpense_perPerson, dtype: float64
```

In [117]:
```
my_data['Expense_person']=0
my_data.loc[(my_data['WeeklyExpense_perPerson']>56), 'Expense_person']=1 #56 RON is $1
```

In [118]:
```
stats.ttest_ind(my_data[my_data.Roma==1]['Expense_person'],my_data[my_data.Roma==0]['E
```

Out[118]:
```
Ttest_indResult(statistic=1.2277841809028889, pvalue=0.22169652389444819)
```

In [119]:
```
my_data[my_data.Roma==1]['Expense_person'].var()
```

Out[119]:
```
0.25225225225225234
```

In [120]:
```
my_data[my_data.Roma==0]['Expense_person'].var()
```

```
Out[120]: 0.24994740164106885

In [121]: table_6 = epipy.create_2x2(my_data, row='Roma', column='Expense_person', row_order=[0,
                                  col_order=[0, 1]) #rows: 1=nonRoma 0=Roma, columns 0= less than 56RON

In [122]: table_6

Out[122]:        0   1  All
          0     54  44   98
          1     16  21   37
          All   70  65  135

In [123]: epipy.analyze_2x2(table_6)

Odds ratio: 1.61 (95% CI: (0.75, 3.45))
Relative risk: 1.27 (95% CI: (0.85, 1.92))

Chi square: 1.51297406399
p value: 0.824340816635


In [124]: my_data['Primary drinking water source'].value_counts()

Out[124]: 7      48
          12     28
          11     16
          8      11
          9      10
          2       7
          1       7
          3       4
          6       3
          4       1
          Name: Primary drinking water source, dtype: int64

In [126]: my_data[my_data.Roma==0]['Primary drinking water source'].value_counts()

Out[126]: 7      29
          12     20
          11     16
          8       9
          9       6
          2       6
          3       4
          1       4
          6       3
          4       1
          Name: Primary drinking water source, dtype: int64

In [127]: my_data[my_data.Roma==1]['Primary drinking water source'].value_counts()
```

```
Out[127]: 7     19
          12     8
          9      4
          1      3
          8      2
          2      1
          Name: Primary drinking water source, dtype: int64
```

```
In [128]: my_data['tap water availabe']=0
```

```
In [129]: my_data.loc[(my_data['Primary drinking water source']!=1), 'tap water available']=0
          my_data.loc[(my_data['Primary drinking water source']!=11), 'tap water available']=0
          my_data.loc[(my_data['Primary drinking water source'] ==1),'tap water available'] = 1
          my_data.loc[(my_data['Primary drinking water source'] ==11),'tap water available'] = 1
```

```
In [130]: my_data[my_data.Roma==1]['tap water available'].value_counts() #92% of non Roma do NOT
```

```
Out[130]: 0.0    34
          1.0     3
          Name: tap water available, dtype: int64
```

```
In [131]: my_data[my_data.Roma==0]['tap water available'].value_counts() #80% of Roma do NOT hav
```

```
Out[131]: 0.0    78
          1.0    20
          Name: tap water available, dtype: int64
```

```
In [132]: table_7 = epipy.create_2x2(my_data, row='Roma', column='tap water available', row_orde
                                 col_order=[0, 1])
```

```
In [133]: table_7
```

```
Out[133]:        0   1  All
          0     78  20   98
          1     34   3   37
          All  112  23  135
```

```
In [134]: epipy.analyze_2x2(table_7)
```

```
Odds ratio: 0.34 (95% CI: (0.1, 1.24))
Relative risk: 0.87 (95% CI: (0.75, 0.99))

Chi square: 2.87494689835
p value: 0.578966061994
```

```
In [135]: my_data['safe water']=0  #safe water is defined as a personal use water source that is
```

```
In [136]: my_data.loc[(my_data['Primary drinking water source']!=1), 'safe water']=0
          my_data.loc[(my_data['Primary drinking water source']!=7), 'safe water']=0
          my_data.loc[(my_data['Primary drinking water source']!=11),'safe water'] = 0
          my_data.loc[(my_data['Primary drinking water source'] ==1),'safe water'] = 1
          my_data.loc[(my_data['Primary drinking water source'] ==7),'safe water'] = 1
          my_data.loc[(my_data['Primary drinking water source'] ==11),'safe water'] = 1

In [137]: my_data['safe water'].value_counts()

Out[137]: 1    71
          0    64
          Name: safe water, dtype: int64

In [138]: my_data[my_data.Roma==0]['safe water'].value_counts()

Out[138]: 1    49
          0    49
          Name: safe water, dtype: int64

In [139]: my_data[my_data.Roma==1]['safe water'].value_counts()

Out[139]: 1    22
          0    15
          Name: safe water, dtype: int64

In [140]: table_8 = epipy.create_2x2(my_data, row='Roma', column='safe water', row_order=[0,1],
                                 col_order=[0, 1])

In [141]: table_8

Out[141]:       0   1  All
          0    49  49   98
          1    15  22   37
          All  64  71  135

In [142]: epipy.analyze_2x2(table_8)

Odds ratio: 1.47 (95% CI: (0.68, 3.16))
Relative risk: 1.23 (95% CI: (0.8, 1.91))

Chi square: 0.963953059574
p value: 0.915211731536


In [143]: stats.ttest_ind(my_data[my_data.Roma==1]['safe water'],my_data[my_data.Roma==0]['safe

Out[143]: Ttest_indResult(statistic=0.97800924037239723, pvalue=0.32984534952286626)

In [144]: my_data['safe water'][my_data.Roma==1].var()
```

```
Out[144]: 0.24774774774774772

In [145]: my_data['safe water'][my_data.Roma==0].var()

Out[145]: 0.25257731958762886

In [146]: table_9 = epipy.create_2x2(my_data, row='Expense_person', column='safe water', row_ord
                          col_order=[0, 1])

In [147]: table_9

Out[147]:       0   1  All
          0    30  40   70
          1    34  31   65
          All  64  71  135

In [148]: epipy.analyze_2x2(table_9)

Odds ratio: 0.68 (95% CI: (0.35, 1.35))
Relative risk: 0.82 (95% CI: (0.57, 1.17))

Chi square: 1.20731601145
p value: 0.876892621614


In [149]: my_data.groupby('Community')['safe water'].value_counts()

Out[149]: Community  safe water
          1          1             20
                     0              9
          2          1             24
                     0              6
          3          0             16
                     1             14
          4          0             20
                     1             10
          5          0             13
                     1              3
          dtype: int64

In [150]: my_data['Distance of primary drinking water source']

Out[150]: 0      1
          1      3
          2      1
          3      1
          4      1
          5      1
          6      1
          7      1
```

8	1
9	1
10	1
11	1
12	1
13	1
14	1
15	1
16	1
17	1
18	1
19	1
20	1
21	1
22	1
23	1
24	1
25	1
26	1
27	1
28	1
29	1
	..
105	2
106	2
107	2
108	1
109	2
110	1
111	1
112	1
113	1
114	1
115	1
116	2
117	2
118	1
119	1
120	1
121	1
122	1
123	1
124	1
125	1
126	1
127	1
128	1
129	1

```
130    1
131    1
132    1
133    1
134    1
Name: Distance of primary drinking water source, dtype: int64
```

```
In [151]: my_data.groupby('Community')['Distance of primary drinking water source'].value_counts
```

```
Out[151]: Community  Distance of primary drinking water source
          1          1                                            28
                     3                                             1
          2          1                                            26
                     3                                             4
          3          1                                            22
                     2                                             8
          4          1                                            21
                     2                                             8
                     3                                             1
          5          1                                            16
          dtype: int64
```

```
In [152]: my_data[my_data.Roma==0]['Distance of primary drinking water source'].value_counts() #
```

```
Out[152]: 1    82
          2    12
          3     4
          Name: Distance of primary drinking water source, dtype: int64
```

```
In [153]: my_data[my_data.Roma==1]['Distance of primary drinking water source'].value_counts() #
```

```
Out[153]: 1    31
          2     4
          3     2
          Name: Distance of primary drinking water source, dtype: int64
```

```
In [154]: my_data['Length of time to collect water'].value_counts()
```

```
Out[154]: 2    99
          1    19
          3    10
          5     7
          Name: Length of time to collect water, dtype: int64
```

```
In [155]: my_data['Time to water']=0 #zero is less than 30 minutes to get the water you need
```

```
In [156]: my_data.loc[(my_data['Length of time to collect water']==1), 'Time to water']=0
          my_data.loc[(my_data['Length of time to collect water']==2), 'Time to water']=0
          my_data.loc[(my_data['Length of time to collect water']==3), 'Time to water']=1
          my_data.loc[(my_data['Length of time to collect water']==5), 'Time to water']=1
```

```
In [157]: my_data['Time to water'].value_counts()

Out[157]: 0    118
          1     17
          Name: Time to water, dtype: int64

In [158]: table_10 = epipy.create_2x2(my_data, row='Roma', column='Time to water', row_order=[0,
                                      col_order=[0, 1])

In [159]: table_10

Out[159]:        0   1  All
          0     86  12   98
          1     32   5   37
          All  118  17  135

In [160]: epipy.analyze_2x2(table_10)

Odds ratio: 1.12 (95% CI: (0.37, 3.43))
Relative risk: 1.01 (95% CI: (0.88, 1.18))

Chi square: 0.0392726948773
p value: 0.999809712276


In [161]: my_data['Bathroom'].value_counts()

Out[161]: 3    105
          1     28
          2      2
          Name: Bathroom, dtype: int64

In [162]: my_data.groupby('Roma')['Bathroom'].value_counts()

Out[162]: Roma  Bathroom
          0     3           76
                1           20
                2            2
          1     3           29
                1            8
          dtype: int64

In [163]: my_data.groupby('Community')['Bathroom'].value_counts()

Out[163]: Community  Bathroom
          1          3           23
                     1            5
                     2            1
          2          3           25
                     1            5
```

```
3          3           24
           1            6
4          3           25
           1            5
5          3            8
           1            7
           2            1
dtype: int64
```

In [164]:
```
my_data['Toilet facility'].value_counts()
```

Out[164]:
```
4    98
2    16
1     9
3     6
8     5
5     1
Name: Toilet facility, dtype: int64
```

In [165]:
```
my_data.groupby('Roma')['Toilet facility'].value_counts()
```

Out[165]:
```
Roma  Toilet facility
0     4                  71
      2                  12
      1                   5
      8                   5
      3                   4
      5                   1
1     4                  27
      1                   4
      2                   4
      3                   2
dtype: int64
```

In [166]:
```
my_data['proper toilet']=0
```

In [167]:
```
my_data.loc[(my_data['Toilet facility']==1),'proper toilet']=1 #toilet facility is seu
my_data.loc[(my_data['Toilet facility']==2),'proper toilet']=1
my_data.loc[(my_data['Toilet facility']==3),'proper toilet']=1
my_data.loc[(my_data['Toilet facility']==4),'proper toilet']=0 #toilet facility is a p
my_data.loc[(my_data['Toilet facility']==5),'proper toilet']=0
my_data.loc[(my_data['Toilet facility']==8),'proper toilet']=0
```

In [168]:
```
my_data['proper toilet'].value_counts()
```

Out[168]:
```
0    104
1     31
Name: proper toilet, dtype: int64
```

In [169]:
```
my_data[my_data.Roma==0]['proper toilet'].value_counts()
```

```
Out[169]: 0    77
          1    21
          Name: proper toilet, dtype: int64
```

```
In [170]: my_data[my_data.Roma==1]['proper toilet'].value_counts()
```

```
Out[170]: 0    27
          1    10
          Name: proper toilet, dtype: int64
```

```
In [171]: table_11 = epipy.create_2x2(my_data, row='Roma', column='proper toilet', row_order=[0,
                              col_order=[0, 1])
```

```
In [172]: table_11
```

```
Out[172]:        0   1  All
          0     77  21   98
          1     27  10   37
          All  104  31  135
```

```
In [173]: epipy.analyze_2x2(table_11)
```

```
Odds ratio: 1.36 (95% CI: (0.57, 3.25))
Relative risk: 1.08 (95% CI: (0.86, 1.34))

Chi square: 0.475886090805
p value: 0.97580561484
```

```
In [174]: my_data['Diarrhea in last year'].value_counts()
```

```
Out[174]: 1    72
          0    63
          Name: Diarrhea in last year, dtype: int64
```

```
In [175]: my_data.groupby('Roma')['Diarrhea in last year'].value_counts()
```

```
Out[175]: Roma  Diarrhea in last year
          0     1                        57
                0                        41
          1     0                        22
                1                        15
          dtype: int64
```

```
In [176]: table_12=epipy.create_2x2(my_data, row='Roma', column='Diarrhea in last year', row_ord
                              col_order=[1,0]) #columns: 1 is yes and 0 is no (one family member at
```

```
In [177]: table_12
```

```
Out[177]:       1   0  All
          0    57  41   98
          1    15  22   37
          All  72  63  135

In [178]: epipy.analyze_2x2(table_12)

Odds ratio: 2.04 (95% CI: (0.94, 4.4))
Relative risk: 1.43 (95% CI: (0.94, 2.19))

Chi square: 3.3514646206
p value: 0.500819658108


In [179]: table_13=epipy.create_2x2(my_data, row='proper toilet', column='Diarrhea in last year'
                                col_order=[1,0]) #1=proper toilet protected from leakage 0=not a prop

In [180]: table_13

Out[180]:       1   0  All
          0    58  46  104
          1    14  17   31
          All  72  63  135

In [181]: epipy.analyze_2x2(table_13)

Odds ratio: 1.53 (95% CI: (0.68, 3.43))
Relative risk: 1.23 (95% CI: (0.81, 1.89))

Chi square: 1.07973679546
p value: 0.89747371947


In [182]: my_data['Immunized'].value_counts() #yes=1, no=0 #Less than 90% immunized against theo

Out[182]: 1    120
          0     15
          Name: Immunized, dtype: int64

In [183]: my_data.groupby('Roma')['Immunized'].value_counts()

Out[183]: Roma  Immunized
          0     1            86
                0            12
          1     1            34
                0             3
          dtype: int64

In [184]: table_14=epipy.create_2x2(my_data, row='Roma', column='Immunized', row_order=[0,1],
                                col_order=[0,1]) #columns are 1 = yes and 0=No, rows are 0=roma 1=not
```

```
In [185]: table_14

Out[185]:       0    1  All
          0    12   86   98
          1     3   34   37
          All  15  120  135

In [186]: epipy.analyze_2x2(table_14)

Odds ratio: 1.58 (95% CI: (0.42, 5.96))
Relative risk: 1.51 (95% CI: (0.45, 5.05))

Chi square: 0.465388858246
p value: 0.976781727544


In [187]: my_data['Medical Insurance'].value_counts()

Out[187]: 1    113
          0     22
          Name: Medical Insurance, dtype: int64

In [188]: my_data.groupby('Roma')['Medical Insurance'].value_counts()

Out[188]: Roma  Medical Insurance
          0     1                    80
                0                    18
          1     1                    33
                0                     4
          dtype: int64

In [189]: table_15=epipy.create_2x2(my_data, row='Roma', column='Medical Insurance', row_order=[
                           col_order=[0,1]) #columns 1=yes, 0=no

In [190]: table_15

Out[190]:       0    1  All
          0    18   80   98
          1     4   33   37
          All  22  113  135

In [191]: epipy.analyze_2x2(table_15)

Odds ratio: 1.86 (95% CI: (0.58, 5.9))
Relative risk: 1.7 (95% CI: (0.62, 4.69))

Chi square: 1.12436150995
p value: 0.890387969872
```

```
In [192]: my_data['Belief in safety of primary drinking water source'].value_counts() #1 is yes,

Out[192]: 1    93
          2    42
          Name: Belief in safety of primary drinking water source, dtype: int64

In [193]: my_data.groupby('Roma')['Belief in safety of primary drinking water source'].value_cou

Out[193]: Roma  Belief in safety of primary drinking water source
          0     1                                                     62
                2                                                     36
          1     1                                                     31
                2                                                      6
          dtype: int64

In [194]: table_16=epipy.create_2x2(my_data, row='Roma', column='Belief in safety of primary dri
                                 col_order=[2,1]) #columsn are 1=safe and 2=not safe

In [195]: table_16

Out[195]:        2   1  All
          0     36  62   98
          1      6  31   37
          All   42  93  135

In [196]: epipy.analyze_2x2(table_16)

Odds ratio: 3.0 (95% CI: (1.14, 7.88))
Relative risk: 2.27 (95% CI: (1.04, 4.93))

Chi square: 5.27617997006
p value: 0.260115069601


In [197]: my_data['Satisfaction level with water'].value_counts()

Out[197]: 1    64
          2    39
          3    30
          4     2
          Name: Satisfaction level with water, dtype: int64

In [198]: my_data.groupby('Roma')['Satisfaction level with water'].value_counts()

Out[198]: Roma  Satisfaction level with water
          0     1                                 45
                3                                 27
                2                                 24
                4                                  2
          1     1                                 19
                2                                 15
                3                                  3
          dtype: int64
```

```
In [199]: my_data['Water satisfaction']=0

In [200]: my_data.loc[(my_data['Satisfaction level with water']==1), 'Water satisfaction']=0
          my_data.loc[(my_data['Satisfaction level with water']==2), 'Water satisfaction']=1
          my_data.loc[(my_data['Satisfaction level with water']==3), 'Water satisfaction']=1
          my_data.loc[(my_data['Satisfaction level with water']==4), 'Water satisfaction']=1 #0

In [201]: my_data['Water satisfaction']

Out[201]: 0      0
          1      1
          2      1
          3      0
          4      0
          5      1
          6      0
          7      1
          8      1
          9      0
          10     1
          11     0
          12     0
          13     0
          14     0
          15     0
          16     0
          17     0
          18     0
          19     0
          20     1
          21     0
          22     1
          23     0
          24     0
          25     1
          26     0
          27     0
          28     0
          29     0
                ..
          105    1
          106    1
          107    0
          108    0
          109    1
          110    1
          111    1
          112    1
```

```
113    1
114    1
115    1
116    1
117    1
118    1
119    1
120    1
121    1
122    1
123    1
124    0
125    0
126    1
127    1
128    1
129    1
130    1
131    1
132    1
133    1
134    1
Name: Water satisfaction, dtype: int64
```

```
In [202]: table_17=epipy.create_2x2(my_data, row='Roma', column='Water satisfaction', row_order=
                              col_order=[1,0]) #rows are 0=roma, 1=not and columns are 0=generally
```

```
In [203]: table_17
```

```
Out[203]:        1   0  All
          0     53  45   98
          1     18  19   37
          All   71  64  135
```

```
In [204]: epipy.analyze_2x2(table_17)
```

```
Odds ratio: 1.24 (95% CI: (0.58, 2.65))
Relative risk: 1.11 (95% CI: (0.76, 1.62))

Chi square: 0.317980214783
p value: 0.988624121709
```

```
In [208]: stats.ttest_ind(my_data[my_data.Roma==1].Education,my_data[my_data.Roma==0].Education)
```

```
Out[208]: Ttest_indResult(statistic=4.1489154678417828, pvalue=5.9230206739533357e-05)
```

```
In [209]: my_data.Education[my_data.Roma==1].var()
```

```
Out[209]: 5.6636636636636641
```

```
In [210]: my_data.Education[my_data.Roma==0].var()

Out[210]: 5.4644435093625079

In [211]: my_data['Household size'][my_data.Roma==1].var()

Out[211]: 4.091591591591591

In [212]: my_data['Household size'][my_data.Roma==0].var()

Out[212]: 6.5610140963601928

In [213]: stats.ttest_ind(my_data[my_data.Roma==1]['Household size'],my_data[my_data.Roma==0]['H

Out[213]: Ttest_indResult(statistic=-3.3437795805782926, pvalue=0.0010738692766787267)

In [214]: my_data['Household size'][my_data.Roma==1].describe()

Out[214]: count    37.000000
          mean      3.729730
          std       2.022768
          min       1.000000
          25%       2.000000
          50%       3.000000
          75%       5.000000
          max       9.000000
          Name: Household size, dtype: float64

In [215]: my_data['Household size'][my_data.Roma==0].describe()

Out[215]: count    98.000000
          mean      5.295918
          std       2.561448
          min       1.000000
          25%       3.000000
          50%       5.000000
          75%       7.000000
          max      12.000000
          Name: Household size, dtype: float64

In [216]: stats.ttest_ind(my_data[my_data.Roma==1]['Employment status'],my_data[my_data.Roma==0]

Out[216]: Ttest_indResult(statistic=-0.67622831308602316, pvalue=0.500069952165482)

In [217]: my_data['Employment status'][my_data.Roma==0].var()

Out[217]: 0.19692825583841783

In [218]: my_data['Employment status'][my_data.Roma==1].var()

Out[218]: 0.2252252252252252
```

```
In [219]: my_data['Occupation']

Out[219]: 0      3
          1      1
          2      1
          3      5
          4      4
          5      4
          6      3
          7      4
          8      4
          9      4
          10     4
          11     3
          12     4
          13     6
          14     1
          15     3
          16     3
          17     3
          18     2
          19     3
          20     4
          21     4
          22     4
          23     4
          24     4
          25     3
          26     4
          27     4
          28     4
          29     3
                ..
          105    4
          106    4
          107    5
          108    4
          109    1
          110    1
          111    4
          112    4
          113    2
          114    3
          115    3
          116    3
          117    4
          118    4
          119    1
```

```
120    1
121    6
122    2
123    1
124    1
125    1
126    2
127    1
128    1
129    1
130    1
131    1
132    1
133    4
134    1
Name: Occupation, dtype: int64
```

In [220]:
```
my_data['Employment type']=0 #unemployed = 0, pension or student = 1, part time or day
my_data.loc[(my_data['Occupation']==4), 'Employment type']=0
my_data.loc[(my_data['Occupation']==5), 'Employment type']=0
my_data.loc[(my_data['Occupation']==6), 'Employment type']=1
my_data.loc[(my_data['Occupation']==3), 'Employment type']=1
my_data.loc[(my_data['Occupation']==2), 'Employment type']=2
my_data.loc[(my_data['Occupation']==1), 'Employment type']=3
```

In [221]:
```
stats.ttest_ind(my_data[my_data.Roma==1]['Employment type'],my_data[my_data.Roma==0]['
```

Out[221]:
```
Ttest_indResult(statistic=0.18516890210900822, pvalue=0.85337863839211892)
```

In [222]:
```
my_data['Employment type'][my_data.Roma==0].var()
```

Out[222]:
```
1.6191878813381018
```

In [223]:
```
my_data['Employment type'][my_data.Roma==1].var()
```

Out[223]:
```
1.7027027027027031
```

In [224]:
```
my_data['Geography']=0 #refers to Urban vs. rural communities (sub)-urban = 1 and rura
```

In [225]:
```
my_data.loc[(my_data['Community']==1),'Geography']=2
my_data.loc[(my_data['Community']==2),'Geography']=2
my_data.loc[(my_data['Community']==3),'Geography']=1
my_data.loc[(my_data['Community']==4),'Geography']=2
my_data.loc[(my_data['Community']==5),'Geography']=1
```

In [226]:
```
stats.ttest_ind(my_data[my_data.Geography==1]['Employment type'],my_data[my_data.Geogr
```

Out[226]:
```
Ttest_indResult(statistic=2.7837086193932903, pvalue=0.0061576957808519165)
```

In [227]:
```
my_data['Employment type'][my_data.Geography==1].var()
```

```
Out[227]: 1.8318840579710149

In [228]: my_data['Employment type'][my_data.Geography==2].var()

Out[228]: 1.408580183861083

In [229]: stats.ttest_ind(my_data[my_data.Geography==1]['Expense_person'],my_data[my_data.Geogra

Out[229]: Ttest_indResult(statistic=3.323024975681669, pvalue=0.001150348561918845)

In [230]: my_data['Expense_person'][my_data.Geography==1].var()

Out[230]: 0.2246376811594202

In [231]: my_data['Expense_person'][my_data.Geography==2].var()

Out[231]: 0.23876404494382023

In [232]: stats.ttest_ind(my_data[my_data.Roma==1]['Distance of primary drinking water source'],

Out[232]: Ttest_indResult(statistic=0.12403858845472203, pvalue=0.90147198774648318)

In [233]: my_data['Distance of primary drinking water source'][my_data.Roma==0].var()

Out[233]: 0.24658110666947192

In [234]: my_data['Distance of primary drinking water source'][my_data.Roma==1].var()

Out[234]: 0.28528528528528529

In [235]: stats.ttest_ind(my_data[my_data.Geography==1]['Distance of primary drinking water sour

Out[235]: Ttest_indResult(statistic=-0.55243353366014969, pvalue=0.58157856654422813)

In [236]: my_data['Time to water']=0
          my_data.loc[(my_data['Length of time to collect water']==1),'Time to water']=0
          my_data.loc[(my_data['Length of time to collect water']==2),'Time to water']=1
          my_data.loc[(my_data['Length of time to collect water']==3),'Time to water']=2
          my_data.loc[(my_data['Length of time to collect water']==4),'Time to water']=3
          my_data.loc[(my_data['Length of time to collect water']==5),'Time to water']=4

In [237]: stats.ttest_ind(my_data[my_data.Roma==1]['Time to water'],my_data[my_data.Roma==0]['Ti

Out[237]: Ttest_indResult(statistic=-0.7691840636904056, pvalue=0.4431478867118408)

In [238]: stats.ttest_ind(my_data[my_data.Geography==1]['Time to water'],my_data[my_data.Geograp

Out[238]: Ttest_indResult(statistic=1.3064576704732969, pvalue=0.19365169365049323)
```

```
In [239]: #multivar_model = sm.formula.glm(['Roma']~['Property documents']+['Education_level']+[
          #multivar_model = sm.formula.glm('Roma~Property documents+Education_level+Family size+
          my_dataRK = my_data
          my_dataRK.columns = [column.replace(' ','_') for column in my_dataRK.columns]
          multivar_model = sm.formula.glm('Roma~Property_documents + Education_level + Family_si


          print my_data.keys()


          multivar_model.summary()
```

```
Index([u'Survey', u'Community', u'Household_size', u'Time_lived_there',
       u'Head_of_house', u'50+_men', u'50+_women', u'15-49_men',
       u'15-49_women', u'under_15_boys', u'under_15_girls',
       u'literate_men_(+15)', u'literate_women_(+15)', u'Religion', u'Gender',
       u'Age', u'Marital_status', u'Education', u'Occupation', u'Ethnicity',
       u'Drinking_water_sources_available', u'Primary_drinking_water_source',
       u'Primary_non-drinking_water_source',
       u'Who_uses_primary_non-drinking_water_source',
       u'Belief_in_safety_of_primary_drinking_water_source',
       u'Distance_of_primary_drinking_water_source',
       u'Length_of_time_to_collect_water', u'Pay_for_water',
       u'Water_treatment', u'Type_of_water_treatment',
       u'Satisfaction_level_with_water', u'Time_on_household_duties',
       u'Spouse_time_on_household_duties', u'Children_attend_school',
       u'Bathroom', u'Toilet_facility', u'Shared_toilet',
       u'Diarrhea_in_last_year', u'Diarrhea_number_of_people', u'Immunized',
       u'Children_immunized', u'Primary_care_physician', u'Medical_Insurance',
       u'Last_doctor_visit', u'Child_to_doctor', u'Land_for_personal_use',
       u'Animals_owned', u'weekly_expenses_(RON)', u'Property_documents',
       u'Items_owned', u'Electricity', u'Piped__tank_gas',
       u'Decider_for_female_earned_money', u'Conflict_between_Roma_and_non',
       u'Roma_segregated', u'Community_helpful', u'House', u'Roof', u'Floor',
       u'Housing_Score', u'Roma', u'Education_level', u'Family_size',
       u'Marital_status_simple', u'Employment_status', u'Total_men',
       u'Total_women', u'illiterate_men', u'illiterate_women',
       u'WeeklyExpense_perPerson', u'Expense_person', u'tap_water_availabe',
       u'tap_water_available', u'safe_water', u'Time_to_water',
       u'proper_toilet', u'Water_satisfaction', u'Employment_type',
       u'Geography'],
      dtype='object')
```

```
Out[239]: <class 'statsmodels.iolib.summary.Summary'>
          """
                            Generalized Linear Model Regression Results
          ==============================================================================
```

```
Dep. Variable:                   Roma   No. Observations:                  129
Model:                            GLM   Df Residuals:                      124
Model Family:                Gaussian   Df Model:                            4
Link Function:               identity   Scale:                   0.17306315844
Method:                          IRLS   Log-Likelihood:                -67.354
Date:            Tue, 14 Mar 2017   Deviance:                       21.460
Time:                        12:46:52   Pearson chi2:                     21.5
No. Iterations:                     4
==================================================================================
                      coef    std err          z      P>|z|      [95.0% Conf. Int.]
----------------------------------------------------------------------------------
Intercept           0.7549      0.146      5.163      0.000         0.468     1.041
Property_documents -0.0854      0.079     -1.084      0.279        -0.240     0.069
Education_level    -0.2613      0.082     -3.180      0.001        -0.422    -0.100
Family_size        -0.2362      0.078     -3.026      0.002        -0.389    -0.083
Employment_status  -0.0505      0.086     -0.585      0.559        -0.220     0.119
==================================================================================
"""
```

```
In [240]: my_dataRK['MV_Roma']=0
          my_dataRK.loc[(my_dataRK['Roma']==0), 'MV_Roma']=1
          my_dataRK.loc[(my_dataRK['Roma']==1), 'MV_Roma']=0

In [241]: my_dataRK['Improved_sanitation']=0

In [242]: my_dataRK.loc[(my_dataRK['Toilet_facility']==1),'Improved_sanitation']=1
          my_dataRK.loc[(my_dataRK['Toilet_facility']==2),'Improved_sanitation']=1

In [243]: my_dataRK['Sanitation_two']=0
          my_dataRK.loc[(my_dataRK['Bathroom']==1), 'Sanitation_two']=1

In [244]: my_dataRK['Improved_water']=0
          my_dataRK.loc[(my_dataRK['Primary_drinking_water_source']==1), 'Improved_water']=1
          my_dataRK.loc[(my_dataRK['Primary_drinking_water_source']==11), 'Improved_water']=1

In [245]: my_dataRK['Modified_WASH_Score']= my_dataRK['Improved_sanitation'] + my_dataRK['Sanita

In [246]: my_dataRK['Modified_WASH_Score'][my_dataRK.Roma==1].value_counts()

Out[246]: 0    26
          1     5
          2     4
          3     2
          Name: Modified_WASH_Score, dtype: int64

In [247]: my_dataRK['Modified_WASH_Score'][my_dataRK.Roma==0].value_counts()

Out[247]: 0    62
          2    17
          1    17
          3     2
          Name: Modified_WASH_Score, dtype: int64
```

```
In [248]: my_dataRK['Diarrhea']=0
          my_dataRK.loc[(my_dataRK['Diarrhea_in_last_year'])==1, 'Diarrhea']=0
          my_dataRK.loc[(my_dataRK['Diarrhea_in_last_year'])==0, 'Diarrhea']=1

In [249]: my_dataRK['Healthcare_Score']= my_dataRK['Primary_care_physician'] + my_dataRK['Medica

In [250]: my_dataRK['Healthcare_Score'][my_dataRK.Roma==1].value_counts()

Out[250]: 4    20
          3    11
          2     6
          Name: Healthcare_Score, dtype: int64

In [251]: my_dataRK['Healthcare_Score'][my_dataRK.Roma==0].value_counts()

Out[251]: 3    43
          4    34
          2    17
          1     4
          Name: Healthcare_Score, dtype: int64

In [252]: my_dataRK['MV_Healthcare_Score']=0
          my_dataRK['MV_Healthcare_Score']=(4-my_dataRK['Healthcare_Score'])/4

In [253]: my_dataRK['MV_Healthcare_Score'][my_dataRK.Roma==0].value_counts()

Out[253]: 0.25    43
          0.00    34
          0.50    17
          0.75     4
          Name: MV_Healthcare_Score, dtype: int64

In [254]: multivar_model = sm.formula.glm('Roma~Healthcare_Score + Modified_WASH_Score + Geograp


          print my_data.keys()


          multivar_model.summary()

Index([u'Survey', u'Community', u'Household_size', u'Time_lived_there',
       u'Head_of_house', u'50+_men', u'50+_women', u'15-49_men',
       u'15-49_women', u'under_15_boys', u'under_15_girls',
       u'literate_men_(+15)', u'literate_women_(+15)', u'Religion', u'Gender',
       u'Age', u'Marital_status', u'Education', u'Occupation', u'Ethnicity',
       u'Drinking_water_sources_available', u'Primary_drinking_water_source',
       u'Primary_non-drinking_water_source',
       u'Who_uses_primary_non-drinking_water_source',
       u'Belief_in_safety_of_primary_drinking_water_source',
```

```
        u'Distance_of_primary_drinking_water_source',
        u'Length_of_time_to_collect_water', u'Pay_for_water',
        u'Water_treatment', u'Type_of_water_treatment',
        u'Satisfaction_level_with_water', u'Time_on_household_duties',
        u'Spouse_time_on_household_duties', u'Children_attend_school',
        u'Bathroom', u'Toilet_facility', u'Shared_toilet',
        u'Diarrhea_in_last_year', u'Diarrhea_number_of_people', u'Immunized',
        u'Children_immunized', u'Primary_care_physician', u'Medical_Insurance',
        u'Last_doctor_visit', u'Child_to_doctor', u'Land_for_personal_use',
        u'Animals_owned', u'weekly_expenses_(RON)', u'Property_documents',
        u'Items_owned', u'Electricity', u'Piped__tank_gas',
        u'Decider_for_female_earned_money', u'Conflict_between_Roma_and_non',
        u'Roma_segregated', u'Community_helpful', u'House', u'Roof', u'Floor',
        u'Housing_Score', u'Roma', u'Education_level', u'Family_size',
        u'Marital_status_simple', u'Employment_status', u'Total_men',
        u'Total_women', u'illiterate_men', u'illiterate_women',
        u'WeeklyExpense_perPerson', u'Expense_person', u'tap_water_availabe',
        u'tap_water_available', u'safe_water', u'Time_to_water',
        u'proper_toilet', u'Water_satisfaction', u'Employment_type',
        u'Geography', u'MV_Roma', u'Improved_sanitation', u'Sanitation_two',
        u'Improved_water', u'Modified_WASH_Score', u'Diarrhea',
        u'Healthcare_Score', u'MV_Healthcare_Score'],
       dtype='object')
```

```
Out[254]: <class 'statsmodels.iolib.summary.Summary'>
"""
                 Generalized Linear Model Regression Results
==============================================================================
Dep. Variable:                   Roma   No. Observations:                  135
Model:                            GLM   Df Residuals:                      131
Model Family:                Gaussian   Df Model:                            3
Link Function:               identity   Scale:                  0.199430675587
Method:                          IRLS   Log-Likelihood:                -80.697
Date:                Tue, 14 Mar 2017   Deviance:                       26.125
Time:                        12:46:52   Pearson chi2:                     26.1
No. Iterations:                     4
================================================================================
                          coef    std err          z      P>|z|      [95.0% Conf. Int.
--------------------------------------------------------------------------------
Intercept               0.0267      0.206      0.130      0.897        -0.376     0.43
Healthcare_Score        0.0887      0.047      1.867      0.062        -0.004     0.18
Modified_WASH_Score    -0.0255      0.045     -0.567      0.571        -0.114     0.06
Geography              -0.0117      0.081     -0.144      0.885        -0.171     0.14
================================================================================
"""
```

```
In [255]: multivar_model = sm.formula.glm('Roma~Healthcare_Score + Modified_WASH_Score + Expense
```

```
print my_data.keys()

multivar_model.summary()
```

```
Index([u'Survey', u'Community', u'Household_size', u'Time_lived_there',
       u'Head_of_house', u'50+_men', u'50+_women', u'15-49_men',
       u'15-49_women', u'under_15_boys', u'under_15_girls',
       u'literate_men_(+15)', u'literate_women_(+15)', u'Religion', u'Gender',
       u'Age', u'Marital_status', u'Education', u'Occupation', u'Ethnicity',
       u'Drinking_water_sources_available', u'Primary_drinking_water_source',
       u'Primary_non-drinking_water_source',
       u'Who_uses_primary_non-drinking_water_source',
       u'Belief_in_safety_of_primary_drinking_water_source',
       u'Distance_of_primary_drinking_water_source',
       u'Length_of_time_to_collect_water', u'Pay_for_water',
       u'Water_treatment', u'Type_of_water_treatment',
       u'Satisfaction_level_with_water', u'Time_on_household_duties',
       u'Spouse_time_on_household_duties', u'Children_attend_school',
       u'Bathroom', u'Toilet_facility', u'Shared_toilet',
       u'Diarrhea_in_last_year', u'Diarrhea_number_of_people', u'Immunized',
       u'Children_immunized', u'Primary_care_physician', u'Medical_Insurance',
       u'Last_doctor_visit', u'Child_to_doctor', u'Land_for_personal_use',
       u'Animals_owned', u'weekly_expenses_(RON)', u'Property_documents',
       u'Items_owned', u'Electricity', u'Piped__tank_gas',
       u'Decider_for_female_earned_money', u'Conflict_between_Roma_and_non',
       u'Roma_segregated', u'Community_helpful', u'House', u'Roof', u'Floor',
       u'Housing_Score', u'Roma', u'Education_level', u'Family_size',
       u'Marital_status_simple', u'Employment_status', u'Total_men',
       u'Total_women', u'illiterate_men', u'illiterate_women',
       u'WeeklyExpense_perPerson', u'Expense_person', u'tap_water_availabe',
       u'tap_water_available', u'safe_water', u'Time_to_water',
       u'proper_toilet', u'Water_satisfaction', u'Employment_type',
       u'Geography', u'MV_Roma', u'Improved_sanitation', u'Sanitation_two',
       u'Improved_water', u'Modified_WASH_Score', u'Diarrhea',
       u'Healthcare_Score', u'MV_Healthcare_Score'],
      dtype='object')
```

Out[255]:
```
<class 'statsmodels.iolib.summary.Summary'>
"""
                 Generalized Linear Model Regression Results
==============================================================================
Dep. Variable:                   Roma   No. Observations:                  135
Model:                            GLM   Df Residuals:                      131
Model Family:                Gaussian   Df Model:                            3
Link Function:               identity   Scale:                  0.197584215263
```

```
Method:                          IRLS   Log-Likelihood:                -80.069
Date:                Tue, 14 Mar 2017   Deviance:                       25.884
Time:                        12:46:52   Pearson chi2:                     25.9
No. Iterations:                     4
==================================================================================
                          coef    std err          z      P>|z|      [95.0% Conf. Int.
----------------------------------------------------------------------------------
Intercept              -0.0181      0.156     -0.116      0.908        -0.324     0.28
Healthcare_Score        0.0843      0.047      1.776      0.076        -0.009     0.17
Modified_WASH_Score    -0.0291      0.045     -0.648      0.517        -0.117     0.05
Expense_person          0.0860      0.077      1.116      0.264        -0.065     0.23
==================================================================================
"""
```

In [256]:
```
my_dataRK['Poverty_Score']= my_dataRK['Electricity'] + my_dataRK['Piped__tank_gas'] +
```

In [257]:
```
my_dataRK['MV_Poverty_Score']=0
my_dataRK['MV_Poverty_Score']=(3-my_dataRK['Poverty_Score'])/3
```

In [258]:
```
my_dataRK['MV_Poverty_Score'][my_dataRK.Roma==0].value_counts()
```

Out[258]:
```
0.333333    46
0.000000    29
0.666667    16
1.000000     7
Name: MV_Poverty_Score, dtype: int64
```

In [259]:
```
multivar_model = sm.formula.glm('Roma~Healthcare_Score + Modified_WASH_Score + Poverty


print my_data.keys()


multivar_model.summary()
```

```
Index([u'Survey', u'Community', u'Household_size', u'Time_lived_there',
       u'Head_of_house', u'50+_men', u'50+_women', u'15-49_men',
       u'15-49_women', u'under_15_boys', u'under_15_girls',
       u'literate_men_(+15)', u'literate_women_(+15)', u'Religion', u'Gender',
       u'Age', u'Marital_status', u'Education', u'Occupation', u'Ethnicity',
       u'Drinking_water_sources_available', u'Primary_drinking_water_source',
       u'Primary_non-drinking_water_source',
       u'Who_uses_primary_non-drinking_water_source',
       u'Belief_in_safety_of_primary_drinking_water_source',
       u'Distance_of_primary_drinking_water_source',
       u'Length_of_time_to_collect_water', u'Pay_for_water',
       u'Water_treatment', u'Type_of_water_treatment',
       u'Satisfaction_level_with_water', u'Time_on_household_duties',
       u'Spouse_time_on_household_duties', u'Children_attend_school',
```

```
       u'Bathroom', u'Toilet_facility', u'Shared_toilet',
       u'Diarrhea_in_last_year', u'Diarrhea_number_of_people', u'Immunized',
       u'Children_immunized', u'Primary_care_physician', u'Medical_Insurance',
       u'Last_doctor_visit', u'Child_to_doctor', u'Land_for_personal_use',
       u'Animals_owned', u'weekly_expenses_(RON)', u'Property_documents',
       u'Items_owned', u'Electricity', u'Piped__tank_gas',
       u'Decider_for_female_earned_money', u'Conflict_between_Roma_and_non',
       u'Roma_segregated', u'Community_helpful', u'House', u'Roof', u'Floor',
       u'Housing_Score', u'Roma', u'Education_level', u'Family_size',
       u'Marital_status_simple', u'Employment_status', u'Total_men',
       u'Total_women', u'illiterate_men', u'illiterate_women',
       u'WeeklyExpense_perPerson', u'Expense_person', u'tap_water_availabe',
       u'tap_water_available', u'safe_water', u'Time_to_water',
       u'proper_toilet', u'Water_satisfaction', u'Employment_type',
       u'Geography', u'MV_Roma', u'Improved_sanitation', u'Sanitation_two',
       u'Improved_water', u'Modified_WASH_Score', u'Diarrhea',
       u'Healthcare_Score', u'MV_Healthcare_Score', u'Poverty_Score',
       u'MV_Poverty_Score'],
      dtype='object')
```

Out[259]:
```
<class 'statsmodels.iolib.summary.Summary'>
"""
                 Generalized Linear Model Regression Results
==============================================================================
Dep. Variable:                   Roma   No. Observations:                  135
Model:                            GLM   Df Residuals:                      131
Model Family:                Gaussian   Df Model:                            3
Link Function:               identity   Scale:                  0.192410703752
Method:                          IRLS   Log-Likelihood:                -78.278
Date:                Tue, 14 Mar 2017   Deviance:                       25.206
Time:                        12:46:52   Pearson chi2:                     25.2
No. Iterations:                     4
==========================================================================================
                          coef    std err          z      P>|z|      [95.0% Conf. Int.
------------------------------------------------------------------------------------------
Intercept              -0.1579      0.170     -0.928      0.354        -0.491      0.17
Healthcare_Score        0.0765      0.047      1.627      0.104        -0.016      0.16
Modified_WASH_Score    -0.0413      0.045     -0.923      0.356        -0.129      0.04
Poverty_Score           0.1019      0.046      2.191      0.028         0.011      0.19
==========================================================================================
"""
```

In [260]:
```
my_dataRK['MV_Poverty_Score'][my_dataRK.Roma==0].value_counts()
```

Out[260]:
```
0.333333    46
0.000000    29
0.666667    16
```

```
        1.000000     7
        Name: MV_Poverty_Score, dtype: int64
```

```
In [261]: my_dataRK['MV_Poverty_Score'][my_dataRK.Roma==1].value_counts()
```

```
Out[261]: 0.000000    17
          0.333333    16
          0.666667     4
          Name: MV_Poverty_Score, dtype: int64
```

```
In [262]: multivar_model = sm.formula.glm('Roma~MV_Healthcare_Score + MV_Poverty_Score', data=my


          print my_data.keys()


          multivar_model.summary()
```

```
Index([u'Survey', u'Community', u'Household_size', u'Time_lived_there',
       u'Head_of_house', u'50+_men', u'50+_women', u'15-49_men',
       u'15-49_women', u'under_15_boys', u'under_15_girls',
       u'literate_men_(+15)', u'literate_women_(+15)', u'Religion', u'Gender',
       u'Age', u'Marital_status', u'Education', u'Occupation', u'Ethnicity',
       u'Drinking_water_sources_available', u'Primary_drinking_water_source',
       u'Primary_non-drinking_water_source',
       u'Who_uses_primary_non-drinking_water_source',
       u'Belief_in_safety_of_primary_drinking_water_source',
       u'Distance_of_primary_drinking_water_source',
       u'Length_of_time_to_collect_water', u'Pay_for_water',
       u'Water_treatment', u'Type_of_water_treatment',
       u'Satisfaction_level_with_water', u'Time_on_household_duties',
       u'Spouse_time_on_household_duties', u'Children_attend_school',
       u'Bathroom', u'Toilet_facility', u'Shared_toilet',
       u'Diarrhea_in_last_year', u'Diarrhea_number_of_people', u'Immunized',
       u'Children_immunized', u'Primary_care_physician', u'Medical_Insurance',
       u'Last_doctor_visit', u'Child_to_doctor', u'Land_for_personal_use',
       u'Animals_owned', u'weekly_expenses_(RON)', u'Property_documents',
       u'Items_owned', u'Electricity', u'Piped__tank_gas',
       u'Decider_for_female_earned_money', u'Conflict_between_Roma_and_non',
       u'Roma_segregated', u'Community_helpful', u'House', u'Roof', u'Floor',
       u'Housing_Score', u'Roma', u'Education_level', u'Family_size',
       u'Marital_status_simple', u'Employment_status', u'Total_men',
       u'Total_women', u'illiterate_men', u'illiterate_women',
       u'WeeklyExpense_perPerson', u'Expense_person', u'tap_water_availabe',
       u'tap_water_available', u'safe_water', u'Time_to_water',
       u'proper_toilet', u'Water_satisfaction', u'Employment_type',
       u'Geography', u'MV_Roma', u'Improved_sanitation', u'Sanitation_two',
       u'Improved_water', u'Modified_WASH_Score', u'Diarrhea',
       u'Healthcare_Score', u'MV_Healthcare_Score', u'Poverty_Score',
```

```
        u'MV_Poverty_Score'],
       dtype='object')
```

Out[262]:
```
<class 'statsmodels.iolib.summary.Summary'>
"""
                 Generalized Linear Model Regression Results
==============================================================================
Dep. Variable:                   Roma   No. Observations:                  135
Model:                            GLM   Df Residuals:                      132
Model Family:                Gaussian   Df Model:                            2
Link Function:               identity   Scale:                  0.192194801096
Method:                          IRLS   Log-Likelihood:                -78.716
Date:                Tue, 14 Mar 2017   Deviance:                       25.370
Time:                        12:46:52   Pearson chi2:                     25.4
No. Iterations:                     4
==============================================================================
                          coef    std err          z      P>|z|      [95.0% Conf. Int.
------------------------------------------------------------------------------
Intercept               0.4217      0.065      6.466      0.000         0.294     0.55
MV_Healthcare_Score    -0.2946      0.187     -1.572      0.116        -0.662     0.07
MV_Poverty_Score       -0.2851      0.138     -2.071      0.038        -0.555    -0.01
==============================================================================
"""
```

In [263]:
```
my_dataRK['Insecure_Housing']=0
my_dataRK.loc[(my_dataRK['Housing_Score'])==0,'Insecure_Housing']=1
my_dataRK.loc[(my_dataRK['Housing_Score'])==1,'Insecure_Housing']=1
my_dataRK.loc[(my_dataRK['Housing_Score'])==2,'Insecure_Housing']=0
my_dataRK.loc[(my_dataRK['Housing_Score'])==3,'Insecure_Housing']=0
```

In [264]:
```
my_dataRK['WASH_Score']= my_dataRK['Improved_sanitation'] + my_dataRK['Sanitation_two'
```

In [265]:
```
my_dataRK['WASH_Safe_Score']= my_dataRK['Improved_sanitation'] + my_dataRK['Sanitation
```

In [266]:
```
my_dataRK['MV_WASH_Score']=0
my_dataRK['MV_WASH_Score']=(4-my_dataRK['WASH_Score'])/4
```

In [267]:
```
my_dataRK['MV_WASH_Safe_Score']=0
my_dataRK['MV_WASH_Safe_Score']=(4-my_dataRK['WASH_Safe_Score'])/4
```

In [268]:
```
my_dataRK['MV_WASH_Score'][my_dataRK.Roma==1].value_counts()
```

Out[268]:
```
1.00    25
0.75     5
0.50     5
0.25     2
Name: MV_WASH_Score, dtype: int64
```

In [269]:
```
my_dataRK['MV_WASH_Score'][my_dataRK.Roma==0].value_counts()
```

```
Out[269]: 1.00    40
          0.75    36
          0.50    18
          0.25     4
          Name: MV_WASH_Score, dtype: int64

In [270]: my_dataRK['Insecure_Housing'][my_dataRK.Roma==0].value_counts()

Out[270]: 0    71
          1    27
          Name: Insecure_Housing, dtype: int64

In [271]: my_dataRK['Insecure_Housing'][my_dataRK.Roma==1].value_counts()

Out[271]: 0    35
          1     2
          Name: Insecure_Housing, dtype: int64

In [272]: multivar_model = sm.formula.glm('MV_Roma~MV_Healthcare_Score + MV_WASH_Score + MV_Pove


          print my_data.keys()


          multivar_model.summary()

Index([u'Survey', u'Community', u'Household_size', u'Time_lived_there',
       u'Head_of_house', u'50+_men', u'50+_women', u'15-49_men',
       u'15-49_women', u'under_15_boys', u'under_15_girls',
       u'literate_men_(+15)', u'literate_women_(+15)', u'Religion', u'Gender',
       u'Age', u'Marital_status', u'Education', u'Occupation', u'Ethnicity',
       u'Drinking_water_sources_available', u'Primary_drinking_water_source',
       u'Primary_non-drinking_water_source',
       u'Who_uses_primary_non-drinking_water_source',
       u'Belief_in_safety_of_primary_drinking_water_source',
       u'Distance_of_primary_drinking_water_source',
       u'Length_of_time_to_collect_water', u'Pay_for_water',
       u'Water_treatment', u'Type_of_water_treatment',
       u'Satisfaction_level_with_water', u'Time_on_household_duties',
       u'Spouse_time_on_household_duties', u'Children_attend_school',
       u'Bathroom', u'Toilet_facility', u'Shared_toilet',
       u'Diarrhea_in_last_year', u'Diarrhea_number_of_people', u'Immunized',
       u'Children_immunized', u'Primary_care_physician', u'Medical_Insurance',
       u'Last_doctor_visit', u'Child_to_doctor', u'Land_for_personal_use',
       u'Animals_owned', u'weekly_expenses_(RON)', u'Property_documents',
       u'Items_owned', u'Electricity', u'Piped__tank_gas',
       u'Decider_for_female_earned_money', u'Conflict_between_Roma_and_non',
       u'Roma_segregated', u'Community_helpful', u'House', u'Roof', u'Floor',
       u'Housing_Score', u'Roma', u'Education_level', u'Family_size',
```

```
       u'Marital_status_simple', u'Employment_status', u'Total_men',
       u'Total_women', u'illiterate_men', u'illiterate_women',
       u'WeeklyExpense_perPerson', u'Expense_person', u'tap_water_availabe',
       u'tap_water_available', u'safe_water', u'Time_to_water',
       u'proper_toilet', u'Water_satisfaction', u'Employment_type',
       u'Geography', u'MV_Roma', u'Improved_sanitation', u'Sanitation_two',
       u'Improved_water', u'Modified_WASH_Score', u'Diarrhea',
       u'Healthcare_Score', u'MV_Healthcare_Score', u'Poverty_Score',
       u'MV_Poverty_Score', u'Insecure_Housing', u'WASH_Score',
       u'WASH_Safe_Score', u'MV_WASH_Score', u'MV_WASH_Safe_Score'],
      dtype='object')
```

Out[272]:

```
<class 'statsmodels.iolib.summary.Summary'>
"""
                 Generalized Linear Model Regression Results
==============================================================================
Dep. Variable:                MV_Roma   No. Observations:                  135
Model:                            GLM   Df Residuals:                      131
Model Family:                Gaussian   Df Model:                            3
Link Function:               identity   Scale:                  0.185557629887
Method:                          IRLS   Log-Likelihood:                -75.830
Date:                Tue, 14 Mar 2017   Deviance:                       24.308
Time:                        12:46:53   Pearson chi2:                     24.3
No. Iterations:                     4
========================================================================================
                          coef    std err          z      P>|z|      [95.0% Conf. Int.
----------------------------------------------------------------------------------------
Intercept               0.8828      0.143      6.193      0.000         0.603      1.16
MV_Healthcare_Score     0.3407      0.185      1.840      0.066        -0.022      0.70
MV_WASH_Score          -0.4104      0.172     -2.392      0.017        -0.747     -0.07
MV_Poverty_Score        0.3391      0.137      2.474      0.013         0.070      0.60
========================================================================================
"""
```

In [273]:

```
multivar_model = sm.formula.glm('MV_Roma~MV_Healthcare_Score + MV_WASH_Safe_Score + MV


print my_data.keys()


multivar_model.summary()
```

```
Index([u'Survey', u'Community', u'Household_size', u'Time_lived_there',
       u'Head_of_house', u'50+_men', u'50+_women', u'15-49_men',
       u'15-49_women', u'under_15_boys', u'under_15_girls',
       u'literate_men_(+15)', u'literate_women_(+15)', u'Religion', u'Gender',
       u'Age', u'Marital_status', u'Education', u'Occupation', u'Ethnicity',
```

```
       u'Drinking_water_sources_available', u'Primary_drinking_water_source',
       u'Primary_non-drinking_water_source',
       u'Who_uses_primary_non-drinking_water_source',
       u'Belief_in_safety_of_primary_drinking_water_source',
       u'Distance_of_primary_drinking_water_source',
       u'Length_of_time_to_collect_water', u'Pay_for_water',
       u'Water_treatment', u'Type_of_water_treatment',
       u'Satisfaction_level_with_water', u'Time_on_household_duties',
       u'Spouse_time_on_household_duties', u'Children_attend_school',
       u'Bathroom', u'Toilet_facility', u'Shared_toilet',
       u'Diarrhea_in_last_year', u'Diarrhea_number_of_people', u'Immunized',
       u'Children_immunized', u'Primary_care_physician', u'Medical_Insurance',
       u'Last_doctor_visit', u'Child_to_doctor', u'Land_for_personal_use',
       u'Animals_owned', u'weekly_expenses_(RON)', u'Property_documents',
       u'Items_owned', u'Electricity', u'Piped__tank_gas',
       u'Decider_for_female_earned_money', u'Conflict_between_Roma_and_non',
       u'Roma_segregated', u'Community_helpful', u'House', u'Roof', u'Floor',
       u'Housing_Score', u'Roma', u'Education_level', u'Family_size',
       u'Marital_status_simple', u'Employment_status', u'Total_men',
       u'Total_women', u'illiterate_men', u'illiterate_women',
       u'WeeklyExpense_perPerson', u'Expense_person', u'tap_water_availabe',
       u'tap_water_available', u'safe_water', u'Time_to_water',
       u'proper_toilet', u'Water_satisfaction', u'Employment_type',
       u'Geography', u'MV_Roma', u'Improved_sanitation', u'Sanitation_two',
       u'Improved_water', u'Modified_WASH_Score', u'Diarrhea',
       u'Healthcare_Score', u'MV_Healthcare_Score', u'Poverty_Score',
       u'MV_Poverty_Score', u'Insecure_Housing', u'WASH_Score',
       u'WASH_Safe_Score', u'MV_WASH_Score', u'MV_WASH_Safe_Score'],
      dtype='object')
```

Out[273]:
```
<class 'statsmodels.iolib.summary.Summary'>
"""
                 Generalized Linear Model Regression Results
==============================================================================
Dep. Variable:                MV_Roma   No. Observations:                  135
Model:                            GLM   Df Residuals:                      131
Model Family:                Gaussian   Df Model:                            3
Link Function:               identity   Scale:                  0.191296152658
Method:                          IRLS   Log-Likelihood:                -77.886
Date:                Tue, 14 Mar 2017   Deviance:                       25.060
Time:                        12:46:53   Pearson chi2:                     25.1
No. Iterations:                     4
==================================================================================
                          coef    std err          z      P>|z|      [95.0% Conf. Int.
----------------------------------------------------------------------------------
Intercept               0.7045      0.119      5.939      0.000       0.472      0.93
MV_Healthcare_Score     0.3277      0.189      1.736      0.083      -0.042      0.69
```

```
    MV_WASH_Safe_Score     -0.2050      0.161     -1.273      0.203         -0.521      0.11
    MV_Poverty_Score        0.3305      0.142      2.330      0.020          0.052      0.60
    ==========================================================================================
    """
```

```
In [274]: multivar_model = sm.formula.glm('MV_Roma~MV_WASH_Score + MV_Poverty_Score + Geography'


          print my_data.keys()


          multivar_model.summary()
```

```
Index([u'Survey', u'Community', u'Household_size', u'Time_lived_there',
       u'Head_of_house', u'50+_men', u'50+_women', u'15-49_men',
       u'15-49_women', u'under_15_boys', u'under_15_girls',
       u'literate_men_(+15)', u'literate_women_(+15)', u'Religion', u'Gender',
       u'Age', u'Marital_status', u'Education', u'Occupation', u'Ethnicity',
       u'Drinking_water_sources_available', u'Primary_drinking_water_source',
       u'Primary_non-drinking_water_source',
       u'Who_uses_primary_non-drinking_water_source',
       u'Belief_in_safety_of_primary_drinking_water_source',
       u'Distance_of_primary_drinking_water_source',
       u'Length_of_time_to_collect_water', u'Pay_for_water',
       u'Water_treatment', u'Type_of_water_treatment',
       u'Satisfaction_level_with_water', u'Time_on_household_duties',
       u'Spouse_time_on_household_duties', u'Children_attend_school',
       u'Bathroom', u'Toilet_facility', u'Shared_toilet',
       u'Diarrhea_in_last_year', u'Diarrhea_number_of_people', u'Immunized',
       u'Children_immunized', u'Primary_care_physician', u'Medical_Insurance',
       u'Last_doctor_visit', u'Child_to_doctor', u'Land_for_personal_use',
       u'Animals_owned', u'weekly_expenses_(RON)', u'Property_documents',
       u'Items_owned', u'Electricity', u'Piped__tank_gas',
       u'Decider_for_female_earned_money', u'Conflict_between_Roma_and_non',
       u'Roma_segregated', u'Community_helpful', u'House', u'Roof', u'Floor',
       u'Housing_Score', u'Roma', u'Education_level', u'Family_size',
       u'Marital_status_simple', u'Employment_status', u'Total_men',
       u'Total_women', u'illiterate_men', u'illiterate_women',
       u'WeeklyExpense_perPerson', u'Expense_person', u'tap_water_availabe',
       u'tap_water_available', u'safe_water', u'Time_to_water',
       u'proper_toilet', u'Water_satisfaction', u'Employment_type',
       u'Geography', u'MV_Roma', u'Improved_sanitation', u'Sanitation_two',
       u'Improved_water', u'Modified_WASH_Score', u'Diarrhea',
       u'Healthcare_Score', u'MV_Healthcare_Score', u'Poverty_Score',
       u'MV_Poverty_Score', u'Insecure_Housing', u'WASH_Score',
       u'WASH_Safe_Score', u'MV_WASH_Score', u'MV_WASH_Safe_Score'],
      dtype='object')
```

```
Out[274]: <class 'statsmodels.iolib.summary.Summary'>
"""
                 Generalized Linear Model Regression Results
==============================================================================
Dep. Variable:                MV_Roma   No. Observations:                  135
Model:                            GLM   Df Residuals:                      131
Model Family:                Gaussian   Df Model:                            3
Link Function:               identity   Scale:                  0.189428204616
Method:                          IRLS   Log-Likelihood:                -77.224
Date:                Tue, 14 Mar 2017   Deviance:                       24.815
Time:                        12:46:53   Pearson chi2:                     24.8
No. Iterations:                     4
====================================================================================
                       coef    std err          z      P>|z|      [95.0% Conf. Int.]
------------------------------------------------------------------------------------
Intercept            1.0302      0.200      5.158      0.000        0.639       1.422
MV_WASH_Score       -0.3927      0.173     -2.264      0.024       -0.733      -0.053
MV_Poverty_Score     0.4037      0.145      2.784      0.005        0.120       0.688
Geography           -0.0667      0.083     -0.800      0.424       -0.230       0.097
====================================================================================
"""
```

```
In [275]: my_dataRK.groupby(['Community','Roma'])['Household_size'].mean()
```

```
Out[275]: Community  Roma
          1          0       4.714286
                     1       5.000000
          2          0       5.875000
                     1       4.833333
          3          0       5.333333
                     1       4.166667
          4          0       6.076923
                     1       2.941176
          5          0       4.733333
                     1       4.000000
          Name: Household_size, dtype: float64
```

```
In [276]: my_dataRK.groupby(['Community','Roma'])['Gender'].value_counts()
```

```
Out[276]: Community  Roma  Gender
          1          0     2          20
                           1           8
                     1     1           1
          2          0     1          18
                           2           6
                     1     1           3
                           2           3
          3          0     2          10
                           1           8
```

```
           1     1          7
                 2          5
4          0     2          7
                 1          6
           1     2         12
                 1          5
5          0     1         10
                 2          5
           1     1          1
dtype: int64
```

```
In [277]: my_dataRK.groupby('Roma')['Gender'].value_counts()
```

```
Out[277]: Roma  Gender
          0     1         50
                2         48
          1     2         20
                1         17
          dtype: int64
```

```
In [278]: my_dataRK.groupby(['Community','Roma'])['Age'].mean()
```

```
Out[278]: Community  Roma
          1          0       49.321429
                     1       52.000000
          2          0       48.083333
                     1       36.833333
          3          0       42.611111
                     1       50.083333
          4          0       42.692308
                     1       60.352941
          5          0       35.400000
                     1       40.000000
          Name: Age, dtype: float64
```

```
In [279]: my_dataRK.groupby('Community')['Age'].mean()
```

```
Out[279]: Community
          1    49.413793
          2    45.833333
          3    45.600000
          4    52.700000
          5    35.687500
          Name: Age, dtype: float64
```

```
In [280]: my_dataRK.groupby('Roma')['Age'].mean()
```

```
Out[280]: Roma
          0    44.775510
          1    52.432432
          Name: Age, dtype: float64
```

```
In [281]: my_dataRK['Age'].mean()
```

```
Out[281]: 46.874074074074073
```

```
In [282]: my_dataRK.groupby(['Community','Roma'])['Education'].mean()
```

```
Out[282]: Community  Roma
          1          0       3.928571
                     1       8.000000
          2          0       3.750000
                     1       8.000000
          3          0       3.888889
                     1       6.833333
          4          0       3.615385
                     1       4.764706
          5          0       6.133333
                     1       5.000000
          Name: Education, dtype: float64
```

```
In [283]: my_dataRK.groupby('Roma')['Education'].mean()
```

```
Out[283]: Roma
          0    4.173469
          1    6.054054
          Name: Education, dtype: float64
```

```
In [284]: my_dataRK['Education'].mean()
```

```
Out[284]: 4.6888888888888891
```

```
In [285]: my_dataRK.groupby(['Community','Roma'])['Marital_status_simple'].value_counts()
```

```
Out[285]: Community  Roma  Marital_status_simple
          1          0     0                        20
                           1                         8
                     1     0                         1
          2          0     0                        21
                           1                         3
                     1     0                         5
                           1                         1
          3          0     0                        17
                           1                         1
                     1     0                        11
                           1                         1
          4          0     0                        11
                           1                         2
                     1     0                        10
                           1                         7
          5          0     0                        11
                           1                         4
                     1     0                         1
          dtype: int64
```

```
In [286]: my_dataRK.groupby(['Community','Roma'])['Employment_status'].value_counts()

Out[286]: Community  Roma  Employment_status
          1          0     1                    25
                           0                     3
                     1     1                     1
          2          0     1                    20
                           0                     4
                     1     0                     4
                           1                     2
          3          0     1                    13
                           0                     5
                     1     1                     9
                           0                     3
          4          0     1                    10
                           0                     3
                     1     1                    13
                           0                     4
          5          0     0                    11
                           1                     4
                     1     0                     1
          dtype: int64

In [287]: my_dataRK['Employment_status'].value_counts()

Out[287]: 1    97
          0    38
          Name: Employment_status, dtype: int64

In [288]: my_dataRK.groupby(['Community','Roma'])['illiterate_men'].value_counts()

Out[288]: Community  Roma  illiterate_men
          1          0     0.0               17
                           1.0                6
                           2.0                5
                     1     1.0                1
          2          0     0.0               10
                           1.0               10
                           2.0                3
                     1     0.0                6
          3          0     0.0                9
                           1.0                5
                           2.0                3
                           3.0                1
                     1     0.0               12
          4          0     0.0                5
                           1.0                4
                           2.0                4
                     1     0.0               16
```

```
                      1.0               1
5          0          0.0              14
                      1.0               1
           1          0.0               1
dtype: int64
```

In [289]: my_dataRK.groupby(['Community','Roma'])['illiterate_women'].value_counts()

```
Out[289]: Community  Roma  illiterate_women
1          0     0.0                 16
                 1.0                  8
                 2.0                  3
                 3.0                  1
           1     0.0                  1
2          0     1.0                 10
                 0.0                  7
                 2.0                  4
                 3.0                  1
           1     0.0                  6
3          0     0.0                  9
                 1.0                  4
                 2.0                  3
                 3.0                  2
           1     0.0                 12
4          0     1.0                  7
                 0.0                  4
                 3.0                  1
                 4.0                  1
           1     0.0                 16
                 1.0                  1
5          0     0.0                 13
                 1.0                  2
           1     0.0                  1
dtype: int64
```

In [290]: my_dataRK.groupby(['Community','Roma'])['Total_men'].value_counts()

```
Out[290]: Community  Roma  Total_men
1          0     1            12
                 2             8
                 3             5
                 0             3
           1     4             1
2          0     2            11
                 1            10
                 4             2
                 3             1
           1     2             4
                 1             2
```

```
3          0     1            8
                 2            8
                 3            2
           1     2            6
                 1            4
                 3            2
4          0     1            6
                 2            4
                 3            2
                 4            1
           1     1            7
                 0            5
                 2            4
                 4            1
5          0     1            6
                 2            6
                 5            2
                 3            1
           1     2            1
dtype: int64
```

In [291]:
```
my_dataRK.groupby(['Community','Roma'])['Total_women'].value_counts()
```

Out[291]:
```
Community  Roma  Total_women
1          0     1               12
                 2                7
                 3                6
                 0                2
                 5                1
           1     1                1
2          0     2               10
                 1                8
                 3                5
                 4                1
           1     2                3
                 1                2
                 3                1
3          0     2                6
                 1                5
                 3                4
                 4                2
                 5                1
           1     1                6
                 2                2
                 4                2
                 0                1
                 3                1
4          0     1                7
```

```
                    4              3
                    2              2
                    3              1
              1     1             12
                    2              2
                    3              2
                    0              1
5             0     2              8
                    1              5
                    3              1
                    4              1
              1     1              1
dtype: int64
```

In [292]: `my_dataRK.groupby('Roma')['Time_to_water'].describe()`

Out[292]:
```
Roma
0      count    98.000000
       mean      1.122449
       std       0.911160
       min       0.000000
       25%       1.000000
       50%       1.000000
       75%       1.000000
       max       4.000000
1      count    37.000000
       mean      1.000000
       std       0.527046
       min       0.000000
       25%       1.000000
       50%       1.000000
       75%       1.000000
       max       2.000000
dtype: float64
```

In [293]: `my_dataRK.groupby('Geography')['Time_to_water'].describe()`

Out[293]:
```
Geography
1           count    46.000000
            mean      1.217391
            std       0.986870
            min       0.000000
            25%       1.000000
            50%       1.000000
            75%       1.000000
            max       4.000000
2           count    89.000000
            mean      1.022472
            std       0.722646
```

```
    min      0.000000
    25%      1.000000
    50%      1.000000
    75%      1.000000
    max      4.000000
dtype: float64
```

```
In [294]: my_dataRK.groupby('Roma')['Improved_sanitation'].value_counts()

Out[294]: Roma  Improved_sanitation
          0     0                      81
                1                      17
          1     0                      29
                1                       8
          dtype: int64

In [295]: stats.ttest_ind(my_dataRK[my_dataRK.Roma==1]['Improved_sanitation'],my_dataRK[my_dataR

Out[295]: Ttest_indResult(statistic=0.56676247803505464, pvalue=0.57183080287152677)

In [296]: my_dataRK.groupby('Roma')['Sanitation_two'].value_counts()

Out[296]: Roma  Sanitation_two
          0     0                 78
                1                 20
          1     0                 29
                1                  8
          dtype: int64

In [297]: stats.ttest_ind(my_dataRK[my_dataRK.Roma==1]['Sanitation_two'],my_dataRK[my_dataRK.Rom

Out[297]: Ttest_indResult(statistic=0.15396868271346401, pvalue=0.87786781182491347)

In [298]: stats.ttest_ind(my_dataRK[my_dataRK.Roma==1]['Insecure_Housing'],my_dataRK[my_dataRK.R

Out[298]: Ttest_indResult(statistic=-2.8576917908457786, pvalue=0.004954673247635261)

In [299]: stats.ttest_ind(my_dataRK[my_dataRK.Roma==1]['Improved_water'],my_dataRK[my_dataRK.Rom

Out[299]: Ttest_indResult(statistic=-1.7011716899468075, pvalue=0.091247536407028096)

In [300]: my_dataRK.groupby('Roma')['Improved_water'].value_counts()

Out[300]: Roma  Improved_water
          0     0                 78
                1                 20
          1     0                 34
                1                  3
          dtype: int64
```

```
In [301]: table_18=epipy.create_2x2(my_data, row='Roma', column='Improved_sanitation', row_order
                            col_order=[0,1]) #rows are 0=roma, 1=not and columns are 0=not improv

In [302]: table_18

Out[302]:        0   1  All
          0     81  17   98
          1     29   8   37
          All  110  25  135

In [303]: epipy.analyze_2x2(table_18)

Odds ratio: 1.31 (95% CI: (0.51, 3.37))
Relative risk: 1.05 (95% CI: (0.87, 1.28))

Chi square: 0.325264503836
p value: 0.98812543102


In [304]: table_19=epipy.create_2x2(my_data, row='Roma', column='Improved_water', row_order=[0,1
                            col_order=[0,1]) #rows are 0=roma, 1=not and columns are 0=not improv

In [305]: table_19

Out[305]:        0   1  All
          0     78  20   98
          1     34   3   37
          All  112  23  135

In [306]: epipy.analyze_2x2(table_19)

Odds ratio: 0.34 (95% CI: (0.1, 1.24))
Relative risk: 0.87 (95% CI: (0.75, 0.99))

Chi square: 2.87494689835
p value: 0.578966061994


In [307]: table_20=epipy.create_2x2(my_data, row='Roma', column='Sanitation_two', row_order=[0,1
                            col_order=[0,1]) #rows are 0=roma, 1=not and columns are 0=not improv

In [308]: table_20

Out[308]:        0   1  All
          0     78  20   98
          1     29   8   37
          All  107  28  135

In [309]: epipy.analyze_2x2(table_20)
```

```
Odds ratio: 1.08 (95% CI: (0.43, 2.71))
Relative risk: 1.02 (95% CI: (0.83, 1.24))

Chi square: 0.0240585535264
p value: 0.999928225867
```

In [310]: my_dataRK['Distance_of_primary_drinking_water_source']

```
Out[310]: 0      1
          1      3
          2      1
          3      1
          4      1
          5      1
          6      1
          7      1
          8      1
          9      1
          10     1
          11     1
          12     1
          13     1
          14     1
          15     1
          16     1
          17     1
          18     1
          19     1
          20     1
          21     1
          22     1
          23     1
          24     1
          25     1
          26     1
          27     1
          28     1
          29     1
                ..
          105    2
          106    2
          107    2
          108    1
          109    2
          110    1
          111    1
          112    1
```

```
113    1
114    1
115    1
116    2
117    2
118    1
119    1
120    1
121    1
122    1
123    1
124    1
125    1
126    1
127    1
128    1
129    1
130    1
131    1
132    1
133    1
134    1
Name: Distance_of_primary_drinking_water_source, dtype: int64
```

```
In [311]: my_dataRK['Water_distance']=0
          my_dataRK.loc[(my_dataRK['Distance_of_primary_drinking_water_source'])==1,'Water_dista
          my_dataRK.loc[(my_dataRK['Distance_of_primary_drinking_water_source'])==3,'Water_dista
          my_dataRK.loc[(my_dataRK['Distance_of_primary_drinking_water_source'])==2,'Water_dista
```

```
In [312]: table_21=epipy.create_2x2(my_data, row='Roma', column='Water_distance', row_order=[0,1
                            col_order=[1,0]) #rows are 0=roma, 1=not and columns are 0=less than
```

```
In [313]: table_21
```

```
Out[313]:        1    0  All
          0     12   86   98
          1      4   33   37
          All   16  119  135
```

```
In [314]: epipy.analyze_2x2(table_21)
```

```
Odds ratio: 1.15 (95% CI: (0.35, 3.82))
Relative risk: 1.13 (95% CI: (0.39, 3.29))

Chi square: 0.0528744316259
p value: 0.999656635398
```

```
In [315]: table_22=epipy.create_2x2(my_data, row='Roma', column='Insecure_Housing', row_order=[0
                            col_order=[0,1]) #rows are 0=roma, 1=not and columns are 0=yes and 1=
```

In [316]: table_22

Out[316]:

	0	1	All
0	71	27	98
1	35	2	37
All	106	29	135

In [317]: epipy.analyze_2x2(table_22)

```
Odds ratio: 0.15 (95% CI: (0.03, 0.67))
Relative risk: 0.77 (95% CI: (0.66, 0.88))

Chi square: 7.80967922698
p value: 0.0988039969998
```

In [318]:
```
table_23=epipy.create_2x2(my_data, row='Geography', column='Time_to_water', row_order=
                          col_order=[0,1]) #rows are 0=roma, 1=not and columns are 0=yes and 1=
```

In [319]: table_23

Out[319]:

	0	1	All
0	NaN	NaN	NaN
1	6.0	32.0	46.0
All	19.0	99.0	135.0

In [320]: my_dataRK['Time_to_water']

Out[320]:
```
0      0
1      0
2      1
3      1
4      1
5      1
6      1
7      1
8      1
9      1
10     1
11     1
12     2
13     1
14     1
15     1
16     1
17     4
18     1
19     1
20     1
```

```
21     1
22     1
23     1
24     1
25     0
26     0
27     1
28     1
29     1
      ..
105    2
106    4
107    4
108    1
109    1
110    1
111    2
112    1
113    1
114    1
115    1
116    2
117    1
118    1
119    0
120    0
121    0
122    1
123    0
124    1
125    1
126    1
127    1
128    1
129    1
130    0
131    1
132    0
133    1
134    1
Name: Time_to_water, dtype: int64
```

```
In [321]: my_dataRK['Water_Time']=0
          my_dataRK.loc[(my_dataRK['Time_to_water']==0), 'Water_Time']=0
          my_dataRK.loc[(my_dataRK['Time_to_water']==1), 'Water_Time']=0
          my_dataRK.loc[(my_dataRK['Time_to_water']==2), 'Water_Time']=1
          my_dataRK.loc[(my_dataRK['Time_to_water']==3), 'Water_Time']=1
          my_dataRK.loc[(my_dataRK['Time_to_water']==4), 'Water_Time']=1
```

```
In [322]: table_24=epipy.create_2x2(my_dataRK, row='Geography', column='Water_Time', row_order=[
                           col_order=[0,1])

In [323]: table_24

Out[323]:          0     1    All
          0      NaN   NaN    NaN
          1     38.0   8.0   46.0
          All  118.0  17.0  135.0

In [324]: my_dataRK['Geography']

Out[324]: 0      2
          1      2
          2      2
          3      2
          4      2
          5      2
          6      2
          7      2
          8      2
          9      2
          10     2
          11     2
          12     2
          13     2
          14     2
          15     2
          16     2
          17     2
          18     2
          19     2
          20     2
          21     2
          22     2
          23     2
          24     2
          25     2
          26     2
          27     2
          28     2
          29     2
                ..
          105    2
          106    2
          107    2
          108    2
          109    2
          110    2
```

```
111    2
112    2
113    2
114    2
115    2
116    2
117    2
118    2
119    1
120    1
121    1
122    1
123    1
124    1
125    1
126    1
127    1
128    1
129    1
130    1
131    1
132    1
133    1
134    1
Name: Geography, dtype: int64
```

```
In [325]: my_dataRK['location']=0

In [326]: my_dataRK.loc[(my_dataRK['Geography']==1), 'location']=0
          my_dataRK.loc[(my_dataRK['Geography']==2), 'location']=1

In [327]: table_25=epipy.create_2x2(my_dataRK, row='location', column='Water_Time', row_order=[1
                            col_order=[1,0]) ##0=urban, 1=rural, 0=under 15m, 1=more than 15m

In [328]: table_25

Out[328]:       1    0  All
          1     9   80   89
          0     8   38   46
          All  17  118  135

In [329]: epipy.analyze_2x2(table_25)

Odds ratio: 0.53 (95% CI: (0.19, 1.49))
Relative risk: 0.58 (95% CI: (0.24, 1.41))

Chi square: 1.45978040476
p value: 0.833741178168
```

```
In [330]: stats.ttest_ind(my_dataRK[my_dataRK.Roma==1]['Diarrhea_in_last_year'],my_dataRK[my_dat

Out[330]: Ttest_indResult(statistic=-1.8400733021653279, pvalue=0.067986771966201037)

In [331]: my_dataRK['Diarrhea_in_last_year'][my_dataRK.Roma==0].var()

Out[331]: 0.24584472964443513

In [332]: my_dataRK['Diarrhea_in_last_year'][my_dataRK.Roma==1].var()

Out[332]: 0.24774774774774772

In [333]: stats.ttest_ind(my_dataRK[my_dataRK.Roma==1]['Immunized'],my_dataRK[my_dataRK.Roma==0]

Out[333]: Ttest_indResult(statistic=0.67829216020886163, pvalue=0.4987649129452133)

In [334]: my_dataRK['Immunized'][my_dataRK.Roma==0].var()

Out[334]: 0.10856301283399962

In [335]: my_dataRK['Immunized'][my_dataRK.Roma==1].var()

Out[335]: 0.076576576576576585

In [336]: stats.ttest_ind(my_dataRK[my_dataRK.Roma==1]['Medical_Insurance'],my_dataRK[my_dataRK.

Out[336]: Ttest_indResult(statistic=1.0568857149980391, pvalue=0.29247967043309459)

In [337]: my_dataRK['Medical_Insurance'][my_dataRK.Roma==1].var()

Out[337]: 0.099099099099099128

In [338]: my_dataRK['Medical_Insurance'][my_dataRK.Roma==0].var()

Out[338]: 0.15148327372185985

In [339]: stats.ttest_ind(my_dataRK[my_dataRK.Roma==1]['Primary_care_physician'],my_dataRK[my_da

Out[339]: Ttest_indResult(statistic=-0.2310269762543104, pvalue=0.81764886681349147)

In [340]: table_26=epipy.create_2x2(my_dataRK, row='Roma', column='Primary_care_physician', row_
                           col_order=[0,1]) ##0=urban, 1=rural, 0=under 15m, 1=more than 15m

In [341]: table_26

Out[341]:      0    1  All
          0    2   96   98
          1    1   36   37
          All  3  132  135

In [342]: epipy.analyze_2x2(table_26)
```

```
Odds ratio: 0.75 (95% CI: (0.07, 8.53))
Relative risk: 0.76 (95% CI: (0.07, 8.08))

Chi square: 0.0541543398686
p value: 0.999639964142
```

```
In [343]: my_dataRK['Electricity']
```

```
Out[343]: 0      1
          1      1
          2      1
          3      0
          4      1
          5      1
          6      1
          7      0
          8      0
          9      0
          10     1
          11     1
          12     1
          13     1
          14     1
          15     1
          16     1
          17     1
          18     1
          19     1
          20     1
          21     1
          22     1
          23     1
          24     1
          25     1
          26     1
          27     1
          28     1
          29     1
                ..
          105    1
          106    0
          107    1
          108    1
          109    1
          110    1
          111    1
          112    1
```

```
113    1
114    1
115    1
116    1
117    1
118    1
119    1
120    1
121    1
122    1
123    1
124    1
125    1
126    1
127    0
128    1
129    1
130    1
131    1
132    1
133    0
134    1
Name: Electricity, dtype: int64
```

```
In [344]: my_dataRK.groupby('Roma')['Electricity'].value_counts()
```

```
Out[344]: Roma  Electricity
          0     1              85
                0              13
          1     1              36
                0               1
          dtype: int64
```

```
In [345]: stats.ttest_ind(my_dataRK[my_dataRK.Roma==1]['Electricity'],my_dataRK[my_dataRK.Roma==
```

```
Out[345]: Ttest_indResult(statistic=1.8038592813170677, pvalue=0.073516659815817786)
```

```
In [346]: table_27=epipy.create_2x2(my_dataRK, row='Roma', column='Electricity', row_order=[0,1]
                                 col_order=[0,1])
```

```
In [347]: table_27
```

```
Out[347]:       0    1  All
          0    13   85   98
          1     1   36   37
          All  14  121  135
```

```
In [348]: epipy.analyze_2x2(table_27)
```

```
Odds ratio: 5.51 (95% CI: (0.69, 43.68))
Relative risk: 4.91 (95% CI: (0.67, 36.21))

Chi square: 3.22396345819
p value: 0.521069154072
```

```
In [349]: my_dataRK.groupby('Roma')['Piped__tank_gas'].value_counts()

Out[349]: Roma  Piped__tank_gas
          0     1                  66
                0                  32
          1     1                  30
                0                   7
          dtype: int64

In [350]: stats.ttest_ind(my_dataRK[my_dataRK.Roma==1]['Piped__tank_gas'],my_dataRK[my_dataRK.Ro

Out[350]: Ttest_indResult(statistic=1.5731745330279541, pvalue=0.11805429179434981)

In [351]: table_28=epipy.create_2x2(my_dataRK, row='Roma', column='Piped__tank_gas', row_order=[
                              col_order=[0,1])

In [352]: epipy.analyze_2x2(table_28)
```

```
Odds ratio: 2.08 (95% CI: (0.82, 5.24))
Relative risk: 1.73 (95% CI: (0.84, 3.56))

Chi square: 2.46620295727
p value: 0.650697729483
```

```
In [353]: my_dataRK.groupby('Roma')['Expense_person'].value_counts()

Out[353]: Roma  Expense_person
          0     0                 54
                1                 44
          1     1                 21
                0                 16
          dtype: int64

In [354]: my_dataRK.groupby('location')['Expense_person'].value_counts()

Out[354]: location  Expense_person
          0         1                 31
                    0                 15
          1         0                 55
                    1                 34
          dtype: int64
```

```
In [355]: stats.ttest_ind(my_dataRK[my_dataRK.Roma==1]['Expense_person'],my_dataRK[my_dataRK.Rom

Out[355]: Ttest_indResult(statistic=1.2277841809028889, pvalue=0.22169652389444819)

In [356]: stats.ttest_ind(my_dataRK[my_dataRK.location==1]['Expense_person'],my_dataRK[my_dataRK

Out[356]: Ttest_indResult(statistic=-3.323024975681669, pvalue=0.0011503485619188845)

In [357]: table_29=epipy.create_2x2(my_dataRK, row='Roma', column='Expense_person', row_order=[0
                                 col_order=[0,1])

In [358]: epipy.analyze_2x2(table_29)

Odds ratio: 1.61 (95% CI: (0.75, 3.45))
Relative risk: 1.27 (95% CI: (0.85, 1.92))

Chi square: 1.51297406399
p value: 0.824340816635


In [359]: table_30=epipy.create_2x2(my_dataRK, row='location', column='Expense_person', row_orde
                                 col_order=[1,0])

In [360]: epipy.analyze_2x2(table_30)

Odds ratio: 3.34 (95% CI: (1.58, 7.08))
Relative risk: 1.76 (95% CI: (1.27, 2.46))

Chi square: 10.3492849359
p value: 0.0349376062464


In [361]: multivar_model = sm.formula.glm('Roma~MV_Healthcare_Score + MV_WASH_Score + Geography


          print my_data.keys()


          multivar_model.summary()

Index([u'Survey', u'Community', u'Household_size', u'Time_lived_there',
       u'Head_of_house', u'50+_men', u'50+_women', u'15-49_men',
       u'15-49_women', u'under_15_boys', u'under_15_girls',
       u'literate_men_(+15)', u'literate_women_(+15)', u'Religion', u'Gender',
       u'Age', u'Marital_status', u'Education', u'Occupation', u'Ethnicity',
       u'Drinking_water_sources_available', u'Primary_drinking_water_source',
       u'Primary_non-drinking_water_source',
       u'Who_uses_primary_non-drinking_water_source',
       u'Belief_in_safety_of_primary_drinking_water_source',
       u'Distance_of_primary_drinking_water_source',
```

```
 u'Length_of_time_to_collect_water', u'Pay_for_water',
 u'Water_treatment', u'Type_of_water_treatment',
 u'Satisfaction_level_with_water', u'Time_on_household_duties',
 u'Spouse_time_on_household_duties', u'Children_attend_school',
 u'Bathroom', u'Toilet_facility', u'Shared_toilet',
 u'Diarrhea_in_last_year', u'Diarrhea_number_of_people', u'Immunized',
 u'Children_immunized', u'Primary_care_physician', u'Medical_Insurance',
 u'Last_doctor_visit', u'Child_to_doctor', u'Land_for_personal_use',
 u'Animals_owned', u'weekly_expenses_(RON)', u'Property_documents',
 u'Items_owned', u'Electricity', u'Piped__tank_gas',
 u'Decider_for_female_earned_money', u'Conflict_between_Roma_and_non',
 u'Roma_segregated', u'Community_helpful', u'House', u'Roof', u'Floor',
 u'Housing_Score', u'Roma', u'Education_level', u'Family_size',
 u'Marital_status_simple', u'Employment_status', u'Total_men',
 u'Total_women', u'illiterate_men', u'illiterate_women',
 u'WeeklyExpense_perPerson', u'Expense_person', u'tap_water_availabe',
 u'tap_water_available', u'safe_water', u'Time_to_water',
 u'proper_toilet', u'Water_satisfaction', u'Employment_type',
 u'Geography', u'MV_Roma', u'Improved_sanitation', u'Sanitation_two',
 u'Improved_water', u'Modified_WASH_Score', u'Diarrhea',
 u'Healthcare_Score', u'MV_Healthcare_Score', u'Poverty_Score',
 u'MV_Poverty_Score', u'Insecure_Housing', u'WASH_Score',
 u'WASH_Safe_Score', u'MV_WASH_Score', u'MV_WASH_Safe_Score',
 u'Water_distance', u'Water_Time', u'location'],
dtype='object')
```

Out[361]:

```
<class 'statsmodels.iolib.summary.Summary'>
"""
                 Generalized Linear Model Regression Results
==============================================================================
Dep. Variable:                   Roma   No. Observations:                  135
Model:                            GLM   Df Residuals:                      130
Model Family:                Gaussian   Df Model:                            4
Link Function:               identity   Scale:                  0.186164544209
Method:                          IRLS   Log-Likelihood:                -75.533
Date:                Tue, 14 Mar 2017   Deviance:                       24.201
Time:                        12:46:54   Pearson chi2:                     24.2
No. Iterations:                     4
=================================================================================
                          coef    std err          z      P>|z|      [95.0% Conf. Int.
---------------------------------------------------------------------------------
Intercept               0.0119      0.199      0.059      0.953        -0.379     0.40
MV_Healthcare_Score    -0.3369      0.186     -1.816      0.069        -0.701     0.02
MV_WASH_Score           0.4242      0.173      2.455      0.014         0.086     0.76
Geography               0.0626      0.083      0.757      0.449        -0.099     0.22
MV_Poverty_Score       -0.3736      0.145     -2.582      0.010        -0.657    -0.09
=================================================================================
```

```
"""
```

In [362]:
```
multivar_model = sm.formula.glm('Roma~MV_Healthcare_Score + MV_WASH_Score + Geography'

print my_data.keys()

multivar_model.summary()
```

```
Index([u'Survey', u'Community', u'Household_size', u'Time_lived_there',
       u'Head_of_house', u'50+_men', u'50+_women', u'15-49_men',
       u'15-49_women', u'under_15_boys', u'under_15_girls',
       u'literate_men_(+15)', u'literate_women_(+15)', u'Religion', u'Gender',
       u'Age', u'Marital_status', u'Education', u'Occupation', u'Ethnicity',
       u'Drinking_water_sources_available', u'Primary_drinking_water_source',
       u'Primary_non-drinking_water_source',
       u'Who_uses_primary_non-drinking_water_source',
       u'Belief_in_safety_of_primary_drinking_water_source',
       u'Distance_of_primary_drinking_water_source',
       u'Length_of_time_to_collect_water', u'Pay_for_water',
       u'Water_treatment', u'Type_of_water_treatment',
       u'Satisfaction_level_with_water', u'Time_on_household_duties',
       u'Spouse_time_on_household_duties', u'Children_attend_school',
       u'Bathroom', u'Toilet_facility', u'Shared_toilet',
       u'Diarrhea_in_last_year', u'Diarrhea_number_of_people', u'Immunized',
       u'Children_immunized', u'Primary_care_physician', u'Medical_Insurance',
       u'Last_doctor_visit', u'Child_to_doctor', u'Land_for_personal_use',
       u'Animals_owned', u'weekly_expenses_(RON)', u'Property_documents',
       u'Items_owned', u'Electricity', u'Piped__tank_gas',
       u'Decider_for_female_earned_money', u'Conflict_between_Roma_and_non',
       u'Roma_segregated', u'Community_helpful', u'House', u'Roof', u'Floor',
       u'Housing_Score', u'Roma', u'Education_level', u'Family_size',
       u'Marital_status_simple', u'Employment_status', u'Total_men',
       u'Total_women', u'illiterate_men', u'illiterate_women',
       u'WeeklyExpense_perPerson', u'Expense_person', u'tap_water_availabe',
       u'tap_water_available', u'safe_water', u'Time_to_water',
       u'proper_toilet', u'Water_satisfaction', u'Employment_type',
       u'Geography', u'MV_Roma', u'Improved_sanitation', u'Sanitation_two',
       u'Improved_water', u'Modified_WASH_Score', u'Diarrhea',
       u'Healthcare_Score', u'MV_Healthcare_Score', u'Poverty_Score',
       u'MV_Poverty_Score', u'Insecure_Housing', u'WASH_Score',
       u'WASH_Safe_Score', u'MV_WASH_Score', u'MV_WASH_Safe_Score',
       u'Water_distance', u'Water_Time', u'location'],
      dtype='object')
```

Out[362]:
```
<class 'statsmodels.iolib.summary.Summary'>
"""
```

```
                 Generalized Linear Model Regression Results
==============================================================================
Dep. Variable:                   Roma   No. Observations:                  135
Model:                            GLM   Df Residuals:                      131
Model Family:                Gaussian   Df Model:                            3
Link Function:               identity   Scale:                  0.194219178365
Method:                          IRLS   Log-Likelihood:                -78.910
Date:                Tue, 14 Mar 2017   Deviance:                       25.443
Time:                        12:46:54   Pearson chi2:                     25.4
No. Iterations:                     4
==============================================================================
                         coef    std err          z      P>|z|      [95.0% Conf. Int.
------------------------------------------------------------------------------
Intercept              0.0892      0.201      0.443      0.658        -0.305     0.48
MV_Healthcare_Score   -0.3918      0.188     -2.081      0.037        -0.761    -0.02
MV_WASH_Score          0.3399      0.173      1.961      0.050         0.000     0.68
Geography             -0.0046      0.080     -0.058      0.954        -0.162     0.15
==============================================================================
"""
```

In [363]:
```
my_dataRK.head
```

Out[363]:
```
<bound method DataFrame.head of      Survey  Community  Household_size  Time_lived_the
0         1          1               8            51.0                1
1         2          1               4            15.0                1
2         3          1               4             4.0                1
3         4          1               1            11.0                1
4         5          1               9            12.0                1
5         6          1              12            40.0                2
6         7          1               7            35.0                2
7         8          1               4             4.0                1
8         9          1               4             7.0                1
9        10          1               1            30.0                2
10       11          1               2            33.0                1
11       12          1               2            45.0                1
12       13          1               8            12.0                2
13       14          1               3            25.0                1
14       15          1               6            30.0                2
15       16          1               8            32.0                2
16       17          1               1            50.0                2
17       18          1               6            60.0                1
18       19          1               5            30.0                2
19       20          1               2            50.0                2
20       21          1               7            40.0                1
21       22          1               3            10.0                1
22       23          1               2            35.0                1
23       24          1               8            35.0                1
24       25          1               1            40.0                2
```

25	26	1	7	26.0	3
26	27	1	5	40.0	1
27	28	1	3	37.0	2
28	29	1	4	30.0	2
29	30	2	10	15.0	1
..	...	...	...	...	...
105	106	4	4	20.0	2
106	107	4	6	40.0	1
107	108	4	9	41.0	1
108	109	4	8	18.0	1
109	110	4	4	18.0	2
110	111	4	6	8.0	2
111	112	4	3	NaN	1
112	113	4	1	30.0	2
113	114	4	5	10.0	1
114	115	4	4	22.0	2
115	116	4	1	55.0	2
116	117	4	5	52.0	2
117	118	4	2	70.0	2
118	119	4	1	45.0	2
119	120	5	5	40.0	1
120	121	5	4	3.0	1
121	122	5	3	23.0	1
122	123	5	7	40.0	1
123	124	5	4	14.0	1
124	125	5	9	20.0	1
125	126	5	4	13.0	1
126	127	5	2	15.0	1
127	128	5	9	10.0	1
128	129	5	3	30.0	1
129	130	5	4	20.0	1
130	131	5	3	45.0	1
131	132	5	5	45.0	1
132	133	5	4	50.0	1
133	134	5	5	30.0	1
134	135	5	4	12.0	1

	50+_men	50+_women	15-49_men	15-49_women	under_15_boys	...	\
0	1	1	2	2	1	...	
1	0	0	2	2	0	...	
2	0	0	1	1	0	...	
3	1	0	0	0	0	...	
4	1	1	2	2	1	...	
5	1	1	1	4	2	...	
6	0	1	3	1	2	...	
7	0	0	1	1	0	...	
8	0	0	1	1	1	...	
9	0	1	0	0	0	...	

10	1	1	0	0	0	...
11	1	1	0	0	0	...
12	1	1	1	1	2	...
13	0	0	1	2	0	...
14	0	1	2	2	0	...
15	0	1	1	2	1	...
16	0	1	0	0	0	...
17	1	1	2	2	0	...
18	1	1	1	0	2	...
19	0	1	1	0	0	...
20	1	1	1	1	3	...
21	0	0	1	1	0	...
22	1	0	0	0	1	...
23	0	1	2	2	1	...
24	0	1	0	0	0	...
25	1	0	2	2	1	...
26	1	0	3	1	0	...
27	1	1	1	0	0	...
28	0	1	1	1	1	...
29	1	1	3	3	1	...
..	...	...	...	...	...	...
105	0	1	2	0	1	...
106	0	0	3	2	0	...
107	1	1	3	3	1	...
108	0	0	1	4	1	...
109	0	0	2	1	0	...
110	0	0	2	1	3	...
111	0	0	1	1	0	...
112	0	1	0	0	0	...
113	0	0	1	2	2	...
114	0	1	0	1	2	...
115	0	1	0	0	0	...
116	1	1	1	2	0	...
117	0	2	0	0	0	...
118	0	1	0	0	0	...
119	0	1	1	3	0	...
120	0	0	1	1	1	...
121	0	0	1	2	0	...
122	1	1	4	1	0	...
123	1	0	1	1	1	...
124	0	0	5	3	1	...
125	0	0	2	1	0	...
126	0	0	1	1	0	...
127	0	0	2	2	4	...
128	0	1	1	1	0	...
129	0	0	2	1	1	...
130	0	0	1	2	0	...
131	0	1	3	1	0	...

132	1	1	1	1	0	...
133	0	0	2	2	0	...
134	0	0	2	1	0	...

	Poverty_Score	MV_Poverty_Score	Insecure_Housing	WASH_Score	\
0	3	0.000000	0	0	
1	2	0.333333	0	2	
2	1	0.666667	0	1	
3	0	1.000000	1	2	
4	1	0.666667	0	0	
5	1	0.666667	1	1	
6	2	0.333333	0	1	
7	0	1.000000	0	1	
8	0	1.000000	0	0	
9	0	1.000000	0	0	
10	2	0.333333	0	2	
11	2	0.333333	0	0	
12	1	0.666667	0	0	
13	2	0.333333	1	2	
14	2	0.333333	0	1	
15	2	0.333333	0	1	
16	3	0.000000	0	0	
17	2	0.333333	1	3	
18	2	0.333333	1	1	
19	1	0.666667	0	0	
20	2	0.333333	0	1	
21	2	0.333333	0	0	
22	2	0.333333	1	1	
23	2	0.333333	1	1	
24	2	0.333333	0	1	
25	3	0.000000	1	3	
26	3	0.000000	0	3	
27	2	0.333333	1	1	
28	2	0.333333	0	0	
29	2	0.333333	0	1	
..	...	...	...	...	
105	2	0.333333	1	1	
106	1	0.666667	0	0	
107	1	0.666667	0	0	
108	1	0.666667	0	0	
109	3	0.000000	0	2	
110	1	0.666667	0	0	
111	1	0.666667	0	0	
112	3	0.000000	0	0	
113	2	0.333333	0	2	
114	3	0.000000	0	0	
115	3	0.000000	0	2	
116	2	0.333333	0	0	

117	2	0.333333	0	0
118	3	0.000000	0	0
119	3	0.000000	0	2
120	2	0.333333	0	2
121	2	0.333333	0	2
122	3	0.000000	0	0
123	2	0.333333	0	2
124	3	0.000000	1	1
125	2	0.333333	0	0
126	3	0.000000	1	1
127	0	1.000000	0	0
128	2	0.333333	0	0
129	3	0.000000	0	0
130	3	0.000000	0	2
131	2	0.333333	0	2
132	2	0.333333	0	2
133	2	0.333333	0	0
134	2	0.333333	0	0

	WASH_Safe_Score	MV_WASH_Score	MV_WASH_Safe_Score	Water_distance \
0	1	1.00	0.75	0
1	2	0.50	0.50	0
2	1	0.75	0.75	0
3	2	0.50	0.50	0
4	1	1.00	0.75	0
5	2	0.75	0.50	0
6	1	0.75	0.75	0
7	1	0.75	0.75	0
8	0	1.00	1.00	0
9	0	1.00	1.00	0
10	2	0.50	0.50	0
11	0	1.00	1.00	0
12	0	1.00	1.00	0
13	2	0.50	0.50	0
14	1	0.75	0.75	0
15	1	0.75	0.75	0
16	1	1.00	0.75	0
17	4	0.25	0.00	0
18	1	0.75	0.75	0
19	1	1.00	0.75	0
20	1	0.75	0.75	0
21	1	1.00	0.75	0
22	1	0.75	0.75	0
23	1	0.75	0.75	0
24	1	0.75	0.75	0
25	4	0.25	0.00	0
26	3	0.25	0.25	0
27	2	0.75	0.50	0

28	0	1.00	1.00	0
29	1	0.75	0.75	0
..	...	...	...	...
105	1	0.75	0.75	1
106	0	1.00	1.00	1
107	0	1.00	1.00	1
108	0	1.00	1.00	0
109	2	0.50	0.50	1
110	1	1.00	0.75	0
111	0	1.00	1.00	0
112	0	1.00	1.00	0
113	2	0.50	0.50	0
114	1	1.00	0.75	0
115	3	0.50	0.25	0
116	0	1.00	1.00	1
117	0	1.00	1.00	1
118	0	1.00	1.00	0
119	2	0.50	0.50	0
120	2	0.50	0.50	0
121	3	0.50	0.25	0
122	0	1.00	1.00	0
123	2	0.50	0.50	0
124	2	0.75	0.50	0
125	0	1.00	1.00	0
126	1	0.75	0.75	0
127	0	1.00	1.00	0
128	0	1.00	1.00	0
129	0	1.00	1.00	0
130	2	0.50	0.50	0
131	2	0.50	0.50	0
132	2	0.50	0.50	0
133	0	1.00	1.00	0
134	1	1.00	0.75	0

	Water_Time	location
0	0	1
1	0	1
2	0	1
3	0	1
4	0	1
5	0	1
6	0	1
7	0	1
8	0	1
9	0	1
10	0	1
11	0	1
12	1	1

13	0	1
14	0	1
15	0	1
16	0	1
17	1	1
18	0	1
19	0	1
20	0	1
21	0	1
22	0	1
23	0	1
24	0	1
25	0	1
26	0	1
27	0	1
28	0	1
29	0	1
..	...	...
105	1	1
106	1	1
107	1	1
108	0	1
109	0	1
110	0	1
111	1	1
112	0	1
113	0	1
114	0	1
115	0	1
116	1	1
117	0	1
118	0	1
119	0	0
120	0	0
121	0	0
122	0	0
123	0	0
124	0	0
125	0	0
126	0	0
127	0	0
128	0	0
129	0	0
130	0	0
131	0	0
132	0	0
133	0	0
134	0	0

```
[135 rows x 97 columns]>
```

```
In [364]: multivar_model = sm.formula.glm('MV_Roma~Property_documents + Education_level + Family

          print my_data.keys()

          multivar_model.summary()
```

```
Index([u'Survey', u'Community', u'Household_size', u'Time_lived_there',
       u'Head_of_house', u'50+_men', u'50+_women', u'15-49_men',
       u'15-49_women', u'under_15_boys', u'under_15_girls',
       u'literate_men_(+15)', u'literate_women_(+15)', u'Religion', u'Gender',
       u'Age', u'Marital_status', u'Education', u'Occupation', u'Ethnicity',
       u'Drinking_water_sources_available', u'Primary_drinking_water_source',
       u'Primary_non-drinking_water_source',
       u'Who_uses_primary_non-drinking_water_source',
       u'Belief_in_safety_of_primary_drinking_water_source',
       u'Distance_of_primary_drinking_water_source',
       u'Length_of_time_to_collect_water', u'Pay_for_water',
       u'Water_treatment', u'Type_of_water_treatment',
       u'Satisfaction_level_with_water', u'Time_on_household_duties',
       u'Spouse_time_on_household_duties', u'Children_attend_school',
       u'Bathroom', u'Toilet_facility', u'Shared_toilet',
       u'Diarrhea_in_last_year', u'Diarrhea_number_of_people', u'Immunized',
       u'Children_immunized', u'Primary_care_physician', u'Medical_Insurance',
       u'Last_doctor_visit', u'Child_to_doctor', u'Land_for_personal_use',
       u'Animals_owned', u'weekly_expenses_(RON)', u'Property_documents',
       u'Items_owned', u'Electricity', u'Piped__tank_gas',
       u'Decider_for_female_earned_money', u'Conflict_between_Roma_and_non',
       u'Roma_segregated', u'Community_helpful', u'House', u'Roof', u'Floor',
       u'Housing_Score', u'Roma', u'Education_level', u'Family_size',
       u'Marital_status_simple', u'Employment_status', u'Total_men',
       u'Total_women', u'illiterate_men', u'illiterate_women',
       u'WeeklyExpense_perPerson', u'Expense_person', u'tap_water_availabe',
       u'tap_water_available', u'safe_water', u'Time_to_water',
       u'proper_toilet', u'Water_satisfaction', u'Employment_type',
       u'Geography', u'MV_Roma', u'Improved_sanitation', u'Sanitation_two',
       u'Improved_water', u'Modified_WASH_Score', u'Diarrhea',
       u'Healthcare_Score', u'MV_Healthcare_Score', u'Poverty_Score',
       u'MV_Poverty_Score', u'Insecure_Housing', u'WASH_Score',
       u'WASH_Safe_Score', u'MV_WASH_Score', u'MV_WASH_Safe_Score',
       u'Water_distance', u'Water_Time', u'location'],
      dtype='object')
```

```
Out[364]: <class 'statsmodels.iolib.summary.Summary'>
```

```
"""
                 Generalized Linear Model Regression Results
==============================================================================
Dep. Variable:                MV_Roma   No. Observations:                  129
Model:                            GLM   Df Residuals:                      124
Model Family:                Gaussian   Df Model:                            4
Link Function:               identity   Scale:                   0.17306315844
Method:                          IRLS   Log-Likelihood:                -67.354
Date:            Tue, 14 Mar 2017   Deviance:                       21.460
Time:                        12:46:54   Pearson chi2:                     21.5
No. Iterations:                     4
======================================================================================
                          coef    std err          z      P>|z|      [95.0% Conf. Int.]
--------------------------------------------------------------------------------------
Intercept               0.2451      0.146      1.676      0.094        -0.041     0.532
Property_documents      0.0854      0.079      1.084      0.279        -0.069     0.240
Education_level         0.2613      0.082      3.180      0.001         0.100     0.422
Family_size             0.2362      0.078      3.026      0.002         0.083     0.389
Employment_status       0.0505      0.086      0.585      0.559        -0.119     0.220
======================================================================================
"""
```

```
In [365]: multivar_model = sm.formula.glm('Roma~Diarrhea + Improved_water + Insecure_Housing + E


          print my_data.keys()


          multivar_model.summary()
```

```
Index([u'Survey', u'Community', u'Household_size', u'Time_lived_there',
       u'Head_of_house', u'50+_men', u'50+_women', u'15-49_men',
       u'15-49_women', u'under_15_boys', u'under_15_girls',
       u'literate_men_(+15)', u'literate_women_(+15)', u'Religion', u'Gender',
       u'Age', u'Marital_status', u'Education', u'Occupation', u'Ethnicity',
       u'Drinking_water_sources_available', u'Primary_drinking_water_source',
       u'Primary_non-drinking_water_source',
       u'Who_uses_primary_non-drinking_water_source',
       u'Belief_in_safety_of_primary_drinking_water_source',
       u'Distance_of_primary_drinking_water_source',
       u'Length_of_time_to_collect_water', u'Pay_for_water',
       u'Water_treatment', u'Type_of_water_treatment',
       u'Satisfaction_level_with_water', u'Time_on_household_duties',
       u'Spouse_time_on_household_duties', u'Children_attend_school',
       u'Bathroom', u'Toilet_facility', u'Shared_toilet',
       u'Diarrhea_in_last_year', u'Diarrhea_number_of_people', u'Immunized',
       u'Children_immunized', u'Primary_care_physician', u'Medical_Insurance',
       u'Last_doctor_visit', u'Child_to_doctor', u'Land_for_personal_use',
```

```
       u'Animals_owned', u'weekly_expenses_(RON)', u'Property_documents',
       u'Items_owned', u'Electricity', u'Piped__tank_gas',
       u'Decider_for_female_earned_money', u'Conflict_between_Roma_and_non',
       u'Roma_segregated', u'Community_helpful', u'House', u'Roof', u'Floor',
       u'Housing_Score', u'Roma', u'Education_level', u'Family_size',
       u'Marital_status_simple', u'Employment_status', u'Total_men',
       u'Total_women', u'illiterate_men', u'illiterate_women',
       u'WeeklyExpense_perPerson', u'Expense_person', u'tap_water_availabe',
       u'tap_water_available', u'safe_water', u'Time_to_water',
       u'proper_toilet', u'Water_satisfaction', u'Employment_type',
       u'Geography', u'MV_Roma', u'Improved_sanitation', u'Sanitation_two',
       u'Improved_water', u'Modified_WASH_Score', u'Diarrhea',
       u'Healthcare_Score', u'MV_Healthcare_Score', u'Poverty_Score',
       u'MV_Poverty_Score', u'Insecure_Housing', u'WASH_Score',
       u'WASH_Safe_Score', u'MV_WASH_Score', u'MV_WASH_Safe_Score',
       u'Water_distance', u'Water_Time', u'location'],
      dtype='object')
```

```
Out[365]: <class 'statsmodels.iolib.summary.Summary'>
"""
                 Generalized Linear Model Regression Results
==============================================================================
Dep. Variable:                   Roma   No. Observations:                  135
Model:                            GLM   Df Residuals:                      130
Model Family:                Gaussian   Df Model:                            4
Link Function:               identity   Scale:                  0.179985634267
Method:                          IRLS   Log-Likelihood:                -73.255
Date:                Tue, 14 Mar 2017   Deviance:                       23.398
Time:                        12:46:54   Pearson chi2:                     23.4
No. Iterations:                     4
=======================================================================================
                       coef    std err          z      P>|z|      [95.0% Conf. Int.]
---------------------------------------------------------------------------------------
Intercept            0.1458      0.118      1.234      0.217        -0.086      0.377
Diarrhea             0.1302      0.074      1.750      0.080        -0.016      0.276
Improved_water      -0.1914      0.098     -1.960      0.050        -0.383 -4.65e-05
Insecure_Housing    -0.2860      0.089     -3.198      0.001        -0.461     -0.111
Electricity          0.1802      0.122      1.481      0.139        -0.058      0.419
=======================================================================================
"""
```

```
In [366]: my_dataRK.groupby('Roma')['Diarrhea'].value_counts()
```

```
Out[366]: Roma  Diarrhea
          0     0           57
                1           41
          1     1           22
```

```
      0           15
dtype: int64
```

In [367]:
```
my_dataRK['MV_Diarrhoea']=0
my_dataRK['MV_Diarrhoea']=1-my_dataRK['Diarrhea']
```

In [368]:
```
my_dataRK.groupby('MV_Roma')['MV_Diarrhoea'].value_counts()
```

Out[368]:
```
MV_Roma  MV_Diarrhoea
0        0               22
         1               15
1        1               57
         0               41
dtype: int64
```

In [369]:
```
my_dataRK.groupby('MV_Roma')['Improved_water'].value_counts()
```

Out[369]:
```
MV_Roma  Improved_water
0        0                 34
         1                  3
1        0                 78
         1                 20
dtype: int64
```

In [370]:
```
my_dataRK['MV_Improved_water']=0
my_dataRK['MV_Improved_water']=1-my_dataRK['Improved_water']
```

In [371]:
```
my_dataRK.groupby('MV_Roma')['MV_Improved_water'].value_counts()
```

Out[371]:
```
MV_Roma  MV_Improved_water
0        1                    34
         0                     3
1        1                    78
         0                    20
dtype: int64
```

In [372]:
```
my_dataRK.groupby('MV_Roma')['Electricity'].value_counts()
```

Out[372]:
```
MV_Roma  Electricity
0        1              36
         0               1
1        1              85
         0              13
dtype: int64
```

In [373]:
```
my_dataRK['MV_Electricity']=0
my_dataRK['MV_Electricity']=1-my_dataRK['Electricity']
```

In [374]:
```
my_dataRK.groupby('MV_Roma')['MV_Electricity'].value_counts()
```

```
Out[374]: MV_Roma  MV_Electricity
          0        0                 36
                   1                  1
          1        0                 85
                   1                 13
          dtype: int64
```

```
In [375]: my_dataRK.groupby('MV_Roma')['Insecure_Housing'].value_counts()
```

```
Out[375]: MV_Roma  Insecure_Housing
          0        0                   35
                   1                    2
          1        0                   71
                   1                   27
          dtype: int64
```

```
In [376]: multivar_model = sm.formula.glm('MV_Roma~MV_Diarrhoea + MV_Improved_water + Insecure_H


          print my_data.keys()


          multivar_model.summary()
```

```
Index([u'Survey', u'Community', u'Household_size', u'Time_lived_there',
       u'Head_of_house', u'50+_men', u'50+_women', u'15-49_men',
       u'15-49_women', u'under_15_boys', u'under_15_girls',
       u'literate_men_(+15)', u'literate_women_(+15)', u'Religion', u'Gender',
       u'Age', u'Marital_status', u'Education', u'Occupation', u'Ethnicity',
       u'Drinking_water_sources_available', u'Primary_drinking_water_source',
       u'Primary_non-drinking_water_source',
       u'Who_uses_primary_non-drinking_water_source',
       u'Belief_in_safety_of_primary_drinking_water_source',
       u'Distance_of_primary_drinking_water_source',
       u'Length_of_time_to_collect_water', u'Pay_for_water',
       u'Water_treatment', u'Type_of_water_treatment',
       u'Satisfaction_level_with_water', u'Time_on_household_duties',
       u'Spouse_time_on_household_duties', u'Children_attend_school',
       u'Bathroom', u'Toilet_facility', u'Shared_toilet',
       u'Diarrhea_in_last_year', u'Diarrhea_number_of_people', u'Immunized',
       u'Children_immunized', u'Primary_care_physician', u'Medical_Insurance',
       u'Last_doctor_visit', u'Child_to_doctor', u'Land_for_personal_use',
       u'Animals_owned', u'weekly_expenses_(RON)', u'Property_documents',
       u'Items_owned', u'Electricity', u'Piped__tank_gas',
       u'Decider_for_female_earned_money', u'Conflict_between_Roma_and_non',
       u'Roma_segregated', u'Community_helpful', u'House', u'Roof', u'Floor',
       u'Housing_Score', u'Roma', u'Education_level', u'Family_size',
       u'Marital_status_simple', u'Employment_status', u'Total_men',
       u'Total_women', u'illiterate_men', u'illiterate_women',
```

```
 u'WeeklyExpense_perPerson', u'Expense_person', u'tap_water_availabe',
 u'tap_water_available', u'safe_water', u'Time_to_water',
 u'proper_toilet', u'Water_satisfaction', u'Employment_type',
 u'Geography', u'MV_Roma', u'Improved_sanitation', u'Sanitation_two',
 u'Improved_water', u'Modified_WASH_Score', u'Diarrhea',
 u'Healthcare_Score', u'MV_Healthcare_Score', u'Poverty_Score',
 u'MV_Poverty_Score', u'Insecure_Housing', u'WASH_Score',
 u'WASH_Safe_Score', u'MV_WASH_Score', u'MV_WASH_Safe_Score',
 u'Water_distance', u'Water_Time', u'location', u'MV_Diarrhoea',
 u'MV_Improved_water', u'MV_Electricity'],
dtype='object')
```

Out[376]:
```
<class 'statsmodels.iolib.summary.Summary'>
"""
                 Generalized Linear Model Regression Results
==============================================================================
Dep. Variable:                MV_Roma   No. Observations:                  135
Model:                            GLM   Df Residuals:                      130
Model Family:                Gaussian   Df Model:                            4
Link Function:               identity   Scale:                  0.179985634267
Method:                          IRLS   Log-Likelihood:                -73.255
Date:                Tue, 14 Mar 2017   Deviance:                       23.398
Time:                        12:46:55   Pearson chi2:                     23.4
No. Iterations:                     4
=====================================================================================
                        coef    std err          z      P>|z|      [95.0% Conf. Int.]
-------------------------------------------------------------------------------------
Intercept             0.7351      0.099      7.426      0.000         0.541     0.929
MV_Diarrhoea          0.1302      0.074      1.750      0.080        -0.016     0.276
MV_Improved_water    -0.1914      0.098     -1.960      0.050        -0.383 -4.65e-05
Insecure_Housing      0.2860      0.089      3.198      0.001         0.111     0.461
MV_Electricity        0.1802      0.122      1.481      0.139        -0.058     0.419
=====================================================================================
"""
```

In [377]:
```
my_dataRK['MV_Expense']=0
my_dataRK['MV_Expense']=1-my_dataRK['Expense_person']
```
