# Supplementary material for: A survey of quality of life indicators in the Romanian Roma population following the ‘Decade of Roma Inclusion’
Source: F1000Res. 2018 Dec 13;6:1692. Originally published 2017 Sep 15. [Version 3] doi: 10.12688/f1000research.12546.3 (PMC6357989; doi:10.12688/f1000research.12546.3)
Supplement: Supplementary file 4 [file f1000research-6-19000-s0003.tgz › f7135dca-2815-4ea5-9864-684561fe94d7.docx]

**Survey of demographics, satisfaction, water access and disease in Roma settlements throughout Romania**

**Date of interview:**

**Start time:**

**COMMUNITY ID#:**

**Household ID#:**

**INTERVIEWER:**

**Household characteristics**. ***First, I’d like ask some questions about your household.***

1. What is the number of people in this household? _______________
2. How long has your household been living here?

Year: _____________ OR _______________years ago

1. What is the relationship of the respondent to the head of household? _________________________ 1 = Male, 2 = Female, 3 = Male and female together (who is head of household)
2. Number of men 50+ years _______

5. Number of women 50+ years ______

1. Number of men 15-49 years ______
2. Number of women 15-49 years ______
3. Number of men 15 or less years ______
4. Number of women 15 or less years ______
5. Number of literate men (15+ years old) _______
6. Number of literate women (15+ years old) _______
7. What is your religion? Orthodox Christian 1 Catholic Christian 2 Protestant Christian 3 Muslim 4 Other 5 (specify) ____________

**Simplify for R analysis: 1 = Orthodox 2 = Other

**RESPONDANT CHARACTERISTICS. *Now, I’d like to ask you a little bit about yourself and your background.***

1. What is your gender? Male 1 Female 2
2. Do you know how old you are? Yes 1 No 2

If yes, how old are you? ________________ (in years)

1. What is your marital status? Married, living with spouse 1 Married, living separate 2 Living with partner3  Divorced/Separated 4 Widow(er) 5 Single 6
2. What is your highest level of education? No formal school 1 Some primary 2 Primary completed 3 Some secondary 4 Secondary completed 5 Required grades complete 6Traditional High school complete 7 Vocational 8 University/College 9 Post-graduate 10 If less than primary completed, why were you unable to finish primary school? ___________________________
3. Do you work outside of your home? Yes, full time employment 1 Yes, day labor or scavenger 2 No, retired or pensioner 3 No, unemployed 4 No, unemployed with social aid 5 No, Student 6

If yes, what is your occupation? _________________

1. What is your ethnicity? Roma 1 Romanian 2 Rudar 3 Hungarian 4 Other 5

**HOUSEHOLD’S WATER SOURCES and activities. *Next, I’d like to ask you about the water sources used by your household and daily activities.***

1. What type of *drinking* water source do you have access to on a daily basis? (Check all that apply)

Piped water into home 1

Neighbor’s piped water 2

Public protected well 3

Public unprotected well 4

Public hand pump 5

Public outdoor tap 6

Private protected well 7

Private unprotected well 8

River, pond, dam or spring 9

Rainwater collection 10

Private outdoor tap 11

Other 12 (specify)___ (bottled water) _______________

1. Of these *drinking* water sources, which ones do you use frequently (more than 3 times a week)?

Piped water into home 1

Neighbor’s piped water 2

Public protected well 3

Public unprotected well 4

Public hand pump 5

Public tap 6

Private protected well 7

Private unprotected well 8

River, pond, dam or spring 9

Rainwater collection 10

Private Tap 11

Other 12 (specify)__________________

1. What is the main source of water used by your household for other purposes, such as cooking and washing (non-drinking water source)?

Piped water into home 1

Neighbor’s piped water 2

Public protected well 3

Public unprotected well 4

Public hand pump 5

Public tap 6

Private protected well 7

Private unprotected well 8

River, pond, dam or spring 9

Rainwater collection 10

Private Tap 11

Other 12 (specify)__________________

1. Who uses this water source? Adult Men 1 Adult women 2  male children 3 female children 4 All adults 5  All children 6 Everyone 7
2. Do you believe that your primary drinking water source is safe to drink? Yes 1 No 2

Why or why not? ________________________________________________________

1. How far is your primary drinking water source from your home? Meters 1 Kilometers 2 In home 3
2. How long does it take to go there, get water, and come back? No. of minutes ____________

Water in home 1  0-15 minutes 2  15-30 minutes 3 30-45 minutes 4

45-60 minutes 5

1. Do you pay for access to *drinking* water? Yes 1 No 2
2. Do you treat your water in any way to make it safer to drink? Yes 1 No 2

Why or why not? __________________________________________________

1. If yes, what do you usually do to the water to make it safer to drink?

Boil 1

Add bleach/chlorine 2

Strain it through a cloth 3

Use a water filter 4

Solar disinfection (let it sit out in the sun) 5

Let it stand and settle 6

I don’t know 7

Other 8  (specify)____________________________

1. Overall, are you satisfied with your current drinking water availability?

1 Generally satisfied

2 Somewhat dissatisfied

3 Very dissatisfied

4 Don’t know / No response

Why are you satisfied / Why are you not satisfied?

Satisfied: (Do not prompt- check all mentioned):

01 Takes little time to get water

02 Water source is close by

03 Adequate water for home use

04 Water can be collected by children

05 Water supply is not costly

06 Water supply is safe for drinking

07 Water supply is reliable

08 Good management of system

09 Other (specify):

________________________________________________

Not Satisfied: (Do not prompt-check all mentioned):

01 Takes too much time to get water

02 Water source is too distant

03 Not enough water for home use

04 Water can’t be collected by children

05 Water supply is too costly

06 Water supply is unsafe

07 Water supply is unreliable

08 Poor management of system

09 Other (specify):

________________________________________________

1. How much time each day do you spend on household chores? ________________ (hrs, mins)
2. How much time each day does your spouse spend on household chores (if married)? _____________ (hrs, mins)
3. Do your children (if any) attend school? Yes 1 No 2 No children 3 Why or why not?
4. Open-ended question: Can you describe a ‘typical’ day in your life?

________________________________________________________________________________________________________________________________________________________________________________________________________________________________________________________________________________________________________________________________________________________________________________________________________________________________________________________________________________________________________________________________________________________________________________________________________________________________________________________________________________

**WELLNESS. *Next, I’d like to ask you about the hygiene and health of you and your family members.***

1. Do you have a bathroom?

Yes, in the house = 1

Yes, outside = 2

No = 3

2***.*** What kind of toilet facility do members of your household usually use?

Indoor flush to piped sewer 1

Indoor flush to septic tank 2

Outdoor flush to septic tank 3

Outdoor flush to pit latrine/hole 4

Composting toilet 5

Bucket 6

Hanging toilet 7

No facilities / open bush or field 8

Other 11(specify)_____________________________

3. Do you share this facility with other households? Yes 1 No 2

If yes, how many other households? ___________

4. As best as you can remember, have you or your family members had a diarrheal illness in the last:

year: Yes 1 No 0

Six months: Yes 3 No 4

Three months: Yes 5 No 6

Month: Yes 7 No 8

Two weeks: Yes 9 No 10

Not at all for any of the above time periods = 11

5. If ‘yes’ to any of #3, which family member was ill (adult male, adult female, male child, female

child? _______________________ (list as many as are relevant)

6. How many times in the same time period (year, months, weeks, etc) has this individual had diarrheal

symptoms? ______________________ (obtain info for each individual listed in #4)

7. Have you ever received an immunization (shot) of any kind against an illness? Yes 1 No 0

If yes, do you remember which one? __________________________ (list all)

8. Have your children received an immunization (shot) of any kind against an illness? Yes 1 No 2

No children 3

If yes, do you remember which one? __________________________ (list all)

9. Do you have access to a primary care physician? Yes 1 No 0

If no, would you take your children to a physician if you had access to one? Yes 1 No 2

10. Do you have medical insurance Yes =1, No = 0

11. When was the last time you went to the doctor?

A year ago, = 1

Six months ago, = 2

Three months ago, = 3

One month ago, = 4

Two weeks ago, = 5

12. Would you take your child to the doctor if he or she was ill?

Every time =1

Sometimes =2

Never = 3 Why not?

**wealth and assets. *Next, I’d like to ask you additional questions about your household.***

1. How much land do you use to grow your own food? ________________ m^3^

2. How many of each of the following animals does your household currently own?

1. Cattle ………………. ______ 6. Goats ……………. ________

2. Sheep ……………… ______ 7. Poultry …………. ________

3. Horses ……………... ______ 8. Rabbits …………. ________

4. Donkeys …………… ______ 9. Other ……………________

5. Pigs ______

3. About how much does your household spend PER WEEK on regular expenses like food, transport, school fees, etc?

*(***Help the respondent calculate their expenses if necessary) CFA* _________________­­­­______ /week

4. Do you have proof of ownership/property documents for your home? Yes = 1 No = 2

5. Does anyone in your household own the following items? *(***Read list and mark answer for each item.)*

|  | Yes | No |  | Yes | No |
| --- | --- | --- | --- | --- | --- |
| 1. Radio? | 1 | 0 | 1. Car | 1 | 0 |
| 1. TV? | 1 | 0 | 1. Plow? | 1 | 0 |
| 1. Generator? | 1 | 0 | 1. Tractor? | 1 | 0 |
| 1. Bicycle? | 1 | 0 | 1. Cart? | 1 | 0 |
| 1. Computer?   11. Refrigerator | 1  1 | 0  0 | 10. Mobile Phone  12. Stove | 1  1 | 0  0 |

6. Does your household have electricity service?

1 Yes

2 Yes, but disconnected now

3 No

7. Do you have gas (piped)?

Yes =1

Yes, but disconnected now = 2

No, we use tanks =3

No, we use fire =4

8. In your household, WHO DECIDES how the income earned by WOMEN will be used?

1 Husband only

2 Wife only

3 Husband and wife TOGETHER

4 Husband and wife SEPARATELY

5 All adults

6 Children

7 All members of the family

8 Other (specify)______________________________________

9. Do you think there are conflicts between Roma and non-Roma in your community?

1 Yes, why______________

2 Sometimes

3 No

4 Don’t know/ no answer

10. Do you think the Roma community is segregated or separated from the majority?

1 Yes, why______________

2 Sometimes

3 No

4 Don’t know/ no answer

11. Would you say most people in this community are willing to help you if you need it?

1 Yes

2 Maybe

3 No

4 Don't know / No answer

12. ****Observe if possible:*

| 1. *What are the walls of the house made of primarily?* | 1. *What is the roof of the house made of primarily?* | 1. *What is the floor of the house made of primarily* |
| --- | --- | --- |
| 1 Stone / Brick / Cement  2 Wood  3 Clay / Mud  4 Metal  5 Sticks  6 Plastic  7 Other  8 Don't know | 1 Metal / Corrugated metal  2 Thatch / Branches  3 Cement / Concrete  4 Wood  5 Plastic sheeting  6 Other  7 Don't know | 1 Cement / Concrete  2 Tile  3 Wood  4 Mud / sand / dirt  5 Cow dung  6 Other  7 Don't know |

13. ****Observe if possible:*

*Are toilet facilities upwind or downwind of water sources? ____________________________*

*If not in home, approximately how far from home is the water source? ___________________*

*Is there stagnant water or waste visible on the streets? _______________________________*

**** This is the end of our interview. THANK YOU for your time. ****

*(***Interviewer: Please record any additional notes here)*

____________________________________________________________________________________________________

____________________________________________________________________________________________________

____________________________________________________________________________________________________
