# Supplementary material for: A survey of quality of life indicators in the Romanian Roma population following the ‘Decade of Roma Inclusion’
Source: F1000Res. 2018 Dec 13;6:1692. Originally published 2017 Sep 15. [Version 3] doi: 10.12688/f1000research.12546.3 (PMC6357989; doi:10.12688/f1000research.12546.3)
Supplement: Supplementary file 5 [file f1000research-6-19000-s0004.tgz › 8f4bdbdc-ef4b-4fcf-9fa3-fbcb58c1c643.docx]

**Chestionar cu privire la demografie, satisfacție, acces la apă și boală în comunitățile de romi din România**

Data interviului:

Ora la care începe interviul:

Codul comunității:

Codul gospodăriei:

Numele persoanei care realizează interviul:

**Caracteristici ale gospodăriei. În primul rând, aș vrea să vă adresez câteva întrebări cu privire la gospodăria dumneavoastră.**

1. Care este numărul persoanelor care locuiesc în gospodărie? _______________
2. De cât timp există această gospodărie aici?

Anul: _____________ SAU_______________ANI

1. Care este relația respondentului cu capul gospodăriei? _________________________
2. Numărul bărbaților din gospodărie care au peste 50 de ani _______

5. Numărul femeilor din gospodărie care au peste 50 de ani ______

1. Numărul bărbaților din gospodărie cu vârste cuprinse între 15-49 ani______
2. Numărul femeilor din gospodărie cu vârste cuprinse între 15-49 ani ______
3. Numărul bărbaților din gospodărie care au mai puțin de 15 ani ______
4. Numărul femeilor din gospodărie care au mai puțin de 15 ani ______
5. Numărul bărbaților din gospodărie peste 15 ani care știu să scrie și să citească _______
6. Numărul femeilor din gospodărie peste 15 ani care știu să scrie și să citească _______
7. Care este religia dumneavoastră? Ortodoxă 1 Catolică 2 Protestantă 3 Musulmană 4 Altă 5 (specificați) ____________

**Caracteristicile respondenților. Acum, aș vrea să pun câteva întrebări despre dumneavostră și experiențele anterioare.**

1. Care este genul dumneavoastră? Bărbatul 1 Femeie 2
2. Care este vârsta dumneavoastră?

________________ Nu știu

1. Care este statutul dumneavoastră marital? What is your marital status?

Căsătorit / ă, locuiescu cu soția / soțul 1  Căsătorit / ă, locuim separate 2  Locuiesc cu partenerul/ a 3  Divorțat / ă 4  Văduv / ă 5 Singur / ă 6

1. Care este cea mai înaltă formă de educație absolvită?

Fără școală 1 Câteva clase primare 2 Școala primară completă 3 Câteva clase din școala generală 4 Școala generală completă 5 10 clase terminate 6 12 clase terminate 7

12 clase terminate cu Bacalaureat 8 Școală profesională 9  Facultate ciclul licență 10  Studii post-universitare 11

Dacă nu ați terminat școala general, de ce? ___________________________

1. Care este ocupația dvs.? _________________

Casnic/ă

1. Sunteți de etnie romă? Da 1 Nu 2

**SURSELE DE APĂ DIN GOSPODĂRIE ȘI ACTIVITĂȚI. În continuare, aș vrea să vă întreb despre sursele de apă folosite zilnic în gospodărie**

1. La ce tip de sursă de apă potabilă aveți acces? (Răspuns multiplu)

Apă curentă în casă 1

Apă curentă de la un vecin 2

Fântână protejată public 3

Fântână neprotejată 4

Robinet public 5

Râu, baraj 6

Apă colectată după ploaie 7

Altele 8 (specificați)__________________

1. Din aceste surse de apă potabilă, pe care o folosiți cel mai des

Apă curentă în casă 1

Apă curentă de la un vecin 2

Fântână protejată public 3

Fântână neprotejată 4

Robinet public 5

Râu, baraj 6

Apă colectată după ploaie 7

Altele 8 (specificați)__________________

1. Care este principal sursă de apă folosită în gospodărie pentru alte activități, precum gătit sau spălat pe mâini?

Apă curentă în casă 1

Apă curentă de la un vecin 2

Fântână protejată public 3

Fântână neprotejată 4

Robinet public 5

Râu, baraj 6

Apă colectată după ploaie 7

Altele 8 (specificați)__________________

1. Cine folosește această sursă de apă, pentru gătit și spălat pe mâini (răspuns multiplu)? Bărbat adult 1 Femeie adult 2  Copil băiat 3  Copil fată 4
2. Considerați că sursa primară de apă este potabilă? Da 1 Nu 2

De ce dau sau de ce nu? ________________________________________________________

1. Cât de departe este sursa primară de apă potabilă de casa dvs.? Metri 1 Kilometri 2
2. Cât timp vă ia să mergeți să luați apă și să vă întoarceți înapoi acasă? Nr. minute ____________

Am apă în curte sau în apropiere 1  Nu știu 2

1. Trebuie să plătiți pentru acces la apă potabilă? Da 1 Nu 2
2. Tratați apa în vreun fel pentru a vă asigura că este potabilă? Da 1 Nu 2

De ce da sau de nu? __________________________________________________

1. Dacă da, ce faceți de obicei pentru a vă asigura că apa este potabilă?

O fierbeți 1

Adăugați Clor 2

O strecurați printr-o pânză sau tifon 3

Folosiți un filtru pentru apă 4

O lăsați să stea și să se liniștească 6

Nu știu 7

Altceva 8  (specificați)____________________________

1. În general, sunteți mulțumit / ă de accesul pe care îl aveți la apă potabilă?

1 În general sunt mulțumit/ă

2 Oarecum nemulțumit/ă

3 Foarte nemulțumit/ă

4 Nu știu / Nu răspund

De ce sunteți mulțumit/ă? De ce sunteți nemulțumit/ă?

Mulțumit/ă: (Do not prompt- check all mentioned):

01 Îmi ia puțin timp să ajung la apă

02 Sursa de apă este aproape

03 Apa este suficientă pentru realizarea activităților zilnice

04 Apa poate fi adusă de copii

05 Alimentarea cu apă nu este costisitoare

06 Sursa de apă este potabilă

07 Sursa de apă este de încredere

08 Autoritățile locale au grijă de rețeaua de apă potabilă

09 Altceva (specificați):

________________________________________________

Nemulțumit/ă: (Do not prompt-check all mentioned):

01 Ne ia foarte mult timp să ajungem la apă

02 Sursa de apă se află la distanță de gospodărie

03 Apa nu este suficientă pentru realizarea activităților zilnice

04 Apa nu poate fi adusă de copii

05 Alimentarea cu apă este prea costisitoare

06 Sursa de apă nu este potabilă

07 Sursa de apă este de încredere

08 Autoritățile locale nu au grijă de rețeaua de apă potabilă

09 Altceva (specificați):

________________________________________________

1. Cât timp petreceți zilnic realizând treburi casnice? ________________ (ore, minute)
2. Cât timp petrece zilnic soțul / soția / partenerul pentru realizarea treburilor casnice? (dacă e cazul) _____________ (ore, minute)
3. În cazul în care aveți copii, acelștia merg la școală? Da 1 Nu 2 De ce da sau de ce nu?

________________________________________________

1. Întrebare deschisă: Puteți descrie o zi obișnuită din viața dumneavoastră?

________________________________________________________________________________________________________________________________________________________________________________________________________________________________________________________________________________________________________________________________________________________________________________________________________________________________________________________________________________________________________________________________________________________________________________________________________________________________________________________________________________

**Sănătate and bunăstare. În continuare, aș vrea să vă întreb despre igiena și sănătatea dvs. și a membrilor familiei dvs.**

1. Aveți baie?

Da, în locuință

Da, in fara locuinței

Nu

1. Ce fel de toaletă este disponibila în gospodăria dvs.?

Toaletă în interiorul locuinței conectată la canalizare

Toaletă în interiorul locuinței conectată la fosă septică

Toaletă în curte conectată la fosa septică

Toaletă în curte

Toaletă ecologică (compost)

Găleată

Altceva (specificați)_______________________

Nu există toaletă

1. Alte persoane din alte gospodării folosesc aceeași toaletă? Da 1 Nu 2

Dacă da, câte persoane? ___________

1. Dvs. sau membrii din familia dvs. ați suferite de dizenterie (boli diareice) în: As best as you can remember, have you or your family members had a diarrheal illness in the last:

Ultimul an: Da 1 Nu 2

Ultimele 6 luni: Da 1 Nu 2

Ultimele 3 luni: Da 1 Nu 2

Ultima lună: Da 1 Nu 2

Ultimele 2 săptămâni: Da 1 Nu 2

1. Dacă da la oricare din răspunsurile de la întrebarea 4, care membru al familiei a fost bolnav? (bărbat, femeie, băiat, fată) _______________________ (îi scrieți pe toți cei relevanți)
2. De câte ori a avut persoana simptome de dizenterie (diaree) în perioada menționată la întrebarea nr. 4? ______________________________________________________________________________(se completează pentru fiecare membru al familiei)
3. Ați fost vaccinat vreodată? Da 1 Nu 2

Dacă da, vă amintiți care __________________________ (se notează toate)

1. Copilul dvs. a făcut vreun vaccin? Da 1 Nu 2

Dacă da, vă amintiți care __________________________ (se notează toate)

1. Sunteți inscris/ă la medical de familie? Da 1 Nu 2
2. Aveți asigurare medicală? Da 1 Nu 2
3. Când ați fost ultima dată la medic?

Ultimul an: Da 1 Nu 2

Ultimele 6 luni: Da 1 Nu 2

Ultimele 3 luni: Da 1 Nu 2

Ultima lună: Da 1 Nu 2

Ultimele 2 săptămâni: Da 1 Nu 2

1. Dacă aveți copii, cât de des îi duceți la doctor dacă sunt bolnavi?

De fiecare dată

Uneori

Niciodată; De ce? _________________________________;

Aveți acces la un medic? Da 1 Nu 2

Dacă ați avea acces, l-ați duce la medic? Da 1 Nu 2

1. La ce distanță se află cel mai apropiat spital? ________________km

**PROPRIETĂȚI ȘI BUNURI. În continuare, aș dori să vă adresez câteva întrebări suplimentare cu privire la această gospodărie.**

1. Cât pământ folosiți pentru cultivarea propriei hrane? ________________m^3^
2. Câte animale din cele menționate mai jos dețineți în gospodărie?

1. Bovine ………………. ______ 6. Capre…………….________

2. Oi……………… ______ 7. Păsări de curte…………..________

3. Cai……………... ______ 8. Iepuri………….________

4. Măgari…………… ______ 9. Altele……………________

5. Porci______

1. Aproximativ, cât cheltuiți în gospodărie săptămânal pentru cheltuieli cu mâncare, transport, etc.?

*(***Ajutați respondentul să calculeze suma, dacă este nevoie) _*________________­­­­_LEI /săptămână

1. Dețineți acte de proprietate pentru locuința familiei dvs.? Da 1 Nu 2
2. Aveți în gospodărie următoarele? *(***Citește toată lista și marcați răspunsul pentru fiecare item)*

|  | Yes | No |  | Yes | No |
| --- | --- | --- | --- | --- | --- |
| 1. Radio | 1 | 0 | 1. Mașină | 1 | 0 |
| 1. TV | 1 | 0 | 1. Plug | 1 | 0 |
| 1. Generator | 1 | 0 | 1. Tractor | 1 | 0 |
| 1. Bicicletă | 1 | 0 | 1. Căruță | 1 | 0 |
| 1. Computer | 1 | 0 | 1. Mobile Phone | 1 | 0 |
| 1. Frigider | 1 | 0 | 1. Aragaz | 1 | 0 |

1. Aveți electricitate?

1 Da

2 Da, dar nu suntem conectați momentan

3 Nu

1. Aveți acces la gaze?

1 Da

2 Da, dar nu suntem conectați momentan

3 Nu

1. În gospodăria dvs., cine decide cum se va cheltui venitul obținut de femeie?

1 Doar bărbatul

2 Doar femeia

3 Bărbatul și femeia împreună

4 Bărbatul și femeia separat

5 Toți adulții

6 Copiii

7 Toți membrii familiei

8 Altcineva (specificați)______________________________________

1. Există conflicte între romi și neromi (români, maghiari sau alte comunități)

1 Da; De ce? ____________________________________

2 Uneori

3 Nu

1. Comunitatea de romi este segregată de cea de majoritari? Da 1 Nu 2

1. Considerați că cei mai mulți oameni din comunitate v-ar ajuta dacă ați avea nevoie?

1 Da

2 Poate

3 Nu

4 Nu știu / Nu răspund

9. ****Observați dacă este posibil:*

| 1. *Din ce sunt făcuți pereții casei?* | 1. *Din ce este făcut acoperișul casei?* | 1. *Din ce este făcută podeaua casei?* |
| --- | --- | --- |
| 1 Stone / Brick / Cement  2 Lemn  3 Chirpici / Pământ  4 Metal  5 Plastic  6 Altceva  7 Nu știu | 1 Metal  2 Paie  3 Ciment / Beton  4 Lemn  5 Plastic  6 Altceva  7 Nu știu | 1 Ciment / Beton  2 Gresie  3 Lemn  4 Pământ  5 Altceva  6 Nu știu |

19. *** *Observați dacă este posibil:*

*Toaletele sunt în amonte sau în aval de râu? ____________________________*

*Dacă nu este în curte, cât de departe de casă este sursa de apă? ___________________*

*Există apă stătătoare / bălți în fața casei sau deșeuri vizibile pe stradă? _____________________*

**** Acesta este finalul interviului. Vă mulțumim.*

*(***Puteți nota alte informații adiționale aici)*

____________________________________________________________________________________________________

____________________________________________________________________________________________________

___________________________________________________________________________________________________
