# Supplementary material for: A survey of quality of life indicators in the Romanian Roma population following the ‘Decade of Roma Inclusion’
Source: F1000Res. 2018 Dec 13;6:1692. Originally published 2017 Sep 15. [Version 3] doi: 10.12688/f1000research.12546.3 (PMC6357989; doi:10.12688/f1000research.12546.3)
Supplement: Peer review history

# Peer review: Reviewer response for version 3

**DOI:** 10.5256/f1000research.19000.r41911

**Published:** 2019-01-31

**Authors:** Pilar Carrasco-Garrido

**Affiliations:** 1 Medical Microbiology and Immunology and Nursing Department, King Juan Carlos University, Madrid, Spain

This is a relevant manuscript from a public health standpoint. In this version after reviewing and incorporating the comments of the different reviewers, quality has improved.

 Methodological analysis has been extended. The discussion of the results and their limitations are now properly defined, despite the limitation of the sample size.

I confirm that I have read this submission and believe that I have an appropriate level of expertise to confirm that it is of an acceptable scientific standard.

# Peer review: Reviewer response for version 3

**DOI:** 10.5256/f1000research.19000.r41912

**Published:** 2019-01-03

**Authors:** Martin McKee

**Affiliations:** 1 European Centre on Health of Societies in Transition, London School of Hygiene and Tropical Medicine, London, UK

This is almost entirely a new paper, with very extensive additional analysis. This recognizes the previously noted limitations but uses a modelling approach to address them, taking the paper in a somewhat different direction. While it is important to continue to view the paper as a methodological rather than empirical paper, seen in this light I feel it is much improved. I would prefer that you received comments from someone with greater expertise in the methods used than me, but as far as I can see it now addresses my previous concerns.

I confirm that I have read this submission and believe that I have an appropriate level of expertise to confirm that it is of an acceptable scientific standard.

# Peer review: Reviewer response for version 2

**DOI:** 10.5256/f1000research.15471.r31657

**Published:** 2018-04-19

**Authors:** Ciprian Marius Ceobanu

**Affiliations:** 1 Faculty of Psychology and Education Sciences, Department of Psychology, Alexandru Ioan Cuza University, Iași, Romania

Despite the small improvements there still remain important issues.

 The comparison with Burkina-Faso is completely uninspired and demonstrate that the author(s) is not aware at all of the whole image related to Roma population.

 Another important issue is related to the extremely small size of the sample. Only 135 persons (on average 27 from each region) is extremely unsignificant from a statistical point of view. Also, there is a large number of Roma population that came from Romania and currently live in different EU countries, on a long-term basis; are they included in the same population?

 Again, the paper still remains a collection of results from official statistics without adding anything new or relevant.

I confirm that I have read this submission and believe that I have an appropriate level of expertise to confirm that it is of an acceptable scientific standard, however I have significant reservations, as outlined above.

# Peer review: Reviewer response for version 2

**DOI:** 10.5256/f1000research.15471.r32678

**Published:** 2018-04-03

**Authors:** Pilar Carrasco-Garrido

**Affiliations:** 1 Medical Microbiology and Immunology and Nursing Department, King Juan Carlos University, Madrid, Spain

Although the objectives are adequate and the subject is relevant, the important limitations of the study are still there.

 Possibly, the small sample size is a problem. This aspect is a methodological problem that can lead to conclusions in the study that are not adequate.

 Sorry but, I do not recommend the acceptance of the article.

I confirm that I have read this submission and believe that I have an appropriate level of expertise to state that I do not consider it to be of an acceptable scientific standard, for reasons outlined above.

# Peer review: Reviewer response for version 2

**DOI:** 10.5256/f1000research.15471.r31658

**Published:** 2018-03-22

**Authors:** Martin McKee

**Affiliations:** 1 European Centre on Health of Societies in Transition, London School of Hygiene and Tropical Medicine, London, UK

While the authors have added some clarity, I remain concerned about the small sample size and other limitations. I don't think they have enough data to draw any conclusions and it is not obvious to me that it has been rewritten as a proof of concept paper. For example, the abstract has, in the conclusions "This paper demonstrates..." whereas I really don't think it does (although in fact, what is concluded is quite well known). I still think it could be revised to be much less ambitious.

I confirm that I have read this submission and believe that I have an appropriate level of expertise to confirm that it is of an acceptable scientific standard, however I have significant reservations, as outlined above.

# Peer review: Reviewer response for version 1

**DOI:** 10.5256/f1000research.13585.r28652

**Published:** 2018-01-02

**Authors:** Pilar Carrasco-Garrido

**Affiliations:** 1 Medical Microbiology and Immunology and Nursing Department, King Juan Carlos University, Madrid, Spain

This is a relevant manuscript from a public health standpoint because one of the main contributions of the present work is to determine quality of life indicators in the Romanian Roma, but methodologically it has significant shortcomings:

- The comparison between the population of Burkina Faso and the population of Romania is not adequate. They are very different populations. This aspect is a methodological problem.
- Discussion of the results is limited. Some aspects are not adequately discussed.
- There is no information about the survey's non-response rate.
- Few references and some of them unrelated to the purpose of the study. It does not seem correct to incorporate a press article as a reference.

Sorry but, I do not recommend the indexing of the article.

I confirm that I have read this submission and believe that I have an appropriate level of expertise to state that I do not consider it to be of an acceptable scientific standard, for reasons outlined above.

# Peer review: Reviewer response for version 1

**DOI:** 10.5256/f1000research.13585.r26652

**Published:** 2017-12-12

**Authors:** Martin McKee

**Affiliations:** 1 European Centre on Health of Societies in Transition, London School of Hygiene and Tropical Medicine, London, UK

The introduction is well written and provides a good overview of the situation facing the Roma population. There are a few more recent references that could be included, such as an evaluation of the Decade of Roma inclusion in Hungary but, in general, the authors have found most of the relevant information.

 The fundamental challenge facing anyone doing research among the Roma population in this region is how to develop a sampling frame. There are numerous methodological problems, in particular varying degrees of assimilation (see, for example, work by K Kosa). Previous studies, such as that by the UNDP or in Hungary, have used Roma communities, identifiable by their socio economic and physical characteristics, while recognising that this is imperfect. However, this paper would benefit from a more detailed description of the communities from which the samples were drawn, in particular, how they relate to Romania as a whole. Given that, in many parts of Romania, Roma live in distinct settlements, separate from the Romanian population, even within individual villages, could the authors comment on any implications that their sampling strategy had for generalisability?

 Given the high levels of distrust that many Roma, justifiably, have, some studies have sought to ensure involvement of Roma fieldworkers, or at least, involvement of community leaders. Can the authors comment on what measures they took in this regard?

 The greatest problem in this paper is the very small sample size. Overall, less than 100 Roma respondents were included and only 37 non-Roma. Given the numerous problems involved in sampling in a study such as this, this is really far too few from which to draw any meaningful conclusion. This is noted in the limitations but I’m not really convinced that a study of this size can be regarded as much more than a pilot. I would suggest that it is described in this way, with many more caveats than there are at present.

 Minor points:

 I’m not sure that it is appropriate to use the words of Soviet rule for the countries of south-eastern Europe. Arguably, Romania was one of the most independent of the Soviet bloc states.

I confirm that I have read this submission and believe that I have an appropriate level of expertise to confirm that it is of an acceptable scientific standard, however I have significant reservations, as outlined above.

# Peer review: Reviewer response for version 1

**DOI:** 10.5256/f1000research.13585.r26651

**Published:** 2017-11-06

**Authors:** Ciprian Marius Ceobanu

**Affiliations:** 1 Faculty of Psychology and Education Sciences, Department of Psychology, Alexandru Ioan Cuza University, Iași, Romania

The main issue is related to the size of the sample and its geographical distribution. It is hard to generalize over the entire Roma minority the conclusions of the study even if the statistical approach is appropriate.

 The geographical distribution of the Roma population is pretty different over the Romanian national territory. The lack of indication of the geographical area of the subjects of the sample is a flaw. Another issue regards the random walk method for sampling which, in our opinion, is not representative for the entire Roma population. Maybe a multilevel sampling would be more appropriate than a simple random walk.

 Despite the fact that the conclusions of the study are correct, these are pretty well known to all levels; also these are commonplaces that were specified in the documents of Decade of Roma Inclusion as directions for future action. There are a lot of significant reports that draw the same conclusions (see The World Bank documents for instance ^1^). From this point of view, there is no original approach to the Roma problem in Romania.

 The conclusion following the Decade of Roma Inclusion is that despite the efforts that there were made, there still remain lots of issues regarding the integration of the Roma population. Also,solving great structural problems of Romania will certainly improve the Roma population situation.

I confirm that I have read this submission and believe that I have an appropriate level of expertise to confirm that it is of an acceptable scientific standard, however I have significant reservations, as outlined above.

## References

[1] : Diagnostics and Policy Advice on the Integration of Roma in Romania [Romanian]. *World Bank Group*.2014;
